# Risk of developing pre-diabetes or diabetes over time in a cohort of Mexican health workers

**DOI:** 10.1371/journal.pone.0229403

**Published:** 2020-03-25

**Authors:** Yvonne N. Flores, Samantha Toth, Catherine M. Crespi, Paula Ramírez-Palacios, William J. McCarthy, Arely Briseño-Pérez, Víctor Granados-García, Jorge Salmerón

**Affiliations:** 1 Unidad de Investigación Epidemiológica y en Servicios de Salud, Delegación Morelos, Instituto Mexicano del Seguro Social, Cuernavaca, Morelos, México; 2 UCLA Department of Health Policy and Management and Kaiser Permanente Center for Health Equity, Fielding School of Public Health, Los Angeles, California, United States of America; 3 UCLA Cancer Prevention and Control Research Center, Fielding School of Public Health and Jonsson Comprehensive Cancer Center, Los Angeles, California, United States of America; 4 UCLA Department of Biostatistics, Fielding School of Public Health, Los Angeles, California, United States of America; 5 Unidad de Investigación Epidemiológica y en Servicios de Salud- Área Envejecimiento, Instituto Mexicano del Seguro Social, Ciudad de México, México; 6 Centro de Investigación en Políticas, Población y Salud, Universidad Nacional Autónoma de México, Ciudad de México, México; Mexican Social Security Institute, MEXICO

## Abstract

**Aim:**

To determine the association between known risk factors (e.g., obesity, metabolic syndrome and its components) and the development of pre-diabetes or diabetes over time in a cohort of Mexican health workers.

**Methods:**

Participants in the Mexican Health Worker Cohort Study with complete information at two waves of data collection, 2004–2006 (W1) and 2011–2013 (W2), were included in the analysis (n = 1,174). Multivariable binary and multinomial logistic regression were used to examine the cross-sectional associations between specific risk factors and diabetes status (diabetes, pre-diabetes, or neither) at W1 and the longitudinal associations between changes in risk factors and progression of diabetes status from W1 to W2, respectively.

**Results:**

Mean time between waves was 7.0 years (SD 1.1). Prevalence of pre-diabetes and diabetes was 16% and 10% at W1 and increased to 30% and 16% at W2, respectively. The cross-sectional prevalence of pre-diabetes and diabetes was significantly higher among men, participants over the age of 45 years, and individuals who were overweight or obese or had metabolic syndrome (MS), three or more components of the MS, elevated alanine aminotransferase (ALT) levels, or elevated uric acid. In longitudinal analyses, remaining obese or gaining weight between waves was associated with an increased risk of developing pre-diabetes. A greater risk of developing pre-diabetes or diabetes was also observed among individuals who either maintained or acquired MS, elevated ALT, or elevated uric acid (only for diabetes) from W1 to W2.

**Conclusions:**

Weight gain and acquiring or maintaining MS, elevated ALT levels, or elevated uric acid were associated with a significant risk of developing pre-diabetes or diabetes. Our findings, especially in the context of the obesity epidemic in Mexico, point towards an urgent need for initiatives to help reduce excess weight in order to avert future cases of pre-diabetes and diabetes.

## Introduction

In Mexico, diabetes is the second most common cause of death, and the leading cause of disability [1,2]. From 1990 to 2015, the percentage of deaths attributed to this disease more than doubled from 6.1% to 15% [3,4]. In 2017, an estimated 106,000 people died from diabetes in Mexico [1], and many of these lives were shortened by up to 10 years [4]. Nearly 15 million Mexican adults were estimated to have the disease in 2017, and that number is projected to increase to 19 million by 2045 [5,6].

Family history and hereditary factors [7] are associated with an increased likelihood of developing diabetes, but additional risk factors, such as environmental and lifestyle choices, can also play a strong role [8–13]. Some of the known risk factors include: high blood pressure, age ≥45, physical inactivity, metabolic syndrome, and obesity; with obesity recognized as the strongest modifiable risk factor [14–20]. Strong associations have also been observed between diabetes and low high-density lipoprotein-cholesterol (HDL-C), high triglycerides, and abnormal levels of alanine aminotransferase (ALT) or serum uric acid [21–26].

The prevalence of overweight and obesity in Mexico is among the highest in the world [27]. In 2016, the National Survey of Health and Nutrition (ENSANUT) determined that an estimated 72.5% of Mexican adults are overweight or obese [28], and that growth in the prevalence of obesity had plateaued in urban areas but continues to rise in rural areas [28]. The surge in weight gain is attributed to changes in lifestyle commonly observed in low- and middle-income countries that have experienced recent advances in per capita socioeconomic resources. [29,30] This increased income has resulted in a higher consumption of calorie-dense, nutrient-poor, low-fiber foods and a corresponding decrease in the consumption of fresh fruits and vegetables, and minimally processed grains and legumes [31**]**. Globalization, urbanization and increased marketing by multinational food companies have provided low-income populations in Mexico with increased exposure to ultra-processed foods that are high in calories, saturated fat and sugar, and low in dietary fiber and nutritional value [32]. This increase in pro-inflammatory calories combined with decreased physical activity and a more sedentary lifestyle have increased the prevalence of excess body fat and metabolic diseases in the last 50 years [3,33–35].

Diabetes is characterized by pancreatic failure to produce enough insulin to keep blood glucose levels in the healthy range. If maintained over time, high blood glucose can result in adverse health effects such as diabetic retinopathy, neuropathy, heart disease, stroke, chronic kidney disease (CKD), sexual impotence and depression [36]. Diabetes is a progressive disease that is preceded by a state of pre-diabetes, which is generally defined as blood glucose levels above the normal range but not high enough to be diabetic (i.e., 100–125 mg/dl for fasting glucose) [37]. In general, the transition from pre-diabetes to diabetes is preceded by increasing insulin resistance and pancreatic beta cell dysfunction [38–40]. Pre-diabetes is linked to a greater risk of developing diabetes and is also associated with a higher risk of cardiovascular disease, CKD, and ocular abnormalities [41–45].

There are limited data regarding the prevalence of pre-diabetes in Mexico, with smaller studies providing estimates ranging from 14% to 47% [46–48], and another study with a high-risk population reporting a prevalence as elevated as 74% [49]. In the United States (U.S.), an estimated 84 million American adults, more than 1 in 3, have pre-diabetes, yet only about 10% are aware they have the condition [50]. Worldwide, between 5–10% of individuals with pre-diabetes go on to develop diabetes each year, and nearly 70% will eventually get the disease [51].

The pathophysiology of diabetes is complex and varied, with disease rates differing by race and ethnicity. Latinos have some of the highest rates of diabetes and obesity, which has been attributed to cultural, social and lifestyle differences, as well as genetic factors [52,53]. The prevalence of diabetes in the U.S. is highest among American Indian/Alaska Natives (15.1%), non-Hispanic Blacks (12.7%), and Latinos (12.1%), as compared to non-Hispanic Whites (7.4%); and it is noteworthy that among Latinos the disease rate is highest among Mexicans (13.8%) [54]. Despite the elevated rates of diabetes among Latinos, there are few studies that have investigated the longitudinal association of certain risk factors on the development of pre-diabetes or diabetes in this population [55–57]. The aim of this study was to determine the association between known risk factors and the development of pre-diabetes or diabetes in a cohort of Mexican health workers. We examined clinical and questionnaire data obtained as part of a longitudinal study, to determine whether participants who acquired or maintained specific risk factors would have a higher risk of pre-diabetes or diabetes, compared to those who did not.

## Materials and methods

### Study design and population

Approvals for both the Mexican Health Worker Cohort Study (MHWCS) and this specific investigation were obtained from the Internal Review Boards of the Mexican Social Security Institute (IMSS) (2005-785-012) and the University of California, Los Angeles (UCLA) (IRB#17–001028). Data were collected from participants in the MHWCS, a longitudinal study of health workers and their immediate family members from two large institutes in Cuernavaca, Mexico, the Mexican Institute of Social Security (IMSS) and the National Institute of Public Health. The purpose of the MHWCS is to prospectively evaluate the incidence of specific risk factors for chronic diseases in an urban area of Central Mexico. During the period from 2004 to 2006 (Wave 1; W1) a total of 5,869 participants from Cuernavaca enrolled in the MHWCS, and 1,571 of these participants were followed up during 2011 to 2013 (Wave 2; W2). The mean follow-up time for the participants was 7.0 years, with a standard deviation (SD) of 1.1 years. Written informed consent was obtained from all participants prior to initiating any data collection activities, which included completing self-reported questionnaires, physical examinations, and routine laboratory tests. Body weight was determined using a calibrated electronic scale (model BC-533; Tanita) with participants wearing minimal clothing and no shoes. Height was measured using a conventional stadiometer (Seca), with barefoot participants standing with their shoulders in a normal position. Measurements were taken with the tape in a horizontal plane perpendicular to the vertical scale, touching the top of the head at the moment of inspiration. Waist circumference was measured at the highest point of the iliac crest to the nearest 0.1 cm. Additional details regarding the methods and design of the MHWCS are available elsewhere [58–60].

Of the 1,571 W1 participants who were followed up during W2, a total of 1,418 were over the age of 20, and 184 individuals were excluded due to incomplete laboratory test information in W1. Other participants were excluded for being pregnant (n = 15), or having incomplete information on body mass index (BMI), waist circumference, HDL cholesterol, physical activity, alcohol consumption, or average daily calories consumed (n = 45). Our final sample consisted of 1,174 participants over the age of 20 years with complete questionnaire and laboratory data from W1 and W2. Although only 20% of the 5,863 participants from W1 were followed up in W2, there were no significant differences between the two groups in terms of sex (p-value = 0.71), BMI (p = 0.96), blood pressure (p = 0.22), waist circumference (p = 0.49), prevalence of metabolic syndrome (p = 0.99), elevated HDL cholesterol (p = 0.99), triglycerides (p = 0.58), or uric acid (p = 0.89). There were statistical differences between the two groups by age (p <0.001), education status (p = 0.02), and elevated ALT levels (p <0.001).

### Independent variables

#### Body Mass Index (BMI)

The weight classification of study participants was determined based on the following recommendations from the National Heart, Lung, and Blood Institute: normal weight (18.5–24.9 kg/m2), overweight (25.0–29.9 kg/m2) and obese (≥30 kg/m2) [61].

#### Metabolic syndrome (MS)

We used a modified version of the 2009 Criteria for Clinical Diagnosis of the Metabolic Syndrome reported by Alberti et al., as having three or more of the following factors: (1) waist circumference ≥80 cm for females and ≥90 cm for males; (2) triglycerides ≥150 mg/dL; (3) HDL-C <50 mg/dL for females and <40 mg/dL for males; and (4) systolic blood pressure ≥130mmHg, diastolic blood pressure ≥85mmHg, blood pressure (BP), or currently taking blood pressure medications [62]. Since level of fasting plasma blood glucose was used to ascertain diabetes status, the dependent variable in the study, we did not include this component in our definition of MS, which would otherwise additionally include a fasting plasma glucose ≥100 mg/dL.

#### Alanine aminotransferase (ALT)

Elevated ALT was defined as >40 U/L for males and females [63,64]. To determine ALT level, the catalytic concentration from the rate of decrease in nicotinamide adenine dinucleotide was measured at 340 nm using a coupled reaction of lactate dehydrogenase (LDH) [59].

#### Alcohol consumption

Study participants were identified as either non-/moderate drinkers or heavy/binge drinkers based on the following definitions from the Centers for Disease Control and Prevention. Moderate drinking is ≤ 1 drink per day for females and ≤ 2 drinks per day for males. Heavy drinking for females is 2–4 drinks per day and 3–4 drinks per day for males. Binge drinking is ≥ 5 drinks on at least one occasion for both females and males [65].

#### Leisure physical activity

Physical activity level was assessed using a self-administered questionnaire that was previously validated in Spanish. Participants reported number of minutes engaged in different recreational physical activities during a typical week in the last year [66]. Participants were categorized as either achieving 30 minutes of recreational physical activity per day or not. The average number of hours/week spent in each of the following activities was assessed: walking, running, cycling, aerobics, dancing, and swimming as well as playing football, volleyball, basketball, tennis, fronton, baseball, softball, and squash, among others [66].

#### Uric acid

An elevated level of serum uric acid (hyperuricemia) was defined as ≥5.7 mg/dL in females and ≥7.0 mg/dL in males [67,68]. Serum uric acid levels were determined with the enzymatic colorimetric method using the SYNCHRON CX system [69].

#### Calorie consumption

A validated semi-quantitative food frequency questionnaire was used to collect diet data based on the consumption patterns of 116 food items during the previous year [58]. Energy and nutrient intake were calculated by multiplying the consumption frequency of each reported food type by the nutrient content obtained from a comprehensive food database [70,71]. Average daily calories consumed were recorded in units of kilocalories and reported as a continuous variable. Extreme values of caloric intake (<600 and >7000 Kcal/day) were excluded from the analysis as being unrealistic outliers.

### Dependent variable

Level of fasting plasma blood glucose (FPG) was used to ascertain diabetes status, the dependent variable in the study. Participants were categorized according to their FPG as normal, pre-diabetic, or diabetic, based on guidelines from the American Diabetes Association [72]. Normal was defined as a FPG <100 mg/dL, pre-diabetes was defined as ≥100 mg/dL and <126 mg/dL, and diabetes was defined as ≥126 mg/dL. Additionally, participants were categorized as having diabetes if they (1) had a medical history of diabetes (not including gestational diabetes) or (2) were currently taking diabetes medication. FPG levels were determined from blood samples that were obtained from participants after fasting for 12 hours. Blood samples were centrifuged and frozen at -20 °C. and then processed at IMSS. All biomedical assays were processed using a Selectra XL instrument (Randox), according to the International Federation of Clinical Chemistry and Laboratory Medicine guidelines [73].

### Statistical analysis

All statistical analyses were performed using Stata 14 (StataCorp LP, College Station, Texas, USA). The unadjusted cross-sectional associations of each risk factor at W1 with normal, pre-diabetic, and diabetic FPG levels were examined using χ2 tests. Adjusted odds ratios (OR) and 95% confidence intervals (CI) for cross-sectional associations at W1 were calculated using multivariable binary logistic regression models, with separate models for pre-diabetes versus normal and diabetes versus normal. Models were adjusted for sex, age, and education, then additionally for BMI, alcohol consumption, ALT, physical activity, and daily calorie intake.

To evaluate the longitudinal association between risk factors and progression to pre-diabetes or diabetes at W2, the sample was restricted to individuals with normal blood glucose levels at W1 and multinomial logistic regression models were used to calculate adjusted relative risk ratios (RRR) and 95% CI for diabetes-related outcomes (normal, pre-diabetes, diabetes) at W2. BMI status over time was categorized into five groups: (1) remained normal: normal in W1 to normal in W2; (2) weight loss: obese in W1 to overweight in W2, obese in W1 to normal in W2, or overweight in W1 to normal in W2; (3) remained overweight: overweight in W1 to overweight in W2; (4) remained obese: obese in W1 to obese in W2; (5) weight gain that resulted in a BMI category change: normal in W1 to overweight in W2, normal in W1 to obese in W2, or overweight in W1 to obese in W2. MS status over time was categorized into four groups: (1) never had MS: MS absent in W1 to MS absent in W2; (2) no longer have MS: MS present in W1 to MS absent in W2; (3) developed MS: MS absent in W1 to MS present in W2; and (4) remained with MS: MS present in W1 to MS present in W2. Status over time for waist circumference, HDL-C, triglycerides, blood pressure, ALT and uric acid were also classified into four groups: (1) never abnormal/elevated: normal in W1 to normal in W2; (2) no longer abnormal/elevated: abnormal/elevated in W1 to normal in W2; (3) became abnormal/elevated: normal in W1 to abnormal/elevated in W2; and (4) remained abnormal/elevated: abnormal/elevated in W1 to abnormal/elevated in W2. Models were fit: (1) adjusted for sex, age, and education at W1, and (2) additionally adjusted for BMI, alcohol consumption, ALT, physical activity, and daily calorie intake at W1. When specific variables were included as predictors they were not adjusted for in the analyses. For participants who were missing education status, alcohol consumption, leisure physical activity, and daily calorie consumption at W1 (n = 127), the W2 measure was used. For the 19 remaining participants with missing education status, education was imputed using an ordinal logistic regression model (education regressed on sex, age and marital status) estimated using data from individuals with complete data. There were no missing data for the other independent variables.

The average predicted probabilities of being classified as having normal blood glucose, pre-diabetes or diabetes given specific characteristics (sex and number of metabolic syndrome components for W1 cross-sectional analysis; each risk factor of interest for longitudinal analyses) were obtained using the Stata margins command; 95% CIs were obtained using the delta method. These results were adjusted by the full set of control variables. For all analyses, a two-sided P value <0.05 was considered statistically significant.

## Results

Table 1 presents the socio-demographic characteristics of the study participants and compares the prevalence of each risk factor for normal, pre-diabetic and diabetic FPG, unadjusted for any other factors. The proportion of males with pre-diabetes was greater than that of females (25% vs. 14%, respectively). Pre-diabetes and diabetes increased with age, with the highest prevalence observed among those aged 45–59 years and over the age of 60. Having a high school education or less was associated with a higher prevalence of pre-diabetes and diabetes. The prevalence of pre-diabetes and diabetes also increased with higher BMI. Among the obese participants, 25% had pre-diabetes and 19% had diabetes. Participants with metabolic syndrome (MS) had a slightly lower, but still high prevalence of pre-diabetes (22%) and diabetes (15%). Additionally, the prevalence of pre-diabetes and diabetes increased as the number of MS components increased. Having elevated ALT levels or an abnormal waist circumference was also associated with a higher prevalence of pre-diabetes and diabetes. A significant difference was observed in the prevalence of all the aforementioned risk factors, between participants with normal glucose levels and those with pre-diabetes or diabetes.

**Table 1:** Characteristics of total sample and participants with normal^a^, pre-diabetic^b^, and diabetic^c^ blood glucose levels at Wave 1. (N = 1,174): n (%).

	Wave 1						
	Total N = 1,174	Normal^a^ n = 867 (74)	Pre-diabetic^b^ n = 191 (16)	Diabetic^c^ n = 116 (10)	P-values^d^		
**Sex**					A	B	C
Female	895 (76)	683 (76)	122 (14)	90 (10)	<0.001	0.769	0.012
Male	279 (24)	184 (66)	69 (25)	26 (9)			
**Age**							
≤ 44 years	455 (39)	392 (86)	47 (10)	16 (4)	<0.001	<0.001	<0.001
45–59 years	484 (41)	323 (67)	107 (21)	54 (12)			
≥ 60 years	235 (20)	152 (64)	37 (16)	46 (20)			
**Education status**							
≤ High school	428 (36)	278 (65)	80 (19)	70 (16)	0.009	<0.001	0.002
> High school	746 (64)	589 (79)	111 (15)	46 (6)			
**BMI**^e^							
Normal	436 (37)	372 (85)	41 (10)	23 (5)	<0.001	<0.001	0.412
Overweight	499 (43)	361 (72)	90 (18)	48 (10)			
Obese	239 (20)	134 (56)	60 (25)	45 (19)			
**Metabolic syndrome (MS)**^f^							
No	607 (52)	511 (84)	65 (11)	31 (5)	<0.001	<0.001	0.181
Yes	567 (48)	356 (63)	126 (22)	85 (15)			
**Number of MS components**							
None or one	219 (19)	193 (88)	18 (8)	8 (4)	<0.001	<0.001	0.086
Two	388 (33)	318 (82)	47 (12)	23 (6)			
Three	403 (34)	268 (67)	88 (22)	47 (12)			
Four	164 (14)	88 (54)	38 (23)	38 (23)			
**Elevated ALT**^g^							
No	958 (82)	739 (77)	135 (14)	84 (9)	<0.001	<0.001	0.745
Yes	216 (18)	128 (59)	56 (26)	32 (15)			
**Alcohol**^h^							
Non-drinker/ Moderate	993 (85)	741 (75)	152 (15)	100 (10)	0.042	0.831	0.142
Heavy/ Binge	181 (15)	126 (70)	39 (21)	16 (9)			
**Leisure physical activity level**							
≤30 minutes/day	827 (70)	603 (73)	140 (17)	84 (10)	0.305	0.528	0.866
>30 minutes/day	347 (30)	264 (76)	51 (15)	32 (9)			
**Waist Circumference**^i^							
Normal	227 (19)	203 (89)	16 (7)	8 (4)	<0.001	<0.001	0.639
Elevated	947 (81)	664 (70)	175 (19)	108 (11)			
**Uric Acid**^j^							
Normal	922 (79)	700 (76)	133 (14)	89 (10)	0.001	0.308	0.178
Elevated	252 (21)	167 (66)	58 (23)	27 (11)			
**Average daily calories**							
Mean (SD)	2,103 (867)	2,115 (882)	2,085 (857)	2,035 (762)	0.666	0.359	0.614

^a^ Normal blood glucose was defined as a fasting plasma glucose less than 100 mg/dL.

^b^ Pre-diabetes was defined as a fasting plasma glucose level between 100 and 125 mg/ dL.

^c^ Diabetes was defined as a self-reported medical history of diabetes, current use of diabetes medications, and/or a fasting plasma glucose level ≥ 126 mg/ dL.

^d^ P-value was computed using the Chi-square test. (A) p-value compares normal and pre-diabetic individuals, (B) p-value compares normal and diabetic individuals, (C) p-value compares pre-diabetic and diabetic individuals.

^e^ Body mass index (BMI) was defined as normal <25 kg/m^2^, overweight between 25- <30 kg/m^2^, or obese ≥ 30 kg/m^2^.

^f^ Metabolic Syndrome was defined based on the 2009 Criteria for Clinical Diagnosis of the Metabolic Syndrome reported by Alberti et al [62], but we excluded glucose as one of the components.

^g^ Elevated alanine aminotransferase (ALT) was defined as ALT > 40 IU/L for males and females.

^h^ Alcohol intake was defined as follows: nondrinkers- no history of drinking, moderate- ≤1 drink a day for females and ≤ 2 drinks per day for males, heavy- 2–4 drinks per day for females and 3–4 for males, binge- ≥5 drinks per day for females and males.

^i^ Elevated waist circumference is defined as ≥ 80 cm for females and ≥ 90 cm for males [62].

^j^ Uric acid levels ≥ 7.0 mg/dL for males and ≥ 5.7 mg/dL for females are considered normal.

A cross-sectional analysis of Wave 1 data comparing the risk factors among those with pre-diabetes and diabetes to those with normal blood glucose adjusting for other factors is shown in Table 2. These results are adjusted by sex, age, education, BMI, alcohol consumption, elevated ALT, physical activity, and calorie consumption. Males have a greater than two-fold odds of pre-diabetes (OR 2.3, 95%, CI 1.6–3.5), compared to females. Participants between the ages of 45–59 years have approximately three-fold higher odds of pre-diabetes and diabetes (OR 2.7, 95% CI 1.8–4.0 and OR 3.6, 95% CI 1.9–6.6, respectively), compared to persons ≤44 years of age. Participants aged 60 years or older have a two-fold greater odds of pre-diabetes (OR 2.0, 95% CI 1.2–3.5), and 5.6-fold higher odds of diabetes (95% CI 2.8–11.5), than persons ≤44 years of age. Participants with an education beyond high school have 50% lower odds (95% CI 0.3–0.9) of diabetes than those with a high school education or less. Overweight and obese participants have 1.7 (95% CI 1.1–2.6) and 2.9 (95% CI 1.8–4.7) higher odds, respectively, of pre-diabetes, while obese participants have 3.6-fold higher odds (95% CI 2.0–6.5) of diabetes, compared to normal weight individuals. The presence of metabolic syndrome (without considering glucose) is associated with a 1.8 (95% CI 1.3–2.6) times greater odds of pre-diabetes and two-fold greater odds of diabetes (OR 2.0, 95% CI 1.2–3.2). Having elevated triglycerides is also significantly associated with pre-diabetes (OR 1.7, 95% CI 1.2–2.4) and diabetes (OR 2.0, 95% CI 1.3–3.1). Participants with elevated blood pressure have a nearly two-fold higher odds of diabetes (OR 1.9, 95% CI 1.2–2.9) than those with normal blood pressure. Participants with three MS components have an almost two times greater odds (OR 1.9, 95% CI 1.0–3.4) of pre-diabetes, compared to participants with none or only one MS component. Participants with four MS components have two-fold higher odds (95% CI 1.0–4.0) of pre-diabetes and 2.7-fold higher odds (95% CI 1.1–6.6) of diabetes, compared to participants with none or only one MS component. Elevated ALT is associated with approximately twice the odds of pre-diabetes (OR 1.7, 95% CI 1.1–2.5), and diabetes (OR 2.1, 95% CI 1.3–3.4), compared to those with non-elevated ALT.

**Table 2:** Cross-sectional analysis comparing pre-diabetics^a^ (n = 191) and diabetics^b^ (n = 116) to those with normal^c^ blood glucose levels (n = 867) in Wave 1 (W1). (N = 1,174).

	n	Risk factors for pre-diabetes^a^		Risk factors for diabetes^b^	
		Model 1^d^	Model 2^e^	Model 1^d^	Model 2^e^
		OR (95% CI)	OR (95% CI)	OR (95% CI)	OR (95% CI)
**Sex**					
Female	895	1.00	1.00	1.00	1.00
Male	279	2.5 (1.8–3.6)**	2.3 (1.6–3.5)**	1.4 (0.9–2.3)	1.6 (0.9–2.7)
**Age**					
≤ 44	455	1.00	1.00	1.00	1.00
45–59	484	2.9 (2.0–4.3)**	2.7 (1.8–4.0)**	3.6 (2.0–6.5)**	3.6 (1.9–6.6)**
≥ 60	235	1.9 (1.2–3.3)*	2.0 (1.2–3.5)*	5.0(2.6–9.7)**	5.6 (2.8–11.5)**
**Education status**					
≤ High school	428	1.00	1.00	1.00	1.00
> High school	746	0.7 (0.5–1.0)	0.8 (0.5–1.1)	0.5 (0.3 0.8)**	0.5 (0.3–0.9)*
**BMI**^f^					
Normal	436	1.00	1.00	1.00	1.00
Overweight	499	1.9 (1.2–2.8)**	1.7 (1.1–2.6)*	1.7 (0.9–2.9)	1.6 (0.9–2.8)
Obese	239	3.3 (2.1–5.3)**	2.9 (1.8–4.7)**	4.2 (2.4–7.4)**	3.6 (2.0–6.5)**
**Metabolic syndrome (MS)**^g^					
No	607	1.00	1.00	1.00	1.00
Yes	567	2.4 (1.7–3.4)**	1.8 (1.3–2.6)**	2.9 (1.8–4.5)**	2.0 (1.2–3.2)**
**Waist circumference**^h^					
Normal	227	1.00	1.00	1.00	1.00
Elevated	947	3.1 (1.8–5.5)**	1.8 (0.99–3.3)	2.9 (1.3–6.1)**	1.3 (0.6–3.0)
**HDL cholesterol**^i^					
Normal	184	1.00	1.00	1.00	1.00
Low	990	0.8 (0.5–1.2)	0.7 (0.4–1.1)	1.0 (0.6–1.8)	0.8 (0.5–1.5)
**Triglycerides**^j^					
Normal	654	1.00	1.00	1.00	1.00
Elevated	520	2.0 (1.4–2.7)**	1.7 (1.2–2.4)**	2.4 (1.6–3.6)**	2.0 (1.3–3.1)**
**Blood pressure**^k^					
Normal	807	1.00	1.00	1.00	1.00
Elevated	367	1.8 (1.3–2.5)**	1.4 (0.96–2.0)	2.5 (1.6–3.7)**	1.9 (1.2–2.9)**
**Number of MS components**					
None or one	219	1.00	1.00	1.00	1.00
Two	388	1.5 (0.8–2.7)	1.1 (0.6–2.0)	1.4 (0.6–3.2)	0.8 (0.4–2.0)
Three	403	3.0 (1.7–2.7)**	1.9 (1.0–3.4)*	2.8 (1.3–6.2)*	1.4 (0.6–3.2)
Four	164	3.5 (1.8–6.6)**	2.0 (1.0–4.0)*	5.7 (2.5–13.1)**	2.7 (1.1–6.6)*
**Elevated ALT**^l^					
No	958	1.00	1.00	1.00	1.00
Yes	216	2.2 (1.5–3.3)**	1.7 (1.1–2.5)*	2.5 (1.6–4.1)**	2.1 (1.3–3.4)**
**Uric Acid**^m^					
Normal	922	1.00	1.00	1.00	1.00
Elevated	252	1.5 (1.0–2.2)*	1.1 (0.8–1.7)	1.0 (0.6–1.6)	0.7 (0.4–1.2)

^a^ Pre-diabetes was defined as a fasting plasma glucose level between 100 and 125 mg/ dL.

^b^ Diabetes was defined as a self-reported medical history of diabetes, current use of diabetes medications, and/or a fasting plasma glucose level ≥ 126 mg/ dL.

^c^ Normal blood glucose was defined as a fasting plasma glucose <100 mg/dL.

^d^ Model 1: Adjusted for sex, age, education.

^e^ Model 2: Adjusted for sex, age, education, BMI, alcohol consumption, ALT, physical activity, daily calories.

^f^ Body mass index (BMI) was defined as normal <25 kg/m2, overweight between 25- <30 kg/m2, or obese ≥ 30 kg/m2.

^g^ Metabolic Syndrome was defined based on the 2009 Criteria for Clinical Diagnosis of the Metabolic Syndrome reported by Alberti et al [62], but we excluded glucose as one of the components.

^h^ Abnormal waist circumference is defined as ≥ 80 cm for females and ≥ 90 cm for males [62].

^i^ Low HDL-Cholesterol is defined as <50 mg/dL for females and <40 mg/dL for males.

^j^ Elevated triglycerides is defined as ≥150 mg/dL for females and males.

^k^ Elevated blood pressure is defined as ≥130/85 mmHg, or currently taking blood pressure medications.

^l^ Elevated alanine aminotransferase (ALT) was defined as ALT > 40 IU/L for males and females.

^m^ Uric acid levels ≥ 7.0 mg/dL for males and ≥ 5.7 mg/dL for females are considered normal.

** P < 0.01 for test of null hypothesis that the odds ratio is equal to the odds ratio in the reference category.

* P < 0.05 for test of null hypothesis that the odds ratio is equal to the odds ratio in the reference category.

**Fig 1:**
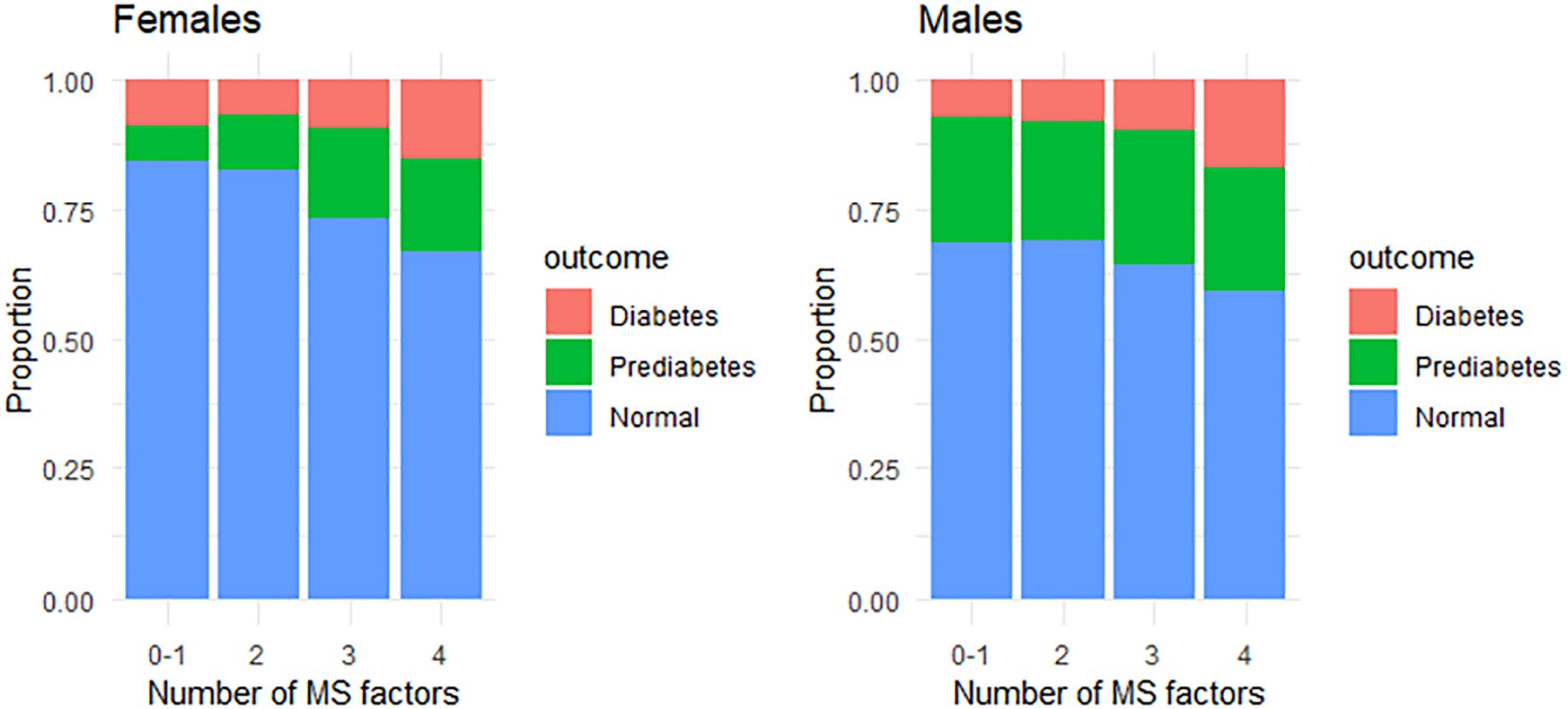
Average predicted probabilities of having normal blood sugar, pre-diabetes, or diabetes in Wave 1, by number of metabolic syndrome (MS) components and sex. (n = 1,174). Results correspond to Model 2 in Table 2.

To aid interpretation, the fully adjusted Model 2 results were converted to average predicted probabilities of having normal blood sugar, pre-diabetes, or diabetes in Wave 1, in relation to the number of MS components, for males and females (Fig 1). The average predicted probability of having diabetes was comparable for males and females, regardless of the number of MS components. However, the average predicted probability of having pre-diabetes was substantially higher among males across all MS component counts. Accordingly, males also have a substantially lower average predicted probability of having normal blood sugar levels than females in the four categories. Among females, the average predicted probability of having normal blood glucose was 0.84 (95% CI 0.77–0.92) for the lowest and 0.67 (95% CI 0.59–0.75) for the highest number of MS components; among males, these values were 0.68 (95% 0.55–0.81) and 0.59 (95% CI 0.46–0.72), respectively.

**Fig 2:**
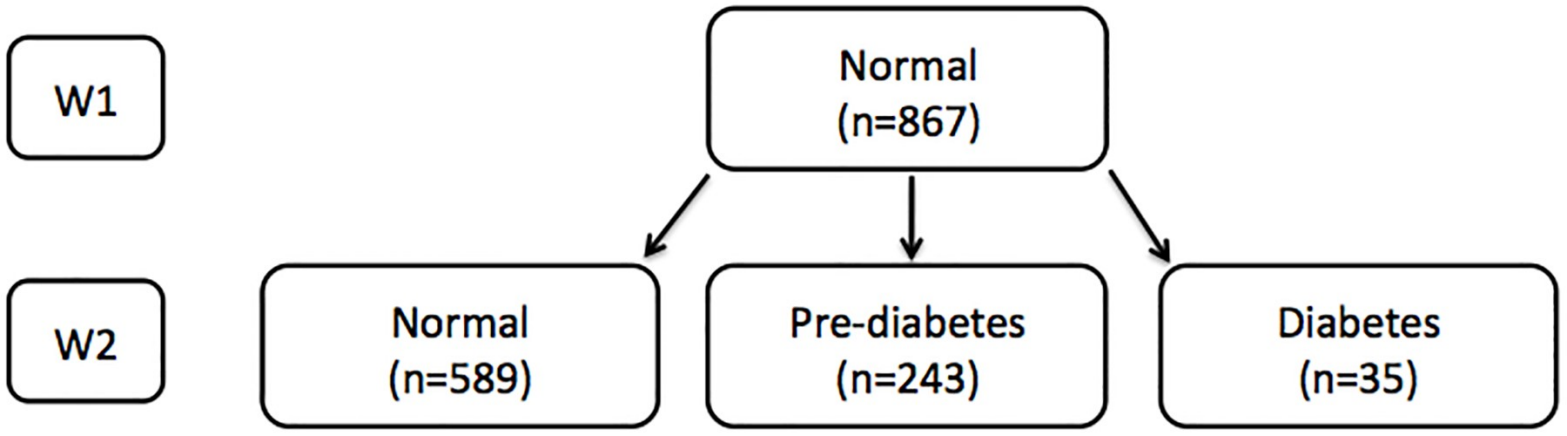
Study sample analyzed to determine the relative risk of developing pre-diabetes or diabetes at follow-up among those with normal blood glucose levels at baseline.

Fig 2 describes the subsample of study participants who had a normal glucose levels at W1, and either remained normal, or developed pre-diabetes or diabetes at W2. The same two analysis models that generated the results presented in Table 2 were used to determine the findings presented in Table 3, but here we focus on the results of Model 2, which are similar to those of Model 1.

**Table 3:** Change in risk factors from Wave 1 (W1) to Wave 2 (W2) and relative risk of developing pre-diabetes^a^ (n = 243) or diabetes^b^ (n = 35) in W2 among those with normal^c^ blood glucose levels (N = 867) in Wave 1.

	n	Relative risk ratios (RRR) for those who developed pre-diabetes in W2		Relative risk ratios (RRR) for those who developed diabetes in W2	
		Model 1^d^	Model 2^e^	Model 1^d^	Model 2^e^
		RRR (95% CI)	RRR (95% CI)	RRR (95% CI)	RRR (95% CI)
**Sex**					
Female	683	1.00	1.00	1.00	1.00
Male	184	1.8 (1.3–2.6)**	2.0 (1.3–3.0)**	1.7 (0.7–4.4)	1.7 (0.6–4.9)
**Age**					
≤ 44	224	1.00	1.00	1.00	1.00
45–59	369	1.7 (1.1–2.7)*	1.5 (0.9–2.3)	0.8 (0.1–4.4)	1.6 (0.4–6.2)
≥ 60	274	1.6 (0.8–3.2)	1.4 (0.7–2.8)	0.6 (0.1–4.3)	0.9 (0.1–6.2)
**Education status**					
≤ High school	278	1.00	1.00	1.00	1.00
> High school	589	1.1 (0.5–2.4)	0.9 (0.4–2.2)	0.5 (0.1–2.6)	0.4 (0.1–2.7)
**BMI**					
Remained normal	270	1.00	1.00	1.00	1.00
Weight loss	57	0.9 (0.4–1.9)	0.8 (0.3–1.8)	4.8 (1.1–20.3)*	4.2 (0.97–17.9)
Remained Overweight	268	2.0 (1.3–3.0)**	1.9 (1.3–2.9)**	4.3 (1.4–13.3)*	4.2 (1.3–13.1)*
Remained Obese	114	3.8(2.3–6.3)**	3.4 (2.0–5.8)**	7.1 (2.1–24.4)**	7.6 (2.2–26.3)**
Weight gain	158	2.4 (1.5–3.7)**	2.4 (1.5–3.7)**	1.7 (0.4–7.7)	1.6 (0.4–7.4)
**Metabolic syndrome (MS)**					
Never had MS	342	1.00	1.00	1.00	1.00
No longer have MS	93	2.0 (1.2–3.4)*	1.5 (0.9–2.7)	2.1 (0.5–8.7)	1.3 (0.3–5.7)
Developed MS	169	2.1 (1.4–3.2)**	1.9 (1.2–2.9)**	3.7 (1.3–10.8)*	3.0 (1.0–9.0)*
Remained with MS	263	3.0 (2.0–4.4)**	2.2 (1.5–3.4)**	4.9 (1.8–13.1)**	2.7 (0.9–7.5)
**Waist Circumference**					
Never elevated	75	1.00	1.00	1.00	1.00
No longer elevated	33	2.1 (0.7–6.7)	1.9 (0.6–6.2)	n/a^f^	n/a^f^
Became elevated	128	3.7 (1.6–8.6)**	3.4 (1.4–7.9)**	n/a^f^	n/a^f^
Remained elevated	631	4.6 (2.1–9.9)**	2.8 (1.2–6.5)*	n/a^f^	n/a^f^
**HDL cholesterol**					
Never low	93	1.00	1.00	1.00	1.00
No longer low	258	1.9 (0.99–3.5)	1.8 (0.9–3.4)	0.5 (0.1–1.8)	0.5 (0.1–1.9)
Became low	37	2.2 (0.9–5.4)	1.8 (0.7–4.5)	0.7 (0.1–6.1)	0.6 (0.1–6.1)
Remained low	479	2.9 (1.6–5.2)**	2.5 (1.3–4.6)**	1.3 (0.5–3.6)	1.0 (0.4–3.1)
**Triglycerides**					
Never elevated	343	1.00	1.00	1.00	1.00
No longer elevated	71	1.9 (1.0–3.3)*	1.6 (0.9–3.0)	3.2 (0.9–11.6)	2.5 (0.6–9.5)
Became elevated	191	1.7 (1.1–2.6)*	1.6 (1.1–2.5)*	2.6 (0.9–7.3)	2.3 (0.8–6.7)
Remained elevated	262	2.4 (1.7–3.6)**	2.0 (1.4–3.0)**	3.6 (1.4–9.1)**	3.2 (1.2–8.1)*
**Blood pressure**					
Never elevated	461	1.00	1.00	1.00	1.00
No longer elevated	42	1.3 (0.6–2.6)	1.1 (0.5–2.4)	1.1 (0.1–8.7)	0.7 (0.1–5.8)
Became elevated	185	1.9 (1.3–2.9)**	1.6 (1.1–2.5)*	3.9 (1.6–9.6)**	3.3 (1.3–8.2)*
Remained elevated	179	1.8 (1.2–2.8)**	1.3 (0.8–2.1)	3.6 (1.4–9.4)**	1.8 (0.6–5.2)
**ALT**					
Never elevated	687	1.00	1.00	1.00	1.00
No longer elevated	71	1.3 (0.7–2.2)	1.1 (0.6–1.9)	1.9 (0.6–6.0)	1.4 (0.4–4.6)
Became elevated	52	2.6 (1.4–4.8)**	2.2 (1.2–4.2)*	7.6 (2.6–21.8)**	6.1 (2.1–17.8)**
Remained elevated	57	3.1 (1.7–5.5)**	2.5 (1.3–4.5)**	4.7 (1.5–14.0)**	3.4 (1.1–10.6)*
**Uric Acid**					
Never elevated	580	1.00	1.00	1.00	1.00
No longer elevated	57	0.8 (0.4–1.5)	0.6 (0.3–1.2)	4.8 (1.5–15.2)*	3.0 (0.9–9.9)
Became elevated	120	1.4 (0.9–2.2)	1.3 (0.8–2.0)	4.5 (1.7–12.0)**	4.7 (1.7–12.6)*
Remained elevated	110	1.5 (0.96–2.4)	1.1 (0.6–1.7)	8.0 (3.2–20.2)**	5.1 (2.0–13.2)**

^a^ Pre-diabetes was defined as a fasting plasma glucose level between 100 and 125 mg/dL.

^b^ Diabetes was defined as a self-reported medical history of diabetes, current use of diabetes medications, and/or a fasting plasma glucose level ≥ 126 mg/ dL.

^c^ Normal blood glucose was defined as a fasting plasma glucose <100 mg/dL.

^d^ Model 1: Adjusted for sex, age, education.

^e^ Model 2: Adjusted for sex, age, education, BMI, alcohol consumption, ALT, physical activity, calories.

** P < 0.01 for test of null hypothesis that the odds ratio is equal to the odds ratio in the reference category.

* P < 0.05 for test of null hypothesis that the odds ratio is equal to the odds ratio in the reference category.

^f^ Too few participants were categorized as having referenced outcomes

Table 3 reports the longitudinal analysis of the association between changes in risk factors from W1 to W2 and the relative risk of developing pre-diabetes or diabetes in a subsample of participants with normal blood glucose levels in W1. As observed in the cross-sectional analysis, males have a two-fold higher relative risk of pre-diabetes (RRR 2.0, 95% CI 1.3–3.0) than females. Participants who acquired or continued to have certain risk factors over time are significantly more likely to have transitioned from normal blood glucose levels in W1 to pre-diabetes or diabetes in W2, than those who did not acquire or maintain these risk factors. Individuals who remained overweight have an almost two-fold higher relative risk of developing pre-diabetes (RRR 1.9, 95% CI 1.3–2.9) and an over four-fold greater relative risk of diabetes (RRR 4.2, 95% CI 1.3–13.1). Participants who maintained an obese BMI have an over three-fold higher relative risk of developing pre-diabetes (RRR 3.4, 95% CI 2.0–5.8) and an over seven-fold greater relative risk of diabetes (RRR 7.6, 95% CI 2.2–26.3). Maintaining the following factors also results in an increased risk of pre-diabetes: metabolic syndrome (RRR 2.2, 95% CI 1.5–3.4); elevated waist circumference (RRR 2.8, 95% CI 1.2–6.5); low HDL cholesterol (RRR 2.5, 95% CI 1.3–4.6); elevated triglycerides (RRR 2.0, 95% CI 1.4–3.0); as well as elevated ALT (RRR 2.5, 95% CI 1.3–4.5). A similar trend is observed for individuals who maintained the following factors over time, and are also significantly more likely to have transitioned from normal blood glucose levels in W1 to diabetes in W2: elevated triglycerides (RRR 3.2, 95% CI 1.2–8.1); elevated ALT (RRR 3.4, 95% CI 1.1–10.6); and elevated uric acid (RRR 5.1 95% CI 2.0–13.2). Participants who acquired specific risk factors over time are significantly more likely to have transitioned from normal blood glucose levels in W1 to pre-diabetes in W2. This included the following risk factors: weight gain (RRR 2.4, 95% CI 1.5–3.7); metabolic syndrome (RRR 1.9, 95% CI 1.2–2.9); elevated waist circumference (RRR 3.4, 95% CI 1.4–7.9); elevated triglycerides (RRR 1.6, 95% CI 1.1–2.5); elevated blood pressure (RRR 1.6, 95% CI 1.1–2.5); and elevated ALT (RRR 2.2, 95% CI 1.2–4.2). The same trend also occurred among individuals who developed certain factors over time and are also significantly more likely to have transitioned from normal blood glucose levels in W1 to diabetes in W2. This was observed for the following risk factors: metabolic syndrome (RRR 3.0, 95% CI 1.0–9.0); elevated blood pressure (RRR 3.3, 95% CI 1.3–8.2); elevated ALT (RRR 6.1, 95% CI 2.1–17.8); and elevated uric acid (RRR 4.7, 95% CI 1.7–12.6).

**Fig 3:**
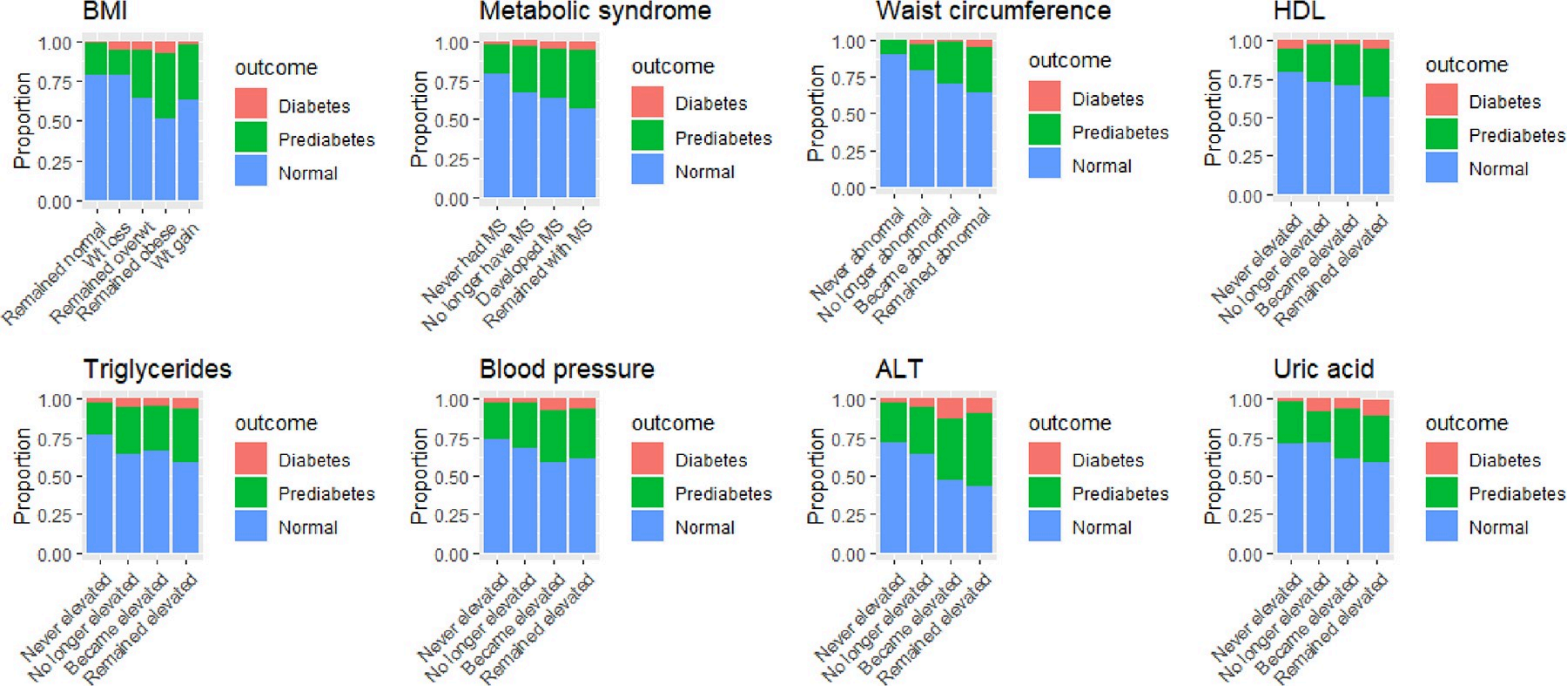
Average predicted probabilities of remaining with a normal blood glucose, developing pre-diabetes, or developing diabetes in Wave 2 among participants with normal blood glucose levels (n = 867) in Wave 1. Results based on Model 2 in Table 3.

To facilitate interpretation, the average predicted probabilities of maintaining normal blood sugar levels, developing pre-diabetes, or developing diabetes in Wave 2, among participants who had normal blood glucose in Wave 1, are shown in Fig 3. These results correspond to the fully adjusted Model 2 presented in Table 3. The lowest probabilities of maintaining a normal blood glucose level, and thus the highest probabilities of progressing to pre-diabetes or diabetes, were found among individuals who remained obese (0.52, 95% CI 0.42–0.61), remained with MS (0.57, 95% CI 0.51–0.63), remained with elevated triglycerides (0.59, 95% CI 0.53–0.65), acquired high blood pressure (0.59, 95% CI 0.52–0.66), had ALT that remained elevated (0.44, 95% CI 0.31–0.57) or became elevated (0.47, 95% 0.33–0.61), or maintained elevated uric acid levels (0.58, 95% CI 0.49–0.67).

## Discussion

This study examined the changes over time in risk factors for diabetes among a cohort of Mexican health workers who initially had either normal fasting plasma glucose (FPG) or pre-diabetes. As anticipated, after controlling for demographic variables, BMI, lifestyle habits (alcohol, diet, physical activity), and ALT, our results indicate that several factors were associated with a greater risk of developing pre-diabetes and diabetes in our sample. These factors include having an elevated BMI, triglycerides, or blood pressure, and the presence of metabolic syndrome (MS). With the exception of elevated blood pressure and elevated BMI, these trends were maintained over time among participants who were first observed as having normal FPG. These findings are consistent with other studies that examined the association between hypertension and diabetes in Latino populations [14]. Another study found that increased BMI and blood pressure were present after the onset of diabetes in a group of adults in Southern California who were followed for 8 years [15]. Other researchers report that individuals with MS have a five times greater risk of developing diabetes [74]. Metabolic syndrome has consistently been shown to be a strong predictor for the onset of diabetes, making it an important target for further research on this topic. Neither waist circumference nor overweight BMI were associated with diabetes status in the more adjusted model of the cross-sectional analysis, which could be due to some patients losing weight after receiving their diagnosis. In the longitudinal analysis, however, we observed a strong association with remaining overweight or obese over time and the development of diabetes. A similar trend was seen for triglycerides and uric acid, suggesting a prolonged exposure is needed to affect development of diabetes.

Additionally, we found that elevated uric acid is associated with greater risk of developing diabetes, although this association was not observed among those who progressed to pre-diabetes. Further comparisons reveal that participants who either remained with or developed elevated levels of serum uric acid have a 4.4 (95% CI 1.7–11.7) and 3.1 (95% CI 1.1–8.6) increased risk, respectively, of developing diabetes, compared to pre-diabetes, during the study period. Increased uric acid has been identified as an independent risk factor for the development of diabetes and hypertension, and treatments to lower uric acid can reduce the burden of diabetes and lower blood pressure [75]. Moreover, a meta-analysis that examined middle-aged and older adults, found that after adjusting for metabolic syndrome, the association between uric acid level and diabetes mellitus increased substantially. Additionally, this association was shown to be stronger in Western societies [76]. However, there is still some debate regarding the role of elevated serum uric acid in the development of diabetes. There is evidence to support that it is a predictive risk factor preceding diabetes, as well as a protective response of glucose metabolism, with greater evidence mounting for the former [77, 78]. Our findings do not support one theory over another, but they do suggest that more research is needed to examine elevated serum uric acid as a possible marker for individuals at high-risk of developing diabetes.

Further comparisons were made for each risk factor, but no additional differences were found between those who progressed from normal FPG to either pre-diabetes or to diabetes. Our cross-sectional findings indicate no significant association between pre-diabetes and diabetes due to low HDL cholesterol, which does not support most research indicating low HDL cholesterol as a possible independent risk factor for diabetes [11,20, 23]. However, in the longitudinal analysis, risk of pre-diabetes was greater among those who remained with abnormally low HDL cholesterol levels over time (RRR 2.4 95% CI 1.3–4.4). A recent study of participants from the Copenhagen City Heart Study (CCHS) and the Copenhagen General Population Study, reported that abnormally low levels of HDL are not associated with an increased risk of developing diabetes in the general population [79]. Thus, our findings contribute to the conflicting results that have been reported concerning the association between HDL cholesterol and diabetes, and support the need for further research in this area.

Among the total sample of 1,174 participants, 16% had pre-diabetes and 10% had diabetes at W1 (2004–2006). By W2 (2011–2013), pre-diabetes had nearly doubled (30%), and the prevalence of diabetes increased to 16%. Among participants with pre-diabetes in W1, 53% remained with pre-diabetes and 28% progressed to diabetes. The increased prevalence of pre-diabetes corresponds to prior estimates for Mexico, while the increased prevalence of diabetes was lower in the study sample than in the general population [5]. Our results support the urgent need for primordial prevention of elevated glucose and failing prevention, the need for earlier screening and treatment of elevated blood glucose prior to the onset of pre-diabetes and diabetes. After considering factors such as sex, age, education status, and weight, patients who have MS, elevated triglycerides levels, and elevated ALT may benefit from routine monitoring of their blood glucose. More research on elevated serum uric acid as a marker for higher risk individuals is also warranted [78].

Research demonstrates that diabetes onset may be delayed by several years through diet and lifestyle modification [80,81]. However, pre-diabetes also poses health risks, such as long-term microvascular and macrovascular complications. These outcomes may not be fully preventable with medications and lifestyle interventions alone; regression to normal FPG is found to also be necessary [82,83]. Nonetheless, studies have found that lifestyle interventions in adults with pre-diabetes show a relative risk reduction of 40–70% for developing diabetes [84]. Risk of cardiovascular disease is increased with pre-diabetes and diabetes, but has been reduced by up to 56% over a 10- year period by regaining normal FPG, compared to remaining with pre-diabetes [42]. Furthermore, elevated FPG in the high normal range has been associated with poorer cognitive performance and decreased brain volume, further underscoring the importance of attaining normal blood glucose levels [85].

There are some limitations to this study. The Mexican health workers who participated in this cohort study were mostly women with a higher level of education and better health than the general population. They are considered middle-income, which corresponds to approximately 34% of the population. Because the sample is not representative of the entire Mexican population, our results should be considered exploratory. A second limitation is the large loss to follow-up between W1 and W2 (80%). Fortunately, there were no differences in several independent variables (i.e. sex, BMI, blood pressure, waist circumference, metabolic syndrome, elevated HDL cholesterol, triglycerides, and uric acid) between those who returned for W2 and those who did not, so we do not expect the low follow-up rate to affect our results. Additionally, we restricted our analyses to only participants with complete W1 and W2 data so our internal validity would not be compromised. A third limitation is the small sample size due to incomplete blood-related laboratory tests for participants at W2, which could affect power. Another limitation is the lack of uniform diagnostic criteria for pre-diabetes among the general population worldwide, which is a necessary prerequisite to conduct studies that can yield consensus treatment and prevention strategies [84].

The structure of the population in Latin America has been changing. Most of the people living in rural areas have migrated to urban cities, seeking better job opportunities. In Mexico, this demographic transition has been occurring for several decades, with the result that only about 20% of the population now lives in rural areas [86]. The lack of skills and knowledge among the populations moving to urban areas puts them at a disadvantage for employment opportunities and a good quality of life [87]. A nutrient-poor diet and lack of physical activity contribute to increasing rates of pre-diabetes and diabetes in the population. Within the next decade, the prevalence of diabetes is expected to increase by 38% in Latin America [88]. Furthermore, people with diabetes generally live shorter lives compared to those without diabetes, which can additionally exacerbate the social and economic consequences of diabetes [89].

The economic burden of diabetes in Mexico represents an enormous challenge for its health system. Diabetes involves direct (e.g. medication) and indirect costs (e.g. disability, shorter life expectancy, etc.). Arredondo et al. reported that in 2010, the total direct cost of the disease was $343 million USD and indirect costs were estimated at $177 million USD. However, these estimates do not include the costs due to the medical complications associated with diabetes, which lead to an additional economic burden [90]. Therefore, future studies should also focus on how diabetes prevention and early detection can help minimize the economic burden and improve the quality of life of these individuals. Mexico has implemented national diabetes action programs, since the prevention of non-communicable diseases has been a top priority in recent National Health plans, with a special emphasis on diabetes. Some of the recommended actions to confront this disease include, self-care diabetes campaigns, regulation of the food available in elementary schools, communication campaigns to raise awareness about diabetes, support groups, as well as standardized criteria and guidelines to improve the diagnosis and control of diabetes nationwide [90].

## Conclusions

Our results indicate that risk of developing diabetes among a group of Mexican health workers who were followed for approximately 7 years was associated with elevated BMI, triglycerides, blood pressure and uric acid, as well as the presence of metabolic syndrome. Most of these trends were observed among participants who had normal levels of FPG at the beginning of the study. These longitudinal findings confirm that serum uric acid is an independent risk factor for diabetes. The prevalence of diabetes among our study participants increased by more than 50% from the period of 2004–2006 (W1) until 2011–2013 (W2). Thus, we believe it imperative to design and implement interventions to prevent individuals from reaching the pre-diabetes stage, and failing that, to detect individuals at the pre-diabetes stage as soon as possible. This will allow them to make changes in their lifestyles that can perhaps delay or ideally prevent the onset of diabetes.

## Supporting information

S1 File Mexican Health Worker Cohort Study questionnaire, 2010–2011. (PDF)
