# Supplementary material for: Risk of developing pre-diabetes or diabetes over time in a cohort of Mexican health workers
Source: PLoS One. 2020 Mar 25;15(3):e0229403. doi: 10.1371/journal.pone.0229403 (PMC7094846; doi:10.1371/journal.pone.0229403)
Supplement: S1 File — (PDF) [file pone.0229403.s001.pdf]

Portada
IMSS adultos

Instituto Mexicano del Seguro Social
Seguridad y Solidaridad Social

Cohorte de Trabajadores de la Salud

Cohorte de Trabajadores del Instituto Mexicano del Seguro Social

Etapa 2010 - 2011

IMSS Seguridad y Solidaridad Social
ISEM
Instituto Nacional de Medicina Genómica MEXICO

Cohorte de Trabajadores del Instituto Mexicano del Seguro Social
Etapa 2010 - 2011

Estimado trabajador, queremos expresarle nuestro más sincero agradecimiento por haber aceptado participar en el proyecto de Cohorte de Trabajadores del IMSS. Estamos llevando a cabo una nueva etapa de este estudio, para participar en este seguimiento es necesario que responda este cuestionario, las preguntas que se le harán pretenden documentar en forma detallada algunos antecedentes de interés para evaluar condiciones de salud especiales. Cuenta usted con tres semanas para contestar, lo más conveniente es que lo haga cuando pueda concentrarse en las preguntas y responda lo más apegado posible a lo que es o ha sido su salud, es por esto que son sus respuestas las que cuentan, y no las de las otras personas.

Instrucciones

Antes de empezar a contestar lea con cuidado las instrucciones, en cada sección puede haber indicaciones especiales que es necesario atender. Lea con atención cada pregunta, antes de responder vea las opciones de respuesta, seleccione la que le parezca más apegada a su realidad. Para marcar su respuesta sólo use lápiz del No. 2, rellene completamente el círculo sin salirse de sus bordes. Sus respuestas serán "leídas" por una máquina llamada lector óptico, esta máquina sólo "puede leer" su respuesta si se usa lápiz y se rellena correctamente el círculo. Cuando se le solicite escribir, use letra de molde o imprenta, sin rebasar el espacio asignado. Si desea cambiar su respuesta tiene que borrar por completo, sin romper el papel y marcar su nueva respuesta. Encontrará cuadros con círculos que dicen NO RELLENAR, por favor no marque estos círculos.

Existen preguntas que están relacionadas o enlazadas, cuando son de este tipo están unidas con una flecha. A veces hay preguntas que nos pueden brincar a otra pregunta, por ejemplo ¿Ha fumado 100 cigarrillos o más en toda su vida?, tiene tres posibles respuestas: No, Sí y actualmente fumo y Sí fumé, pero actualmente ya no fumo. En la opción No, aparece una instrucción de pasar a la siguiente pregunta; debido a que la persona que nunca ha fumado no tiene por qué contestar las preguntas que se refieren al hábito de fumar. Si la respuesta elegida fue: Sí y actualmente fumo; el círculo está relleno por completo, esta respuesta se enlaza con la flecha que lleva al recuadro que solicita que especifique la marca y tipo de cigarrillos, la cual se contestó con letra de molde en el cuadro asignado. El cuadro que dice NO RELLENAR, no fue marcado.

¿Ha fumado 100 cigarrillos o más en toda su vida?

○ No, nunca he fumado (pase a la siguiente pregunta)

○ Sí fumé, pero actualmente ya no fumo

● Sí y actualmente fumo

Especifique la marca y el tipo de cigarrillos (por ej. Marlboro lights)	Delicados con filtro<br>Especifique qué marca<br>NO RELLENAR<br>0 1 2 3 4 5 6 7 8 9<br>0 1 2 3 4 5 6 7 8 9

Para esta pregunta hay que responder cuál es la figura que mejor representa a su madre (en caso de que todavía viva), así como la edad que tiene actualmente. Si su madre tuviera 58 años, hay que escribir esta edad en el espacio asignado, además hay que rellenar el círculo correspondiente como se presenta en el ejemplo.

Si su madre es muy delgada seguramente la figura que mejor la refleja sería una de la extrema izquierda y deberá rellenar uno de estos círculos, como se muestra en el ejemplo.

55. Datos de su madre
De las figuras que aparecen a continuación, por favor señale cuál representa mejor figura de su madre actualmente (no responda si su madre ya no vive)

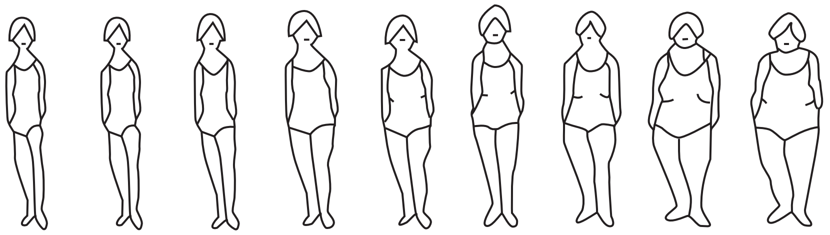

● ○ ○ ○ ○ ○ ○ ○ ○

¿Qué edad tiene su madre actualmente?

| 5 | 8 | años

1 2 3 4 ● 6 7 8 9
0 1 2 3 4 5 6 7 ● 9

En ocasiones es necesario especificar la actividad a la que se dedica, alguna molestia que usted tiene o algún alimento que come, etc.

En este ejemplo, si su respuesta fuera Sí, seguiría la flecha y tendría qué especificar la margarina que consume.

¿Consume margarina comúnmente?

● **Sí** ○ **No**

**Primavera**<br>**especifique marca**	**NO RELLENAR**<br>0 1 2 3 4 5 6 7 8 9<br>0 1 2 3 4 5 6 7 8 9

Cohorte de Trabajadores del Instituto Mexicano del Seguro Social
Etapa 2010 - 2011

No lo olvide, use lápiz del No. 2 para contestar todas las preguntas. Es importante respetar los espacios señalados y no hacer marcas fuera de ellos. Rellene el círculo completamente de esta forma ●, no marque así: ⊗ ⊘ ◐ ◉ porque su respuesta no será leída correctamente por el lector óptico.

Cuando haya terminado de contestar una sección, cheque que todas las preguntas tengan su respuesta. Si desea hacer comentarios, por favor escríbanos en una hoja por separado y anéxela al cuestionario, por favor entregue el cuestionario en el módulo en donde se le dio inicialmente el cuestionario.

Recuerde, es muy importante que responda a todas las preguntas.
Gracias por participar en el estudio Cohorte de Trabajadores del Instituto Mexicano del Seguro Social

Aspectos Generales, Personales y Familiares

Escriba su fecha de nacimiento en el espacio vacío de la parte superior de la caja.
Por ejemplo: 6 de septiembre de 1921, sería

Note que para cada cifra, se rellena el círculo que corresponda en esta forma.

1.- Fecha de nacimiento (ejemplo)

Día		Mes		Año			
0	6	0	9	1	9	2	1
●	0	●	0	0	0	0	0
1	1	1	1	●	1	1	●
2	2	2	2	2	2	●	2
3	3	3	3		3	3	3
	4		4		4	4	4
	5		5		5	5	5
	●		6		6	6	6
	7		7		7	7	7
	8		8		8	8	8
	9		●		●	9	9

Siguiendo el ejemplo, escriba su fecha de nacimiento →

1) NO RELLENAR
0 1 2 3 4 5 6 7 8 9
0 1 2 3 4 5 6 7 8 9
0 1 2 3 4 5 6 7 8 9
0 1 2 3 4 5 6 7 8 9
0 1 2 3 4 5 6 7 8 9

1.- Fecha de nacimiento

Día		Mes		Año			
0	0	0	0	0	0	0	0
1	1	1	1	1	1	1	1
2	2	2	2	2	2	2	2
3	3	3	3		3	3	3
	4		4		4	4	4
	5		5		5	5	5
	6		6		6	6	6
	7		7		7	7	7
	8		8		8	8	8
	9		9		9	9	9

¿Cuál es su C.U.R.P.? |_|_|_|_|_|_|_|_|_|_|_|_|_|_|_|_|_|_|

2.- ¿Cuál es su estado civil?
- ◯ Casado (a)
- ◯ Viudo (a)
- ◯ Separado (a)
- ◯ Soltero (a)
- ◯ Unión libre
- ◯ Divorciado (a)

3.- Indique con quién vive
- ◯ Solo (a) (Pase a al pregunta 4)
- ◯ Solo (a) con sus hijos
- ◯ Con su esposo (a) e hijos
- ◯ Con sus padres
- ◯ Con otra familia
- ◯ Otro: ____________ (Especifique)

¿Cuántas personas viven en total con usted?
0 1 2 3 4 5 6
0 1 2 3 4 5 6 7 8 9 |_|_| personas

¿Cuántas personas que viven con usted en su casa son menores de 12 años y/o mayores de 60?
0 1 2 3 4 5 6
0 1 2 3 4 5 6 7 8 9 |_|_| personas

3) NO RELLENAR
0 1 2 3 4 5 6 7 8 9

4.- Si usted trabaja en el IMSS especifique cuál de las siguientes categorías describe mejor su puesto de trabajo:

- ◯ Jubilado - Pensionado
- ◯ Enfermera
- ◯ Asistente Médica
- ◯ Auxiliar de Administración (AUO)
- ◯ Auxiliar de Farmacia
- ◯ Personal Administrativo
- ◯ Auxiliar de Intendencia
- ◯ Jefe de Servicio
- ◯ Investigador
- ◯ Laboratorio
- ◯ Cajera
- ◯ Chofer
- ◯ Especialidad Técnica
- ◯ Orientador de Actividades Artísticas
- ◯ Trabajo Social
- ◯ Personal de Conservación
- ◯ Educador Físico
- ◯ Promotora Voluntaria
- ◯ Becario - Estudiante
- ◯ Médico Familiar
- ◯ Médico No Familiar
- ◯ Nutriólogo - Dietista - Puericultura
- ◯ Otro: ____________ (Especifique)

4a) NO RELLENAR
0 1 2 3 4 5 6 7 8 9

4b) NO RELLENAR
0 1 2 3 4 5 6 7 8 9

¿Cuál es su matrícula? |_|_|_|_|_|_|_|

¿Cuál es su condición laboral?
- ◯ Base
- ◯ Confianza
- ◯ 08
- ◯ Otra: ____________ (Especifique)

5) NO RELLENAR
0 1 2 3 4 5 6 7 8 9

5.- Si usted no trabaja en el IMSS, ¿a qué se dedica?
(Especifique) ____________

¿Hasta qué año fue a la escuela?

	Completa	Incompleta
◯ Primaria	◯	◯
◯ Secundaria	◯	◯
◯ Preparatoria	◯	◯
◯ Normal superior	◯	◯
◯ Profesional	◯	◯
◯ Posgrado	◯	◯

Antecedentes Heredofamiliares

6.- Algún familiar ha padecido o padece

	Abuelos paternos	Abuelos maternos	Madre	Padre	Hermanos	Hijos
**¿Hipertensión arterial?**						
◯ **Sí (quién)** →	◯	◯	◯	◯	◯	◯
◯ **No**						
◯ **No sabe**						
**¿Diabetes mellitus?**						
◯ **Sí (quién)** →	◯	◯	◯	◯	◯	◯
◯ **No**						
◯ **No sabe**						
**¿Infarto al miocardio?**						
◯ **Sí (quién)** →	◯	◯	◯	◯	◯	◯
◯ **No**						
◯ **No sabe**						
**¿Cáncer de mama?**						
◯ **Sí (quién)** →	◯	◯	◯	◯	◯	◯
◯ **No**						
◯ **No sabe**						
**¿Cáncer de ovario?**						
◯ **Sí (quién)** →	◯	◯	◯	◯	◯	◯
◯ **No**						
◯ **No sabe**						
**¿Cáncer de endometrio?**						
◯ **Sí (quién)** →	◯	◯	◯	◯	◯	◯
◯ **No**						
◯ **No sabe**						
**¿Cáncer de colon?**						
◯ **Sí (quién)** →	◯	◯	◯	◯	◯	◯
◯ **No**						
◯ **No sabe**						
**¿Tumor cerebral?**						
◯ **Sí (quién)** →	◯	◯	◯	◯	◯	◯
◯ **No**						
◯ **No sabe**						
**¿Leucemia?**						
◯ **Sí (quién)** →	◯	◯	◯	◯	◯	◯
◯ **No**						
◯ **No sabe**						
**¿Osteoporosis?**						
◯ **Sí (quién)** →	◯	◯	◯	◯	◯	◯
◯ **No**						
◯ **No sabe**						
**¿Fractura de cadera?**						
◯ **Sí (quién)** →	◯	◯	◯	◯	◯	◯
◯ **No**						
◯ **No sabe**						
**¿Fractura de muñeca?**						
◯ **Sí (quién)** →	◯	◯	◯	◯	◯	◯
◯ **No**						
◯ **No sabe**						

7.- ¿Cuántos dientes propios tiene?

◯ **Ninguno** ◯ **11 a 16** ◯ **25 a 32**
◯ **1 a 10** ◯ **17 a 24**

¿Durante los últimos dos años cuántos dientes permanentes perdió?

◯ **0** ◯ **3-4** ◯ **Más de 10**
◯ **1-2** ◯ **5-10**

¿Durante los últimos dos años en cuántos dientes le han hecho endodoncia?

◯ **0** ◯ **3-4** ◯ **Más de 10**
◯ **1-2** ◯ **5-10**

8.- ¿Ha fumado 100 cigarrillos o más en toda su vida?

◯ No, nunca he fumado (pase a la pregunta 14)
◯ Sí fumé, pero actualmente ya no fumo
◯ Sí y actualmente fumo

→ Especifique la marca y el tipo de cigarrillos (por ej. Marlboro lights)

____
(Especifique)

8a) NO RELLENAR
⓪ ① ② ③ ④ ⑤ ⑥ ⑦ ⑧ ⑨
⓪ ① ② ③ ④ ⑤ ⑥ ⑦ ⑧ ⑨

→ **¿A qué edad fumó su primer cigarrillo?**

⓪ ① ② ③ ④ ⑤
⓪ ① ② ③ ④ ⑤ ⑥ ⑦ ⑧ ⑨ |__|__| años

→ **¿Cuántos cigarros fuma al día?**

⓪ ① ② ③ ④ ⑤
⓪ ① ② ③ ④ ⑤ ⑥ ⑦ ⑧ ⑨ |__|__| años

→ **¿Hace cuánto tiempo dejó de fumar?**

◯ < 1 año ◯ 1 a 2 años ◯ Más de 2 años

→ **¿Por qué dejó de fumar?**

◯ Por conciencia de su daño
◯ Por prescripción médica
◯ Porque se hartó de fumar
◯ Porque le causó problemas con la familia
◯ Por restricciones en su trabajo u otros lugares
◯ Otra razón ____________________
(Especifique)

8b) NO RELLENAR
⓪ ① ② ③ ④ ⑤ ⑥ ⑦ ⑧ ⑨

→ **Para cada edad, marca el número de cigarrillos que fumaba al día o que fuma actualmente:**

	Ninguno	1-5	6-14	15-24	25-34	35-44	45 y +
< 15 años	◯	◯	◯	◯	◯	◯	◯
15-19 años	◯	◯	◯	◯	◯	◯	◯
20-29 años	◯	◯	◯	◯	◯	◯	◯
30-39 años	◯	◯	◯	◯	◯	◯	◯
40-49 años	◯	◯	◯	◯	◯	◯	◯
50-59 años	◯	◯	◯	◯	◯	◯	◯
60 ó más años	◯	◯	◯	◯	◯	◯	◯
Actualmente	◯	◯	◯	◯	◯	◯	◯

9.- ¿En su casa convive con personas que fuman?

◯ Sí ◯ No (pase a la siguiente pregunta)

¿Cree qué esto afecta su salud?

◯ Sí ◯ No

¿De los habitantes de su casa cuántos fuman?

⓪ ① ② ③ ④ ⑤ ⑥
⓪ ① ② ③ ④ ⑤ ⑥ ⑦ ⑧ ⑨ personas

¿Cree qué esto afecta la salud de los miembros de la familia que no fuman?

◯ Sí ◯ No

10.- ¿Convive en su trabajo con alguien que fuma?

◯ Sí ◯ No (pase a la pregunta 13)
◯ No trabajo (pase a la pregunta 13)

¿Dentro de su trabajo, el área donde convive con fumadores, es?

◯ Cerrada ◯ Ventilada

¿Está prohibido fumar en su área de trabajo?

◯ Sí ◯ No ◯ No sé

¿Existe señalamiento de prohibición de fumar, en su área de trabajo?

◯ Sí ◯ No ◯ No sé

¿Dentro de su edificio o trabajo hay un sitio especial para fumadores?

◯ Sí ◯ No ◯ No sé

¿Conoce acerca de la disposición de Edificios Libres de Humo?

◯ Sí ◯ No

¿Le gustaría que esta disposición se implante en su trabajo?

◯ Sí ◯ No

¿En caso de que fume, piensa que esta disposición le ayudará a dejar de fumar?

◯ Sí ◯ No ◯ No sé

11.- ¿Usted fuma durante su jornada laboral?

◯ Sí ◯ No (pase a pregunta 13) ◯ No Trabajo (pase a la pregunta 13)

¿Cuántos cigarros al día fuma en su trabajo?

⓪ ① ② ③ ④ ⑤ ⑥
⓪ ① ② ③ ④ ⑤ ⑥ ⑦ ⑧ ⑨ cigarros

Estos espacios le parecen:

◯ Muy confortables
◯ Confortables
◯ Indistinto
◯ Poco confortables
◯ Desagradables

¿Ha recibido sanciones por no acatar la disposición de no fumar?

◯ Sí ◯ No

¿En su trabajo cuál es el lugar que más frecuentemente usa para fumar?

◯ El pasillo
◯ La escalera
◯ Mi oficina
◯ El baño
◯ Otro: ____________________ (Especifíque)

11) NO RELLENAR
⓪ ① ② ③ ④ ⑤ ⑥ ⑦ ⑧ ⑨

¿Cuántas veces al día acude a ese lugar?

⓪ ① ② ③ ④ ⑤ ⑥ ⑦ ⑧ ⑨
⓪ ① ② ③ ④ ⑤ ⑥ ⑦ ⑧ ⑨ veces

¿Existen señalamientos que identifiquen los espacios asignados a los fumadores?

◯ Sí ◯ No ◯ No sé

¿Le han dado orientación sobre los riesgos que implica para su salud el ser fumador?

◯ Sí ◯ No ◯ No sé

¿Durante el último año le han ofrecido ayuda médica o psicológica para dejar de fumar?

◯ Sí ◯ No ◯ No sé

12.- ¿En su lugar de trabajo considera que se cumple actualmente con el Reglamento sobre Consumo de Tabaco?

◯ Sí ◯ No (pase a pregunta 13) ◯ No Trabajo (pase a la pregunta 13)

¿Qué tan de acuerdo se encuentra con esta medida?

◯ Totalmente de acuerdo
◯ De acuerdo
◯ Indiferente
◯ En desacuerdo
◯ Totalmente en desacuerdo

12) NO RELLENAR
⓪ ① ② ③ ④ ⑤ ⑥ ⑦ ⑧ ⑨

¿En su lugar de trabajo le han dado a conocer el reglamento sobre Consumo de Tabaco en Edificios Públicos?

◯ Sí ◯ No

¿En los planes de estudio de las facultades de medicina y enfermería se incluye información sobre consumo de tabaco?

◯ Sí ◯ No ◯ No sé

¿Considera necesario abordar estos temas en la formación profesional de los trabajadores de la salud?

◯ Sí ◯ No ◯ No sé

¿Cómo fue que conoció estas disposiciones?

◯ Carteles - Folletos
◯ Mensajes de televisión
◯ Mensajes de radio
◯ Oficio/circular
◯ A través de jefes superiores
◯ Por compañeros
◯ Pláticas
◯ Otros: ____________________ (Especifíque)

13.- ¿En los últimos 6 meses ha usado parches de nicotina o goma de mascar con nicotina para dejar de fumar?

◯ Sí ◯ No

◯ Parches
◯ Goma de mascar

¿Cuánto tiempo los usó?

meses
⓪ ① ② ③ ④ ⑤ ⑥ ⑦ ⑧ ⑨
⓪ ① ② ③ ④ ⑤ ⑥ ⑦ ⑧ ⑨

¿Desde que se levanta cuánto tiempo pasa antes de que fume su primer cigarrillo?

◯ Menos de 5 minutos ◯ De 6 a 30 minutos
◯ De 31 a 60 minutos ◯ Más de 60 minutos

¿Cuál cigarrillo le costaría más trabajo dejar de fumar?

◯ El primero de la mañana ◯ Otros durante el día

¿Cuando usted se enferma y tiene que permanecer en cama, fuma?

◯ Sí ◯ No

¿Le cuesta trabajo abstenerse de fumar en los lugares donde está prohibido, por ejemplo, transporte público, cines, centros comerciales, escuelas, etc.?

◯ Sí ◯ No

¿Fuma más por la mañana que el resto del día?

◯ Sí ◯ No

14.- ¿En qué lugar nació?

◯ Aguascalientes	◯ Distrito Federal	◯ Morelos	◯ Sinaloa
◯ Baja California Norte	◯ Durango	◯ Nayarit	◯ Sonora
◯ Baja California Sur	◯ Guanajuato	◯ Nuevo León	◯ Tabasco
◯ Campeche	◯ Guerrero	◯ Oaxaca	◯ Tamaulipas
◯ Coahuila	◯ Hidalgo	◯ Puebla	◯ Tlaxcala
◯ Colima	◯ Jalisco	◯ Querétaro	◯ Veracruz
◯ Chihuahua	◯ Edo. México	◯ Quintana Roo	◯ Yucatán
◯ Chiapas	◯ Michoacán	◯ San Luis Potosi	◯ Zacatecas
			◯ Extranjero

¿Cuántos años lleva viviendo en donde vive actualmente?

|__|__| años

⓪ ① ② ③ ④ ⑤ ⑥ ⑦ ⑧ ⑨
⓪ ① ② ③ ④ ⑤ ⑥ ⑦ ⑧ ⑨

Sección de utilización de Servicios de Salud

Desde el punto de vista del estudio de la salud de las poblaciones, el uso de los servicios de salud puede dar un panorama general de la conducta preventiva y del patrón de utilización que realiza la población en función de sus necesidades o problemas de salud. Estas condiciones de salud pueden motivar en primera instancia el uso de servicios de prevención, en segunda los servicios de diagnóstico y tratamiento y, en última instancia, los de mantenimiento y rehabilitación. Es asi que la utilización de servicios de salud es considerada como una sucesión de acontecimientos que va desde el momento que surge la necesidad de atención hasta el momento en que se inicia o se continúa dicha utilización. En el presente estudio en el que ustedes, los participantes, son trabajadores de una institución que cuenta con un esquema integral de prestación de servicios de salud, es de gran utilidad contar con información sobre el patrón de uso de servicios de salud, que permita plantear nuevas formas de organizar los servicios de salud para mejorar las opciones que actualmente se le ofrecen.

A continuación le haremos algunas preguntas sobre los servicios de salud que ha utilizado durante los últimos meses.

15.- ¿Tuvo algún problema de salud durante los últimos 6 meses?

◯ Sí ◯ No

→ **¿Dejó de trabajar algunos días por este problema de salud?**

◯ Sí → ¿Cuánto tiempo? |__|__| semanas

◯ No

¿Qué problema fue? ________________________________
(Especifíque)

15a) NO RELLENAR
⓪ ① ② ③ ④ ⑤ ⑥ ⑦ ⑧ ⑨
⓪ ① ② ③ ④ ⑤ ⑥ ⑦ ⑧ ⑨

15b) NO RELLENAR
⓪ ① ② ③ ④ ⑤ ⑥ ⑦ ⑧ ⑨
⓪ ① ② ③ ④ ⑤ ⑥ ⑦ ⑧ ⑨

16.- ¿Tuvo usted alguna consulta con el médico en los últimos 12 meses?

◯ Sí ◯ No

→ **¿Hace cuánto tiempo fue esta consulta?** |__|__| semanas

16) NO RELLENAR
⓪ ① ② ③ ④ ⑤ ⑥ ⑦ ⑧ ⑨
⓪ ① ② ③ ④ ⑤ ⑥ ⑦ ⑧ ⑨

17.- La última vez que visitó al médico fue por:

◯ Enfermedad
◯ Prueba(s) o estudio(s) de control
◯ Revisión (chequeo general)
◯ Otro:______________________
(Especifíque)

17) NO RELLENAR
⓪ ① ② ③ ④ ⑤ ⑥ ⑦ ⑧ ⑨

**18.- ¿Usó usted alguno de los siguientes servicios de salud durante los últimos 12 meses?**	**Sí**	**No**	**Número de veces**	
Consulta externa al médico familiar o general	◯	◯	|__|__| veces	18a) NO RELLENAR ⓪ ① ② ③ ④ ⑤ ⑥ ⑦ ⑧ ⑨
Consulta externa al médico especialista	◯	◯	|__|__| veces	18b) NO RELLENAR ⓪ ① ② ③ ④ ⑤ ⑥ ⑦ ⑧ ⑨
Consulta de Nutrición	◯	◯	|__|__| veces	18c) NO RELLENAR ⓪ ① ② ③ ④ ⑤ ⑥ ⑦ ⑧ ⑨
Consulta de Psicología	◯	◯	|__|__| veces	18d) NO RELLENAR ⓪ ① ② ③ ④ ⑤ ⑥ ⑦ ⑧ ⑨
Consulta de Odontología	◯	◯	|__|__| veces	18e) NO RELLENAR ⓪ ① ② ③ ④ ⑤ ⑥ ⑦ ⑧ ⑨
Consulta en Urgencias	◯	◯	|__|__| veces	18f) NO RELLENAR ⓪ ① ② ③ ④ ⑤ ⑥ ⑦ ⑧ ⑨
Medicina preventiva (detecciones, vacunas, estudios epidemiológicos)	◯	◯	|__|__| veces	18g) NO RELLENAR ⓪ ① ② ③ ④ ⑤ ⑥ ⑦ ⑧ ⑨
Laboratorio	◯	◯	|__|__| veces	18h) NO RELLENAR ⓪ ① ② ③ ④ ⑤ ⑥ ⑦ ⑧ ⑨
Gabinete (rayos X, ultrasonido, etc.)	◯	◯	|__|__| veces	18i) NO RELLENAR ⓪ ① ② ③ ④ ⑤ ⑥ ⑦ ⑧ ⑨
Sesiones de educación para la salud (Talleres o grupos de ayuda)	◯	◯	|__|__| veces	18j) NO RELLENAR ⓪ ① ② ③ ④ ⑤ ⑥ ⑦ ⑧ ⑨
Actividades de promoción a la salud (yoga, danza, manualidades, etc.)	◯	◯	|__|__| veces	18k) NO RELLENAR ⓪ ① ② ③ ④ ⑤ ⑥ ⑦ ⑧ ⑨
Otro: ____________ (Especifíque) 18l) NO RELLENAR ⓪ ① ② ③ ④ ⑤ ⑥ ⑦ ⑧ ⑨	◯	◯	|__|__| veces	18m) NO RELLENAR ⓪ ① ② ③ ④ ⑤ ⑥ ⑦ ⑧ ⑨

19.- ¿Estuvo usted hospitalizado o internado por alguno de los siguientes motivos en instituciones de salud durante los últimos 12 meses?	Sí	No	Número de veces	
Cirugía (Especifíque) ____ NO RELLENAR 0 1 2 3 4 5 6 7 8 9	◯	◯	veces	19a) NO RELLENAR 0 1 2 3 4 5 6 7 8 9
Enfermedad (Especifíque) ____ NO RELLENAR 0 1 2 3 4 5 6 7 8 9	◯	◯	veces	19b) NO RELLENAR 0 1 2 3 4 5 6 7 8 9
Lesión física por accidente dentro de la casa	◯	◯	veces	19c) NO RELLENAR 0 1 2 3 4 5 6 7 8 9
Lesión física por accidente fuera de la casa	◯	◯	veces	19d) NO RELLENAR 0 1 2 3 4 5 6 7 8 9
Lesión física por agresión o violencia dentro de la casa	◯	◯	veces	19e) NO RELLENAR 0 1 2 3 4 5 6 7 8 9
Lesión física por agresión o violencia fuera de la casa	◯	◯	veces	19f) NO RELLENAR 0 1 2 3 4 5 6 7 8 9
Otro motivo (Especifique) ____ NO RELLENAR 0 1 2 3 4 5 6 7 8 9	◯	◯	veces	19g) NO RELLENAR 0 1 2 3 4 5 6 7 8 9

20.- ¿En los últimos 12 meses, ha utilizado algún servicio en centros de Seguridad Social, Centros Culturales, Casas de día, Centros de bienestar familiar o Centros deportivos?

◯ Sí ◯ No

¿Qué servicios o actividades sociales ha utilizado o realizado?	Sí	No	Número de veces	
Cursos o eventos de cultura para la salud como: actividad o acondicionamiento físico, jornadas nacionales, caminatas para la salud.	◯	◯	veces	20a) NO RELLENAR 0 1 2 3 4 5 6 7 8 9
Campañas de higiene para la salud como: higiene personal o prevención	◯	◯	veces	20b) NO RELLENAR 0 1 2 3 4 5 6 7 8 9
Cursos, eventos o campañas de bienestar social como: cocina, aeróbics, baile, artesanías, prevención de accidentes, exposiciones, concursos y festivales culturales y muestras artísticas	◯	◯	veces	20c) NO RELLENAR 0 1 2 3 4 5 6 7 8 9
Cursos de superación de nivel de vida como: corte y confección, juguetería, decoración.	◯	◯	veces	20d) NO RELLENAR 0 1 2 3 4 5 6 7 8 9
Otro: ____ (Especifique)			veces	20e) NO RELLENAR 0 1 2 3 4 5 6 7 8 9

¿Por qué motivo no ha usado servicios sociales o realizado actividades sociales? (puede seleccionar más de una opción)

◯ No tiene tiempo para realizar esas actividades.
◯ No puede realizar ese tipo de actividades por alguna limitación física.
◯ No le gustan las actividades que se ofrecen.
◯ Los lugares donde se realizan esas actividades quedan lejos de su casa.
◯ No sabía que podía usar esos servicios o realizar ese tipo de actividades.
◯ No cuenta con recursos económicos para realizarlas.

20f) NO RELLENAR
0 1 2 3 4 5 6 7 8 9
0 1 2 3 4 5 6 7 8 9

Si usted participó en la etapa anterior de este estudio, por favor responda las siguientes preguntas:

21.- Cuando recibió los resultados de los análisis practicados en la fase anterior del estudio, usted:

¿Acudió al médico?

◯ No (pase a la siguiente pregunta) ◯ Sí

Tomó algún medicamento

◯ No ◯ Sí ¿Cuál?: ____ (Especifique)

Su médico le dio alguna indicación sobre:

◯ Bajar de peso
Siguió la indicación ◯ No ◯ Sí

◯ Disminuir el consumo de ciertos alimentos
Siguió la indicación ◯ No ◯ Sí

◯ Aumentar el tiempo dedicado a realizar ejercicio
Siguió la indicación ◯ No ◯ Sí

◯ Dejar de fumar
Siguió la indicación ◯ No ◯ Sí

21) NO RELLENAR
0 1 2 3 4 5 6 7 8 9
0 1 2 3 4 5 6 7 8 9

22.- ¿Qué medicamentos toma actualmente?

(Marque sólo los que usa regularmente, por ejemplo, 2 ó más veces por semana).

- ○ **Ninguno (Pase a la pregunta 23).**
- ○ Ácido Acetil Salicílico (ej. Aspirina).
- ○ Acetaminofén (ej. Tempra).
- ○ Antiinflamatorios (ej. Diclofenaco, Naproxeno, Ibuprofeno, Piroxicam).
- ○ Diuréticos como Furosemida (ej. Lasix).
- ○ Clortalidona (ej. Higotrón).
- ○ Espironolactona (ej. Aldactone).
- ○ Beta bloqueadores (ej. Propanolol, Metoprolol).
- ○ Bloqueadores de calcio (ej. Nifedipina, Adalat, Verapamilo).
- ○ Nitratos (ej. Isosorbide).
- ○ Otros antihipertensivos (ej. Alfa metil dopa, Hidralazina, Enalapril, Prasozina).
- ○ Digoxina (ej. Lanoxin).
- ○ Antiarrítmicos (ej. Norpace, Propofenona, Amiadarona, Quinidina).
- ○ Cimetidina, Ranitidina (ej. Tagamet, Ranisen).
- ○ Anticolesterolémicos (ej. Pravastatina, Bezafibrato).
- ○ Fibra natural como Psillium plántago (ej. Metamucil,Psillumax).
- ○ Hipoglucemiantes (ej. Tolbutamida, Glibenclamida).
- ○ Hormonales (ej. Estrógenos, Premarín). .
- ○ Antidepresivos (ej. Monclobamida, Imipramina).
- ○ Ansiolíticos (ej. Diazepán, Clonazepán, Alprazolán).
- ○ Metformín
- ○ Otro: ____________________ (Especifique)

22) NO RELLENAR
⓪ ① ② ③ ④ ⑤ ⑥ ⑦ ⑧ ⑨
⓪ ① ② ③ ④ ⑤ ⑥ ⑦ ⑧ ⑨

23a) NO RELLENAR
⓪ ① ② ③ ④ ⑤ ⑥ ⑦ ⑧ ⑨
⓪ ① ② ③ ④ ⑤ ⑥ ⑦ ⑧ ⑨

23b) NO RELLENAR
⓪ ① ② ③ ④ ⑤ ⑥ ⑦ ⑧ ⑨
⓪ ① ② ③ ④ ⑤ ⑥ ⑦ ⑧ ⑨

23c) NO RELLENAR
⓪ ① ② ③ ④ ⑤ ⑥ ⑦ ⑧ ⑨
⓪ ① ② ③ ④ ⑤ ⑥ ⑦ ⑧ ⑨

23.- Por favor marque si se ha sometido a alguna de las siguientes cirugías o si algún médico le ha dicho que tuvo o que tiene alguna de las siguientes enfermedades y el año en que se lo mencionó por primera vez:

**Sólo marque lo que sí tiene o ha tenido**	Antes del 2000	Del 2000 al 2007	Durante 2008	Durante 2009	Durante 2010	Durante 2011
Diabetes	○	○	○	○	○	○
Presión alta	○	○	○	○	○	○
Colesterol elevado	○	○	○	○	○	○
Triglicéridos elevados	○	○	○	○	○	○
Angina de pecho	○	○	○	○	○	○
Infarto al miocardio	○	○	○	○	○	○
Cirugía de arterias coronarias	○	○	○	○	○	○
Taquicardia supraventricular	○	○	○	○	○	○
Otra enfermedad del corazón	○	○	○	○	○	○
Claudicación intermitente	○	○	○	○	○	○
Trombosis venosa periférica	○	○	○	○	○	○
Infarto cerebral	○	○	○	○	○	○
Embolia cerebral	○	○	○	○	○	○
Bronquitis crónica	○	○	○	○	○	○
Embolia pulmonar	○	○	○	○	○	○
Asma	○	○	○	○	○	○
Úlcera gástrica	○	○	○	○	○	○
Úlcera duodenal	○	○	○	○	○	○
Cirrosis hepática	○	○	○	○	○	○
Cálculos (piedras) vesiculares	○	○	○	○	○	○
Colecistectomía (operación de vesícula)	○	○	○	○	○	○
Cálculos (piedras) en el riñón	○	○	○	○	○	○
Insuficiencia renal crónica	○	○	○	○	○	○
Apendicectomía	○	○	○	○	○	○
Cataratas	○	○	○	○	○	○
Extracción de catarata	○	○	○	○	○	○
Glaucoma	○	○	○	○	○	○
Gota	○	○	○	○	○	○
Artritis reumatoide	○	○	○	○	○	○
Artritis degenerativa	○	○	○	○	○	○
Osteoporosis	○	○	○	○	○	○
Fractura de cadera o fémur	○	○	○	○	○	○
Fractura de muñeca	○	○	○	○	○	○
Enfermedad de Parkinson	○	○	○	○	○	○
Melanoma	○	○	○	○	○	○
Cáncer de la piel	○	○	○	○	○	○
Cáncer gástrico	○	○	○	○	○	○
Cáncer de colon o recto	○	○	○	○	○	○
Linfoma	○	○	○	○	○	○
Leucemia	○	○	○	○	○	○
Cáncer de pulmón	○	○	○	○	○	○
Cáncer de mama	○	○	○	○	○	○
Quistes benignos en mama	○	○	○	○	○	○
Cáncer de endometrio	○	○	○	○	○	○
Cáncer del cuello de la matriz	○	○	○	○	○	○
Cáncer de ovario	○	○	○	○	○	○
Cáncer de próstata	○	○	○	○	○	○
Accidente de tránsito con fractura u hospitalización	○	○	○	○	○	○
Hepatitis B o C	○	○	○	○	○	○
Depresión	○	○	○	○	○	○
Otra enfermedad mental	○	○	○	○	○	○
(Especifique) ____________	○	○	○	○	○	○
Otras enfermedades	○	○	○	○	○	○
(Especifique) ____________	○	○	○	○	○	○
(Especifique) ____________	○	○	○	○	○	○

Sección de Alimentación

Esta sección del cuestionario está diseñada para conocer sus hábitos alimentarios. Lo recomendable es que cada una de las secciones del cuestionario se responda cuando usted este tranquilo (a) y tenga tiempo suficiente para concentrarse en lo que se le pide contestar.

La alimentación es un proceso que cambia a través del tiempo, por lo que esta sección del cuestionario es indispensable que se registre en cada etapa del estudio, registrar los cambios o consistencias en su patrón de alimentación nos resulta muy útil para cumplir con los objetivos del proyecto. Por lo que le pedimos que responda nuevamente esta sección del cuestionario.

Responda cada pregunta lo mejor que pueda. Si no está seguro de alguna respuesta, proporcione una aproximación o cálculo del consumo de los alimentos que se incluyen en el cuestionario. Realizar alguna aproximación será mejor que no contestar nada.

Marque el círculo que indica la frecuencia con que comió cada uno de los siguientes alimentos durante los últimos 12 meses

24.- Productos Lácteos	Promedio consumido durante los últimos 12 meses									
	Nunca	Menos de 1 vez x mes	1 - 3 x sem.	1 x sem.	2 - 4 x sem.	5 - 6 x sem.	1 x día	2 - 3 x día	4 - 5 x día	6 ó más x día
Un vaso de leche entera	○	○	○	○	○	○	○	○	○	○
Un vaso de leche descremada o light	○	○	○	○	○	○	○	○	○	○
Un vaso de leche semidescremada	○	○	○	○	○	○	○	○	○	○
Una cucharada de queso crema	○	○	○	○	○	○	○	○	○	○
Una rebanada de queso Oaxaca	○	○	○	○	○	○	○	○	○	○
Una rebanada de queso fresco	○	○	○	○	○	○	○	○	○	○
Un helado de leche con barquillo	○	○	○	○	○	○	○	○	○	○
Un helado de leche sin barquillo	○	○	○	○	○	○	○	○	○	○
Una taza de yogurt	○	○	○	○	○	○	○	○	○	○
Productos lácteos fermentados (yakult, soful, etc.)	○	○	○	○	○	○	○	○	○	○
Margarina que agregue al pan (una untada)	○	○	○	○	○	○	○	○	○	○
Mantequilla que agregue al pan (una untada)	○	○	○	○	○	○	○	○	○	○
Otro: ____________ (Especifique)	○	○	○	○	○	○	○	○	○	○

24. NO RELLENAR
⓪ ① ② ③ ④ ⑤ ⑥ ⑦ ⑧ ⑨

Nota: Cuando come huevo, si es que come dos en cada ocasión, calculé el número total de huevos en promedio

25.- Huevo, Carnes y Embutidos	Nunca	Menos de 1 vez x mes	1 - 3 x sem.	1 x sem.	2 - 4 x sem.	5 - 6 x sem.	1 x día	2 - 3 x día	4 - 5 x día	6 ó más x día
Un huevo	○	○	○	○	○	○	○	○	○	○
Una pieza de pollo	○	○	○	○	○	○	○	○	○	○
Una rebanada de tocino	○	○	○	○	○	○	○	○	○	○
Una salchicha	○	○	○	○	○	○	○	○	○	○
Una rebanada de jamón	○	○	○	○	○	○	○	○	○	○
Una rebanada de jamón de pavo	○	○	○	○	○	○	○	○	○	○
Un bistec de hígado o hígado de pollo	○	○	○	○	○	○	○	○	○	○
Una porción de chorizo o longaniza	○	○	○	○	○	○	○	○	○	○
Un platillo con carne de puerco	○	○	○	○	○	○	○	○	○	○
Un platillo de cecina de res o de puerco	○	○	○	○	○	○	○	○	○	○
Un platillo con atún (en lata)	○	○	○	○	○	○	○	○	○	○
Un platillo con sardina (en lata)	○	○	○	○	○	○	○	○	○	○
Una porción de pescado fresco (huachinango, róbalo, mojarra, salmón, etc.)	○	○	○	○	○	○	○	○	○	○
Una porción de pulpos / calamar / camarón	○	○	○	○	○	○	○	○	○	○
Un pedazo de chicharrón	○	○	○	○	○	○	○	○	○	○
Un plato de barbacoa	○	○	○	○	○	○	○	○	○	○
Una porción de pescado seco (bacalao, charales, etc.)	○	○	○	○	○	○	○	○	○	○

26.- ¿Consume margarina comúnmente?

○ Sí ○ No

Especifique qué marca usa

26) NO RELLENAR
⓪ ① ② ③ ④ ⑤ ⑥ ⑦ ⑧ ⑨
⓪ ① ② ③ ④ ⑤ ⑥ ⑦ ⑧ ⑨

28.- ¿Cuántas veces por semana come en su casa la comida principal?

○ Nunca ○ 3 a 4 veces
○ 1 a 2 veces ○ 5 a 7 veces

27.- ¿Qué tipo de grasa utiliza normalmente para cocinar?

La que más usa es:

○ Ninguna ○ Mantequilla
○ Margarina ○ Manteca de puerco
○ Aceite vegetal ○ Manteca vegetal

Especifique qué marca usa ____________

27a) NO RELLENAR
⓪ ① ② ③ ④ ⑤ ⑥ ⑦ ⑧ ⑨
⓪ ① ② ③ ④ ⑤ ⑥ ⑦ ⑧ ⑨

En segundo lugar usa:

○ Ninguna ○ Mantequilla
○ Margarina ○ Manteca de puerco
○ Aceite vegetal ○ Manteca vegetal

Especifique qué marca usa ____________

27b) NO RELLENAR
⓪ ① ② ③ ④ ⑤ ⑥ ⑦ ⑧ ⑨
⓪ ① ② ③ ④ ⑤ ⑥ ⑦ ⑧ ⑨

29.- ¿Cuántas veces por semana cena en su casa? ○ Nunca ○ 1 a 2 veces ○ 3 a 4 veces ○ 5 ó más

30.- ¿Cuántas veces por semana come alimentos fritos fuera de su casa? (Ej. papas fritas, pollo o pescado frito) ○ Nunca ○ 1 a 2 veces ○ 3 a 4 veces ○ 5 ó más

31.- ¿Cuántas veces por semana come alimentos fritos en su casa? (Ej. papas fritas, pollo o pescado frito) ○ Nunca ○ 1 a 2 veces ○ 3 a 4 veces ○ 5 ó más

Marque el círculo que indica la frecuencia con que comió cada uno de los siguientes alimentos durante los últimos 12 meses. Considere que aún cuando hay frutas que sólo consume en temporada, marque el promedio que consumió durante esa temporada específicamente.

**32.- Frutas**	Nunca	Menos de 1 vez x mes	1 - 3 x mes	1 x sem.	2 - 4 x sem.	5 - 6 x sem.	1 x día	2 - 3 x día	4 - 5 x día	6 ó más x día
Un plátano	○	○	○	○	○	○	○	○	○	○
Media taza de ciruelas	○	○	○	○	○	○	○	○	○	○
Un durazno	○	○	○	○	○	○	○	○	○	○
Una manzana	○	○	○	○	○	○	○	○	○	○
Una naranja	○	○	○	○	○	○	○	○	○	○
Un vaso de jugo de naranja	○	○	○	○	○	○	○	○	○	○
Media taza de uvas	○	○	○	○	○	○	○	○	○	○
Media taza de fresas	○	○	○	○	○	○	○	○	○	○
Una rebanada de melón	○	○	○	○	○	○	○	○	○	○
Una rebanada de sandía	○	○	○	○	○	○	○	○	○	○
Un mango	○	○	○	○	○	○	○	○	○	○
Una mandarina	○	○	○	○	○	○	○	○	○	○
Una pera	○	○	○	○	○	○	○	○	○	○
Una rebanada de mamey	○	○	○	○	○	○	○	○	○	○
Una tuna	○	○	○	○	○	○	○	○	○	○
Un zapote	○	○	○	○	○	○	○	○	○	○
Una rebanada de papaya	○	○	○	○	○	○	○	○	○	○
Una rebanada de piña	○	○	○	○	○	○	○	○	○	○
Una guayaba	○	○	○	○	○	○	○	○	○	○
Media taza de pasitas	○	○	○	○	○	○	○	○	○	○
Media taza de cacahuates	○	○	○	○	○	○	○	○	○	○
Un cuarto de taza de nueces	○	○	○	○	○	○	○	○	○	○
Un cuarto de taza de almendras	○	○	○	○	○	○	○	○	○	○
Un vaso de jugo de frutas frescas	○	○	○	○	○	○	○	○	○	○

**33.- Verduras**	Nunca	Menos de 1 vez x mes	1 - 3 x mes	1 x sem.	2 - 4 x sem.	5 - 6 x sem.	1 x día	2 - 3 x día	4 - 5 x día	6 ó más x día
Media taza de coliflor	○	○	○	○	○	○	○	○	○	○
Media taza de espinacas	○	○	○	○	○	○	○	○	○	○
Media taza de calabacitas o chayote	○	○	○	○	○	○	○	○	○	○
Una hoja de lechuga	○	○	○	○	○	○	○	○	○	○
Un jitomate en salsa o guisado	○	○	○	○	○	○	○	○	○	○
Un jitomate crudo o en ensalada	○	○	○	○	○	○	○	○	○	○
Un nopal	○	○	○	○	○	○	○	○	○	○
Medio aguacate	○	○	○	○	○	○	○	○	○	○
Media taza de flor de calabaza	○	○	○	○	○	○	○	○	○	○
Un betabel	○	○	○	○	○	○	○	○	○	○
Una rebanada de cebolla cruda o cocida	○	○	○	○	○	○	○	○	○	○
Media taza de ejotes	○	○	○	○	○	○	○	○	○	○
Media taza de chícharos	○	○	○	○	○	○	○	○	○	○
Un plato de sopa de verdura	○	○	○	○	○	○	○	○	○	○
Una cucharada de salsa picante	○	○	○	○	○	○	○	○	○	○
Una cucharada de chiles de lata	○	○	○	○	○	○	○	○	○	○
Un platillo con chile seco	○	○	○	○	○	○	○	○	○	○

39a) NO RELLENAR
⓪ ① ② ③ ④ ⑤ ⑥ ⑦ ⑧ ⑨
⓪ ① ② ③ ④ ⑤ ⑥ ⑦ ⑧ ⑨

39b) NO RELLENAR
⓪ ① ② ③ ④ ⑤ ⑥ ⑦ ⑧ ⑨
⓪ ① ② ③ ④ ⑤ ⑥ ⑦ ⑧ ⑨

39c) NO RELLENAR
⓪ ① ② ③ ④ ⑤ ⑥ ⑦ ⑧ ⑨
⓪ ① ② ③ ④ ⑤ ⑥ ⑦ ⑧ ⑨

34.- ¿Cuántas cucharadas de azúcar agrega a sus comidas o bebidas en un día? (en total)

⓪ ① ② ③ ④ ⑤ ⑥ ⑦ ⑧ ⑨

35.- ¿En su casa ponen el salero en la mesa durante las comidas?

○ Sí ○ No

36.- ¿Agrega sal a sus alimentos antes de probarlos?

○ Sí ○ No

¿Cuántas veces por semana come hamburguesas o pizza fuera de su casa?

○ Nunca ○ Una ó 2 veces ○ 3 a 4 veces ○ 5 ó más

37.- ¿Consume regularmente Canderel, Splenda o Nutrasuit?

○ Sí ○ No

¿Desde cuándo los consume?

○ Menos de 1 año ○ 3 a 5 años
○ 1 a 2 años ○ Más de 5 años

¿Con qué frecuencia los consume?

○ 1 a 3 al mes ○ 5 a 6 por semana ○ 4 a 5 por día
○ 1 por semana ○ 1 por día ○ 6 ó más por día
○ 2 a 4 por semana ○ 2 a 3 por día

Marque el círculo que indica la frecuencia con que comió cada uno de los siguientes alimentos durante los **últimos 12 meses**.
Nota: Cuando come tortilla, pan de dulce o galletas, si es que come más de uno(a) calcule el número total de éstos en promedio.

**38.- Cereales, Leguminosas y Harinas**	**Nunca**	**Menos de 1 vez x mes**	**1 - 3 x mes**	**1 x sem.**	**2 - 4 x sem.**	**5 - 6 x sem.**	**1 x día**	**2 - 3 x día**	**4 - 5 x día**	**6 ó más x día**
Una rebanada de pastel	○	○	○	○	○	○	○	○	○	○
Una pieza de pan dulce	○	○	○	○	○	○	○	○	○	○
Una galleta dulce	○	○	○	○	○	○	○	○	○	○
Una tablilla de chocolate	○	○	○	○	○	○	○	○	○	○
Una cucharada de mermelada, miel o ate	○	○	○	○	○	○	○	○	○	○
Una tortilla de maíz	○	○	○	○	○	○	○	○	○	○
Una tortilla de harina	○	○	○	○	○	○	○	○	○	○
Un bolillo	○	○	○	○	○	○	○	○	○	○
Una rebanada de pan blanco de caja (ej. Bimbo Blanco)	○	○	○	○	○	○	○	○	○	○
Una rebanada de pan integral de caja (ej. Bimbo, etc.)	○	○	○	○	○	○	○	○	○	○
Una galleta salada	○	○	○	○	○	○	○	○	○	○
Un plato de arroz	○	○	○	○	○	○	○	○	○	○
Un plato de avena	○	○	○	○	○	○	○	○	○	○
Un plato de sopa de pasta	○	○	○	○	○	○	○	○	○	○
Una bolsita de churritos, papas, frituras, etc.	○	○	○	○	○	○	○	○	○	○
Un taco al pastor	○	○	○	○	○	○	○	○	○	○
Una memela, quesadilla o sope	○	○	○	○	○	○	○	○	○	○
Un plato de pozole	○	○	○	○	○	○	○	○	○	○
Un plato de habas verdes	○	○	○	○	○	○	○	○	○	○
Un plato de lentejas	○	○	○	○	○	○	○	○	○	○
Un plato de frijoles	○	○	○	○	○	○	○	○	○	○
Una cucharada de salvado de trigo	○	○	○	○	○	○	○	○	○	○
Un elote	○	○	○	○	○	○	○	○	○	○
Una papa	○	○	○	○	○	○	○	○	○	○
Una taza de cereal (Especifique)	○	○	○	○	○	○	○	○	○	○

En primer lugar:

Especifique qué marca usa

38a) NO RELLENAR
⓪ ① ② ③ ④ ⑤ ⑥ ⑦ ⑧ ⑨
⓪ ① ② ③ ④ ⑤ ⑥ ⑦ ⑧ ⑨

En segundo lugar:

Especifique qué marca usa

38b) NO RELLENAR
⓪ ① ② ③ ④ ⑤ ⑥ ⑦ ⑧ ⑨
⓪ ① ② ③ ④ ⑤ ⑥ ⑦ ⑧ ⑨

	**Nunca**	**Menos de 1 vez x mes**	**1 - 3 x sem.**	**1 x sem.**	**2 - 4 x sem.**	**5 - 6 x sem.**	**1 x día**	**2 - 3 x día**	**4 - 5 x día**	**6 ó más x día**
Una barra de cereal (Especifique)	○	○	○	○	○	○	○	○	○	○

En primer lugar:

Especifique qué marca usa

38c) NO RELLENAR
⓪ ① ② ③ ④ ⑤ ⑥ ⑦ ⑧ ⑨
⓪ ① ② ③ ④ ⑤ ⑥ ⑦ ⑧ ⑨

En segundo lugar:

Especifique qué marca usa

38d) NO RELLENAR
⓪ ① ② ③ ④ ⑤ ⑥ ⑦ ⑧ ⑨
⓪ ① ② ③ ④ ⑤ ⑥ ⑦ ⑧ ⑨

Nota: Considere la medición de estas bebidas como una botella mediana, una lata o un vaso.

**39.- Bebidas**	Nunca	Menos de 1 vez x mes	1 - 3 x mes	1 x sem.	2 - 4 x sem.	5 - 6 x sem.	1 x día	2 - 3 x día	4 - 5 x día	6 ó más x día
Un refresco embotellado de cola	◯	◯	◯	◯	◯	◯	◯	◯	◯	◯
Un refresco embotellado de sabor	◯	◯	◯	◯	◯	◯	◯	◯	◯	◯
Un refresco embotellado dietético	◯	◯	◯	◯	◯	◯	◯	◯	◯	◯
Un vaso de agua de sabor (fruta natural)	◯	◯	◯	◯	◯	◯	◯	◯	◯	◯
Un vaso de agua de sabor (en polvo preparada)	◯	◯	◯	◯	◯	◯	◯	◯	◯	◯
Un vaso de agua de sabor (dietética)	◯	◯	◯	◯	◯	◯	◯	◯	◯	◯
Un vaso de jugo industrializado	◯	◯	◯	◯	◯	◯	◯	◯	◯	◯
Una taza o botella de té	◯	◯	◯	◯	◯	◯	◯	◯	◯	◯
Otra: (Especifique) ____________________	◯	◯	◯	◯	◯	◯	◯	◯	◯	◯
Una taza de café con leche	◯	◯	◯	◯	◯	◯	◯	◯	◯	◯
Una taza de café sin leche	◯	◯	◯	◯	◯	◯	◯	◯	◯	◯
Una taza de atole con leche	◯	◯	◯	◯	◯	◯	◯	◯	◯	◯
Una taza de atole sin leche	◯	◯	◯	◯	◯	◯	◯	◯	◯	◯
Una taza de chocolate con leche	◯	◯	◯	◯	◯	◯	◯	◯	◯	◯
Una taza de chocolate sin leche	◯	◯	◯	◯	◯	◯	◯	◯	◯	◯
Otra: (Especifique) ____________________	◯	◯	◯	◯	◯	◯	◯	◯	◯	◯
Una copa de vino	◯	◯	◯	◯	◯	◯	◯	◯	◯	◯
Una cerveza	◯	◯	◯	◯	◯	◯	◯	◯	◯	◯
Una copa de brandy	◯	◯	◯	◯	◯	◯	◯	◯	◯	◯
Una copa de whisky	◯	◯	◯	◯	◯	◯	◯	◯	◯	◯
Una copa de tequila	◯	◯	◯	◯	◯	◯	◯	◯	◯	◯
Una copa de ron	◯	◯	◯	◯	◯	◯	◯	◯	◯	◯
Una copa de aguardiente	◯	◯	◯	◯	◯	◯	◯	◯	◯	◯
Un vaso de pulque	◯	◯	◯	◯	◯	◯	◯	◯	◯	◯
Otra: (Especifique) ____________________	◯	◯	◯	◯	◯	◯	◯	◯	◯	◯

40.- ¿La última vez que tomó algún tipo de bebida alcohólica, cuántas copas se tomó?

- ◯ Nunca ha tomado una bebida alcohólica
- ◯ Una copa
- ◯ Dos copas
- ◯ Tres copas
- ◯ Cuatro copas
- ◯ Cinco copas
- ◯ Seis copas
- ◯ Siete copas
- ◯ Ocho a 10 copas
- ◯ 11 a 15 copas
- ◯ Más de 15 copas

41.- ¿Actualmente utiliza suplementos de fibra en su dieta?

◯ Sí ◯ No

41) NO RELLENAR ⓪ ① ② ③ ④ ⑤ ⑥ ⑦ ⑧ ⑨

De la siguiente lista, seleccione cuáles medicamentos consume: en la segunda columna, marque la cantidad de cucharadas que consume en un día. Marque también cuál es la presentación del medicamento, rellenando el círculo que corresponda a la presentación de ese medicamento. Por ejemplo, si ingiere en un día 3 cucharadas de metamucil sabor natural, anote un 3 en la columna de cantidad y rellene el círculo de la columna de polvo natural (sin sabor) o polvo de sabor, según sea el caso.

Nombre del Medicamento	Cantidad	Presentación: Polvo natural (sin sabor)	Presentación: Polvo de sabor
Metamucil	⓪ ① ② ③ ④ ⑤ ⑥ ⑦ ⑧ ⑨	◯	◯
Psilumax	⓪ ① ② ③ ④ ⑤ ⑥ ⑦ ⑧ ⑨	◯	◯
Otro (Especifique) ____________	⓪ ① ② ③ ④ ⑤ ⑥ ⑦ ⑧ ⑨	◯	◯

48.- ¿Cuántas “porciones” de cereales como: arroz, tortilla, pastas, pan, etc., debe consumir diariamente?

◯ De 1 a 5 ◯ De 6 a 10 ◯ De 11 a 15 ◯ No sé

En caso de que requiramos localizarlo(a), sería tan amable de proporcionarnos algunos datos para contactarlo(a):
Recuerde que estos datos son confidenciales.

Tel.: ______________________________

Cel.: ______________________________

E-mail: ______________________________

42.- ¿Cuántos litros de agua simple consume al día?

◯ Menos de 1/2 litro ◯ 1 Litro ◯ 3 Litros
◯ 1/2 Litro ◯ 2 Litros

43.- ¿Qué cantidad de líquidos consume al día diferentes al agua (un vaso mediano contiene 250 ml.)**?**

◯ Menos de 1/2 litro ◯ 1 Litro ◯ 3 Litros
◯ 1/2 Litro ◯ 2 Litros

44.- ¿Con qué frecuencia lee la información nutricional que se encuentra al reverso de las envolturas de los alimentos?

◯ Siempre ◯ Casi siempre ◯ Rara vez ◯ Nunca

45.- ¿Comprende la información nutricional que se encuentra al reverso de las envolturas de los alimentos?

◯ Totalmente ◯ Parcialmente ◯ Casi nada ◯ Nada

46.- ¿Cuántas “porciones” de fruta debe comer diariamente?

◯ De 1 a 2 ◯ De 2 a 4 ◯ De 4 a 6 ◯ No sé

47.- ¿Cuántas “porciones” de verdura debe comer diariamente?

◯ De 1 a 2 ◯ De 3 a 5 ◯ De 6 a 8 ◯ No sé

49.- ¿Cuántas "porciones" de lácteos como: queso, yogurt o leche, debe consumir diariamente?

De 1 a 3	De 4 a 6	De 7 a 9	No sé
◯	◯	◯	◯

50.- ¿Cuántas "porciones" de grasa vegetal como: aceite de olivo, de canola, aderezos, aguacate, etc., debe consumir diariamente?

1 a 2	3 a 4	De 5 a 6	No sé
◯	◯	◯	◯

51.- ¿Cuántas "porciones" de alimentos dulces como: mermelada, dulces, chocolates, refrescos, cajeta, etc., debe consumir al día?

0 a 1	2 a 3	4 a 5	No sé
◯	◯	◯	◯

52.- ¿Cuántas "porciones" de alimentos de origen animal como: carnes, pescado, queso y huevo, debe consumir diariamente?

De 1 a 2	De 3 a 4	De 5 a 6	No sé
◯	◯	◯	◯

55.- ¿Qué tan saludable considera usted la comida del lugar donde generalmente come? Elija solamente una opción

Muy Saludable	Saludable	Poco Saludable	Nada Saludable
◯	◯	◯	◯

53.- Generalmente, ¿dónde obtiene usted información que le pueda ayudar a tener una alimentación saludable?
Elija solamente una opción

- ◯ De libros o revistas
- ◯ De clínicas o consultorios
- ◯ De la familia
- ◯ En el trabajo o la escuela
- ◯ De la radio
- ◯ De la televisión
- ◯ No tengo información
- ◯ Otra fuente ¿Cuál? ____________________
  (Especifique)

53) NO RELLENAR ⓪ ① ② ③ ④ ⑤ ⑥ ⑦ ⑧ ⑨

54) NO RELLENAR ⓪ ① ② ③ ④ ⑤ ⑥ ⑦ ⑧ ⑨

54.- Generalmente, durante la semana o en sus días de trabajo, ¿dónde realiza usted sus 3 comidas? Elija solamente una opción

	Desayuno	Comida	Cena
En casa	◯	◯	◯
En restaurantes o fondas	◯	◯	◯
En puestos ambulantes	◯	◯	◯
En el comedor de mi trabajo	◯	◯	◯
Llevo mis alimentos	◯	◯	◯
Consumo alimentos de máquinas distribuidoras de comida rápida	◯	◯	◯

Otro: ¿Cuál? ____________________
(Especifique)

56.- Cuando usted come fuera de su casa ¿Cómo considera los siguientes aspectos del lugar donde generalmente come?
Responda cada una de las siguientes preguntas

	Muy Adecuado	Adecuado	Poco Adecuado	Nada Adecuado
El costo de los alimentos	◯	◯	◯	◯
La higiene	◯	◯	◯	◯
La distancia y facilidad para llegar al lugar	◯	◯	◯	◯
El sabor y presentación de los alimentos	◯	◯	◯	◯
	**Demasiada**	**Adecuada**	**Insuficiente**	**No sé**
La cantidad de verduras es:	◯	◯	◯	◯
La cantidad de frutas es:	◯	◯	◯	◯
La presencia de carne de cerdo, res o pollo es:	◯	◯	◯	◯
La presencia de carne de pescado o mariscos es:	◯	◯	◯	◯
La cantidad de sal en los platillos es:	◯	◯	◯	◯
La cantidad de grasa en los platillos es:	◯	◯	◯	◯
La presencia de postres, bebidas azucaradas, refrescos y pastelillos es:	◯	◯	◯	◯

57.- Para favorecer una alimentación saludable en las personas que tienen que comer fuera de su casa. ¿Cuál considera usted que sería la mejor propuesta?

- ◯ Reglamentar que los vendedores ambulantes ofrezcan opciones de alimentos y bebidas saludables.
- ◯ Reglamentar que las fondas y restaurantes ofrezcan opciones de alimentos y bebidas saludables.
- ◯ Mejorar la calidad de los alimentos que se ofrecen en los comedores de los centros de trabajo.
- ◯ Que no se permita la venta de alimentos y bebidas por vendedores ambulantes cerca de los centros de trabajo o escuelas.
- ◯ Reducir el número de máquinas distribuidoras de comida rápida y de bebidas azucaradas.
- ◯ Reglamentar que las máquinas distribuidoras de comida rápida y bebidas ofrezcan opciones saludables.
- ◯ Otra ¿Cuál? ____________________

57) NO RELLENAR ⓪ ① ② ③ ④ ⑤ ⑥ ⑦ ⑧ ⑨

58.- Para llevar a cabo una alimentación saludable, ¿qué tanta importancia tienen para usted los siguientes elementos?

- ◯ Fuerza de voluntad y disciplina
- ◯ Educación alimentaria
- ◯ Apoyo familiar para consumir alimentos saludables en casa
- ◯ El tener opciones donde se vendan alimentos saludables, cuando uno está fuera de su casa.
- ◯ Tiempo para preparar alimentos saludables
- ◯ Dinero para comprar alimentos saludables
- ◯ Apoyo de amigos y/o compañeros para consumir alimentos saludables en el trabajo, escuela, etc.
- ◯ Otra ¿Cuál? ____________________

58) NO RELLENAR ⓪ ① ② ③ ④ ⑤ ⑥ ⑦ ⑧ ⑨

59.- ¿Cuando usted come, generalmente qué hace? Por favor responda en cada una de las siguientes preguntas:

a) ¿Usted toma el tiempo necesario para terminar de comer?
- ◯ Nunca
- ◯ Casi siempre
- ◯ Casi nunca
- ◯ Siempre

b) ¿Qué tan rápido come sus alimentos?
- ◯ Muy rápido
- ◯ Despacio
- ◯ Rápido
- ◯ Muy despacio

c) ¿Se distrae al comer, ya sea viendo la televisión, trabajando, leyendo, platicando o resolviendo problemas cotidianos?
- ◯ Nunca
- ◯ Casi siempre
- ◯ Casi nunca
- ◯ Siempre

d) ¿Disfruta el momento de comer?
- ◯ Lo disfruto bastante
- ◯ Casi no lo disfruto
- ◯ Lo disfruto
- ◯ No lo disfruto nada

e) ¿Selecciona el tipo de alimentos que va a consumir en favor de su salud?
- ◯ Nunca
- ◯ Casi siempre
- ◯ Casi nunca
- ◯ Siempre

f) ¿Selecciona la cantidad de alimentos que va a consumir?
- ◯ Nunca
- ◯ Casi siempre
- ◯ Casi nunca
- ◯ Siempre

g) ¿Usted come todo lo que le sirven sin dejar comida en el plato?
- ◯ Nunca
- ◯ Casi siempre
- ◯ Casi nunca
- ◯ Siempre

h) Semanalmente ¿Cuántas veces come la comida principal en su casa?
- ◯ De 0 a 1 vez
- ◯ De 4 a 5 veces
- ◯ De 2 a 3 veces
- ◯ De 6 a 7 veces

i) ¿Usted come acompañado de amigos, familiares o colegas?
- ◯ Nunca
- ◯ Casi siempre
- ◯ Casi nunca
- ◯ Siempre

j) ¿Se "salta" usted alguna de las comidas del día?
- ◯ Nunca
- ◯ Casi siempre
- ◯ Casi nunca
- ◯ Siempre

Sección Actividad Física

Esta sección del cuestionario está diseñada para conocer las actividades diarias que usted realiza en su trabajo, en su casa y en su tiempo libre.
La actividad física es parte de nuestro estilo de vida y puede modificarse a través del tiempo, por lo que esta sección del cuestionario será de utilidad para registrar en cada etapa de la Cohorte las actividades que usted realiza. Registrar el cambio o consistencia en el patrón de actividad es importante para nosotros, pues nos permite cumplir con los objetivos del proyecto. Una de las actividades principales es utilizar esta información como base para poder realizar recomendaciones de actividad física a nivel poblacional que permitan mejorar nuestros estilos de vida y mantener un buen estado de salud.

60.- ¿Para que usted pueda hacer ejercicio de manera cotidiana qué necesita? Elija solamente una opción
- ◯ Instalaciones apropiadas
- ◯ Que las instalaciones deportivas estén cerca de mi casa
- ◯ Tiempo para hacer ejercicio
- ◯ Espacios recreativos o áreas verdes en buen estado
- ◯ Dinero para pagar los servicios deportivos
- ◯ Participar en torneos deportivos de: fútbol, beisbol, básquetbol, voleibol, tenis, etc.
- ◯ Instructores capacitados
- ◯ La compañía de amigos, familiares o colegas
- ◯ Otro: ¿Cuál? ______________________

60) NO RELLENAR ⓪ ① ② ③ ④ ⑤ ⑥ ⑦ ⑧ ⑨

61.- ¿Existen instalaciones deportivas cerca de su casa, en su trabajo, o cerca de su trabajo?
Señale las opciones que considere necesarias

	Cerca de mi casa	En mi trabajo	Cerca de mi trabajo
Canchas de voleibol, básquetbol o tenis	◯	◯	◯
Deportivo	◯	◯	◯
Gimnasio	◯	◯	◯
Alberca	◯	◯	◯
Canchas de fútbol o béisbol	◯	◯	◯
Instalaciones para artes marciales	◯	◯	◯
Instalaciones para danza y actividades culturales	◯	◯	◯
Ciclopista o pista de patinaje	◯	◯	◯
Sí hay, pero no sé cuáles son	◯	◯	◯
Otra: ¿Cuál? __________	◯	◯	◯

62.- ¿Cuáles considera usted que son las principales barreras para practicar algún deporte de manera cotidiana?
Puede elegir una o varias opciones.
- ◯ Tener pocas instalaciones deportivas cercanas a mi entorno: centros deportivos, canchas o parques
- ◯ Falta de compañía de amigos, compañeros o familiares
- ◯ Falta de dinero para pagar los servicios deportivos
- ◯ Falta de tiempo
- ◯ Falta de gusto por practicar un deporte
- ◯ Otra ¿Cuál? ______________________

62) NO RELLENAR ⓪ ① ② ③ ④ ⑤ ⑥ ⑦ ⑧ ⑨

64.- ¿Cuáles de las siguientes medidas considera usted, son importantes en la prevención de enfermedades crónicas como diabetes, hipertensión, cáncer o infarto al corazón?
Elija solamente una opción
- ◯ Hacer ejercicio
- ◯ Mantener un peso saludable
- ◯ Mejorar la calidad de la alimentación
- ◯ Acudir con frecuencia al médico
- ◯ Otro: ¿Cuál? ______________________

64) NO RELLENAR ⓪ ① ② ③ ④ ⑤ ⑥ ⑦ ⑧ ⑨

63.- ¿Qué es lo que le motiva o motivaría para hacer deporte?
Puede elegir varias opciones.
- ◯ El sentirme bien físicamente
- ◯ El sentirme bien mentalmente
- ◯ El mantener mi figura
- ◯ Los beneficios para la salud
- ◯ Otra ¿Cuál? ______________________

61) NO RELLENAR ⓪ ① ② ③ ④ ⑤ ⑥ ⑦ ⑧ ⑨

63) NO RELLENAR ⓪ ① ② ③ ④ ⑤ ⑥ ⑦ ⑧ ⑨

65.- Usted considera que su riesgo a padecer enfermedades crónicas como diabetes, hipertensión, cáncer o infarto al corazón? es: (Si ya padece alguna de estas enfermedades, pase a la siguiente pregunta)
- ◯ Muy elevado
- ◯ Bajo
- ◯ Elevado
- ◯ Muy bajo

66.- Si la institución donde usted trabaja, el gobierno o la iniciativa privada le ofrecieran un programa de actividad física para prevenir enfermedades crónicas y obesidad, ¿Cuál elegiría? Elija solamente una opción.

○ **Programa 1.**
“*Actividad Física en el centro de trabajo*“ que incluya:

a. Espacios adecuados
b. Equipo y material necesario
c. Organización de torneos y actividades recreativas durante todo el año
d. Regaderas
e. Que la institución de trabajo proporcione ½ hora de la jornada laboral, y el trabajador también aporte ½ hora de su tiempo
f. Que la actividad física sea realizada entre 3 a 5 días a la semana
g. Que el trabajador aporte un porcentaje del costo total de operación del programa

○ **Programa 2.**
“*Horarios flexibles para practicar un deporte*“ que incluya:

a. Que la institución de trabajo le dé a usted la oportunidad de adaptar sus horarios para poder realizar ejercicio
b. Que la institución de trabajo considere 1 hora para hacer la actividad física más el tiempo de traslado
c. Que la actividad física sea realizada entre 3 a 5 días a la semana

○ **Programa 3.**
“*Sistema de transporte en bicicleta*“ que incluya:

a. Ciclopista, con carriles adaptados, señalizados y seguros para transportarse en bicicletas rentadas dentro de la ciudad
b. Contar con estaciones de bicicletas disponibles en diferentes puntos de la ciudad
c. Que los costos de la ciclopista y las bicicletas sean a cargo de la iniciativa privada
d. Que el gobierno federal, estatal y/o municipal subsidie una parte del costo para los usuarios

67.- De las siguientes actividades marque aquella(s) que haya realizado en su tiempo libre, durante el año pasado seleccionando el círculo que mejor indica los días, el tiempo y la intensidad con que la realiza:

Actividad	¿Qué días las realizó? Lu Ma Mi Ju Vi Sa Do	5-14 min x semana	15-30 min x semana	31-60 min x semana	1-2 hrs x semana	3-4 hrs x semana	5-6 hrs x semana	Más de 6 hrs x semana	Cada actividad la realiza en forma: Ligera	Moderada	Intensa
○ **Ninguna**											
Caminar	○ ○ ○ ○ ○ ○ ○	○	○	○	○	○	○	○	○	○	○
Correr	○ ○ ○ ○ ○ ○ ○	○	○	○	○	○	○	○	○	○	○
Andar en bicicleta	○ ○ ○ ○ ○ ○ ○	○	○	○	○	○	○	○	○	○	○
Softbol/beisbol	○ ○ ○ ○ ○ ○ ○	○	○	○	○	○	○	○	○	○	○
Fútbol soccer	○ ○ ○ ○ ○ ○ ○	○	○	○	○	○	○	○	○	○	○
Voleibol	○ ○ ○ ○ ○ ○ ○	○	○	○	○	○	○	○	○	○	○
Aerobics	○ ○ ○ ○ ○ ○ ○	○	○	○	○	○	○	○	○	○	○
Bailar	○ ○ ○ ○ ○ ○ ○	○	○	○	○	○	○	○	○	○	○
Boliche	○ ○ ○ ○ ○ ○ ○	○	○	○	○	○	○	○	○	○	○
Nadar	○ ○ ○ ○ ○ ○ ○	○	○	○	○	○	○	○	○	○	○
Tenis	○ ○ ○ ○ ○ ○ ○	○	○	○	○	○	○	○	○	○	○
Frontón	○ ○ ○ ○ ○ ○ ○	○	○	○	○	○	○	○	○	○	○
Básquetbol	○ ○ ○ ○ ○ ○ ○	○	○	○	○	○	○	○	○	○	○
Squash	○ ○ ○ ○ ○ ○ ○	○	○	○	○	○	○	○	○	○	○
Otro:____________ ____________ (Especifique)	○ ○ ○ ○ ○ ○ ○	○	○	○	○	○	○	○	○	○	○

Ligera: como la actividad que se asemeja a caminar en forma tranquila.
Moderada: actividad que realiza con esfuerzo provocando en usted sudoración.
Intensa: actividad que realiza una persona que entrena o se prepara para una competencia deportiva.

67) NO RELLENAR
0 1 2 3 4 5 6 7 8 9
0 1 2 3 4 5 6 7 8 9

68.- Marque el círculo que mejor indica el tiempo acumulado que dedica a las siguientes actividades de la vida diaria durante una semana de rutina (fuera de su jornada de trabajo):

Actividad	Nunca	Menos de 15 minutos	16-29 min x semana	30-59 min x semana	1-2 hrs x semana	3-4 hrs x semana	5-6 hrs x semana	Más de 6 hrs
Cocinar	○	○	○	○	○	○	○	○
Servir comida	○	○	○	○	○	○	○	○
Lavar trastes	○	○	○	○	○	○	○	○
Limpiar ventanas	○	○	○	○	○	○	○	○
Trapear pisos	○	○	○	○	○	○	○	○
Lavar ropa	○	○	○	○	○	○	○	○
Planchar ropa	○	○	○	○	○	○	○	○
Coser ropa o remendar	○	○	○	○	○	○	○	○
Arreglar el jardín	○	○	○	○	○	○	○	○
Ir de compras (al mercado)	○	○	○	○	○	○	○	○
Atender niños (menores de 3 años)	○	○	○	○	○	○	○	○
Atender a un anciano	○	○	○	○	○	○	○	○
Atender a un discapacitado	○	○	○	○	○	○	○	○
Otra:____________________ (Especifique)	○	○	○	○	○	○	○	○

68) NO RELLENAR
0 1 2 3 4 5 6 7 8 9
0 1 2 3 4 5 6 7 8 9

69.- Marque el círculo que mejor indica el tiempo que pasa, en un día de trabajo normal, en actividades como las siguientes
(Nota: No se podrán sumar más horas que las de su jornada)

Actividad de un día de trabajo	Menos de 15 min	15-30 min	31-60 min	1-2 hrs.	3-4 hrs.	5-6 hrs.	Más de 6 hrs.
No trabajo ○ (pase a la siguiente pregunta)							
Estar sentado (a)	○	○	○	○	○	○	○
Estar de pie	○	○	○	○	○	○	○
Estar caminando	○	○	○	○	○	○	○
Caminar levantando o empujando objetos de 5-10 Kgs.	○	○	○	○	○	○	○
Caminar levantando o empujando más de 10 Kgs.	○	○	○	○	○	○	○
Subir escaleras	○	○	○	○	○	○	○
Trabajar con herramienta ligera	○	○	○	○	○	○	○
Trabajar con herramienta pesada	○	○	○	○	○	○	○
Otro: (Especifique) ______________________	○	○	○	○	○	○	○

70.- Marque el círculo que mejor indica el tiempo que dedica, fuera de su jornada de trabajo, a las siguientes actividades:

Actividades	Tiempo acumulado en lunes a viernes							Tiempo acumulado en sábado y domingo						
	Menos de 15 min	16-29 min	30-59 min	1-2 hrs.	3-4 hrs.	5-6 hrs.	Más de 6 hrs.	Menos de 15 min	16-29 min	30-59 min	1-2 hrs.	3-4 hrs.	5-6 hrs.	Más de 6 hrs.
Leer (periódico, revistas, libro)	○	○	○	○	○	○	○	○	○	○	○	○	○	○
Escribir	○	○	○	○	○	○	○	○	○	○	○	○	○	○
Usar la computadora	○	○	○	○	○	○	○	○	○	○	○	○	○	○
Mirar televisión	○	○	○	○	○	○	○	○	○	○	○	○	○	○
Sentarse a escuchar radio o música	○	○	○	○	○	○	○	○	○	○	○	○	○	○
Ir al cine / teatro / concierto	○	○	○	○	○	○	○	○	○	○	○	○	○	○
Tomar una siesta	○	○	○	○	○	○	○	○	○	○	○	○	○	○
Juegos de mesa	○	○	○	○	○	○	○	○	○	○	○	○	○	○
Planear actividades	○	○	○	○	○	○	○	○	○	○	○	○	○	○
Otra: (Especifique)__________________	○	○	○	○	○	○	○	○	○	○	○	○	○	○
Otra: (Especifique)__________________	○	○	○	○	○	○	○	○	○	○	○	○	○	○

69) NO RELLENAR
⓪ ① ② ③ ④ ⑤ ⑥ ⑦ ⑧ ⑨
⓪ ① ② ③ ④ ⑤ ⑥ ⑦ ⑧ ⑨

70a) NO RELLENAR
⓪ ① ② ③ ④ ⑤ ⑥ ⑦ ⑧ ⑨
⓪ ① ② ③ ④ ⑤ ⑥ ⑦ ⑧ ⑨

70b) NO RELLENAR
⓪ ① ② ③ ④ ⑤ ⑥ ⑦ ⑧ ⑨
⓪ ① ② ③ ④ ⑤ ⑥ ⑦ ⑧ ⑨

71.- ¿Qué promedio de horas duerme al día?

De lunes a viernes |__|__|

○	○	○	○	○	○	○	○	○	○	○	○	○	○	○	○
1	2	3	4	5	6	7	8	9	10	11	12	13	14	1 5	16

En sábados y domingos |__|__|

○	○	○	○	○	○	○	○	○	○	○	○	○	○	○	○
1	2	3	4	5	6	7	8	9	10	11	12	13	14	1 5	16

72.- ¿Cuántos trabajos tiene?

① ② ③ ⓪ → (pase a la siguiente pregunta)

¿Cuántas horas al día labora en su(s) trabajo(s)?

Trabajo 1	④	⑥	⑧	⑩	⑫	⑭	⑯
Trabajo 2	④	⑥	⑧	⑩	⑫	⑭	⑯
Trabajo 3	④	⑥	⑧	⑩	⑫	⑭	⑯

¿Qué turno(s) labora?

	Mat.	Vesp.	Noct.	Jornada Acumulada
Trabajo 1	○	○	○	○
Trabajo 2	○	○	○	○
Trabajo 3	○	○	○	○

73.- Nos gustaría saber cuánto tiempo hacía actividad física en su tiempo libre en el pasado. Por favor rellene el círculo que mejor indica el tiempo que dedicaba a estas actividades en las distintas edades indicadas.

	Tiempo de actividad						
Edad	Menos de 15 min	15-30 min	31-60 min	1-2 hrs./sem	3-4 hrs./sem	5-6 hrs./sem	Más de 6 hrs/sem
A los 10 años	○	○	○	○	○	○	○
A los 15 años	○	○	○	○	○	○	○
A los 20 años	○	○	○	○	○	○	○
A los 25 años	○	○	○	○	○	○	○
A los 30 años	○	○	○	○	○	○	○
A los 35 años	○	○	○	○	○	○	○
A los 40 años	○	○	○	○	○	○	○
A los 45 años	○	○	○	○	○	○	○
A los 50 años	○	○	○	○	○	○	○
A los 55 años	○	○	○	○	○	○	○
A los 60 años	○	○	○	○	○	○	○
A los 65 años	○	○	○	○	○	○	○
Actualmente	○	○	○	○	○	○	○

74.- ¿Cuánto tiempo le toma llegar a su trabajo? (Las siguientes preguntas se refieren únicamente al trabajo 1)

- ◯ No trabajo (pase a la siguiente pregunta)
- ◯ 5 a 14 min.
- ◯ 15 a 29 min.
- ◯ 30 a 44 min.
- ◯ 45 a 1 hora
- ◯ Más de 1 hora

¿En el trayecto de su casa al trabajo, cuántas cuadras camina?

|_|_| cuadras

⓪ ①
⓪ ① ② ③ ④ ⑤ ⑥ ⑦ ⑧ ⑨

¿Cuál es su paso normal al caminar para ir al trabajo?

- ◯ Lento
- ◯ Rápido
- ◯ Regular
- ◯ Muy rápido

¿Cuántos pisos (escaleras) sube en su trabajo regularmente durante el día?

- ◯ Ninguno
- ◯ 5-9 pisos
- ◯ 1-2 pisos
- ◯ 10-14 pisos
- ◯ 3-4 pisos
- ◯ 15 ó más pisos

¿Usted considera que la actividad física que realiza en su trabajo es?

◯ Ligera ◯ Moderada ◯ Intensa

Constitución Corporal.

75.- Datos de usted (mujeres)

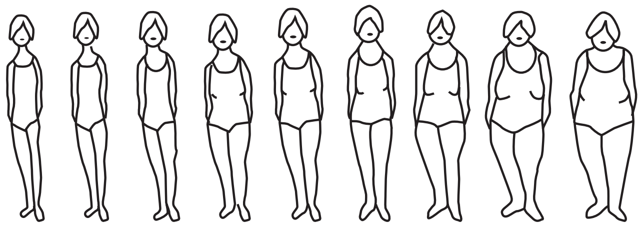

1 2 3 4 5 6 7 8 9

De las figuras que aparecen en la parte izquierda, por favor señale cuál representa mejor su figura en cada edad.

Edad	1	2	3	4	5	6	7	8	9
A los 10 años	◯	◯	◯	◯	◯	◯	◯	◯	◯
A los 15 años	◯	◯	◯	◯	◯	◯	◯	◯	◯
A los 20 años	◯	◯	◯	◯	◯	◯	◯	◯	◯
A los 25 años	◯	◯	◯	◯	◯	◯	◯	◯	◯
A los 30 años	◯	◯	◯	◯	◯	◯	◯	◯	◯
A los 35 años	◯	◯	◯	◯	◯	◯	◯	◯	◯
A los 40 años	◯	◯	◯	◯	◯	◯	◯	◯	◯
A los 45 años	◯	◯	◯	◯	◯	◯	◯	◯	◯
A los 50 años	◯	◯	◯	◯	◯	◯	◯	◯	◯
A los 55 años	◯	◯	◯	◯	◯	◯	◯	◯	◯
A los 60 años	◯	◯	◯	◯	◯	◯	◯	◯	◯
A los 65 años	◯	◯	◯	◯	◯	◯	◯	◯	◯
Actualmente	◯	◯	◯	◯	◯	◯	◯	◯	◯

76.- Datos de usted (hombres)

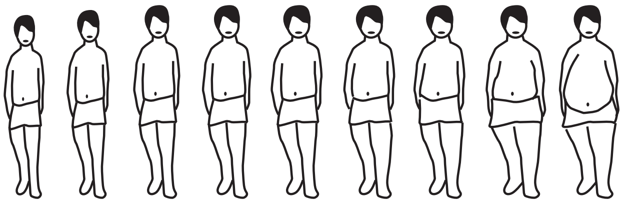

1 2 3 4 5 6 7 8 9

De las figuras que aparecen en la parte izquierda, por favor señale cuál representa mejor su figura en cada edad.

Edad	1	2	3	4	5	6	7	8	9
A los 10 años	◯	◯	◯	◯	◯	◯	◯	◯	◯
A los 15 años	◯	◯	◯	◯	◯	◯	◯	◯	◯
A los 20 años	◯	◯	◯	◯	◯	◯	◯	◯	◯
A los 25 años	◯	◯	◯	◯	◯	◯	◯	◯	◯
A los 30 años	◯	◯	◯	◯	◯	◯	◯	◯	◯
A los 35 años	◯	◯	◯	◯	◯	◯	◯	◯	◯
A los 40 años	◯	◯	◯	◯	◯	◯	◯	◯	◯
A los 45 años	◯	◯	◯	◯	◯	◯	◯	◯	◯
A los 50 años	◯	◯	◯	◯	◯	◯	◯	◯	◯
A los 55 años	◯	◯	◯	◯	◯	◯	◯	◯	◯
A los 60 años	◯	◯	◯	◯	◯	◯	◯	◯	◯
A los 65 años	◯	◯	◯	◯	◯	◯	◯	◯	◯
Actualmente	◯	◯	◯	◯	◯	◯	◯	◯	◯

De las figuras que aparecen, por favor señale cuál representa mejor la figura de sus padres en cada edad.

77.- Datos de su madre

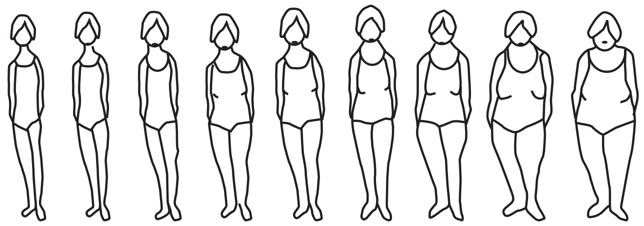

① ② ③ ④ ⑤ ⑥ ⑦ ⑧ ⑨

¿Qué edad tiene su madre actualmente (en caso de que viva)?

|_|_| años

⓪ ① ② ③ ④ ⑤ ⑥ ⑦ ⑧ ⑨
⓪ ① ② ③ ④ ⑤ ⑥ ⑦ ⑧ ⑨

78.- Datos de su padre

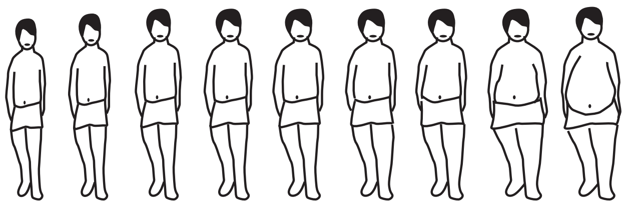

① ② ③ ④ ⑤ ⑥ ⑦ ⑧ ⑨

¿Qué edad tiene su padre actualmente (en caso de que viva)?

|_|_| años

⓪ ① ② ③ ④ ⑤ ⑥ ⑦ ⑧ ⑨
⓪ ① ② ③ ④ ⑤ ⑥ ⑦ ⑧ ⑨

79.- Estatura (cm)	80.- Peso actual (kg)	81.- Peso a los 21 años
ESCRIBIR		
⓪⓪⓪	⓪⓪⓪	⓪⓪⓪
①①①	①①①	①①①
②②②	②②②	②②②
③③③	③③③	③③③
④④④	④④④	④④④
⑤⑤⑤	⑤⑤⑤	⑤⑤⑤
⑥⑥⑥	⑥⑥⑥	⑥⑥⑥
⑦⑦⑦	⑦⑦⑦	⑦⑦⑦
⑧⑧⑧	⑧⑧⑧	⑧⑧⑧
⑨⑨⑨	⑨⑨⑨	⑨⑨⑨

82.- ¿Cambió de peso en los últimos 5 años?

○ Sí ○ No

Ganancia (Kg.)		Pérdida (Kg.)	
○ + 2 a 4	○ +15 a 19	○ - 2 a 4	○ - 15 a 19
○ + 5 a 9	○ + 20 a 29	○ - 5 a 9	○ - 20 ó más
○ + 10 a 14	○ + 30 ó más	○ - 10 a 14	

83.- ¿Cambió de peso en el último año?

○ Sí ○ No

Ganancia (Kg.)		Pérdida (Kg.)	
○ + 2 a 4	○ +15 a 19	○ - 2 a 4	○ - 15 a 19
○ + 5 a 9	○ + 20 a 29	○ - 5 a 9	○ - 20 ó más
○ + 10 a 14	○ + 30 ó más	○ - 10 a 14	

84.- Sus cifras de presión arterial actualmente son:

Diastólica (mínima)		Sistólica (máxima)	
○ No sabe	○ 90-94 mm Hg	○ No sabe	○ 150-159 mm Hg
○ menos de 75 mm Hg	○ 95-104 mm Hg	○ menos de 120 mm Hg	○ 160-169 mm Hg
○ 75-84 mm Hg	○ 105 ó más mm Hg	○ 120-139 mm Hg	○ 170 ó más mm Hg
○ 84-89 mm Hg		○ 140-149 mm Hg	

85.- El sobre que se le entregó contiene una cinta métrica para que lleve a cabo las siguientes mediciones

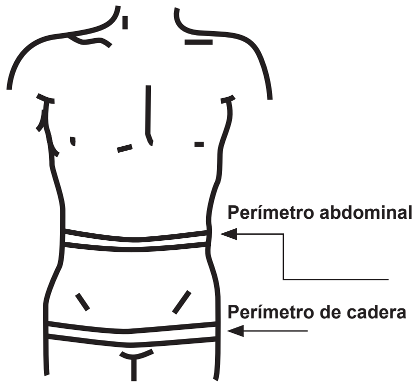

Instrucciones especiales para la Medición de perímetros

Adjunta se encuentra una cinta métrica para que pueda realizar estas mediciones.

Siga los siguientes pasos para realizar la medición correctamente:

1. Lleve a cabo las mediciones estando de pie.
2. Evite hacer las mediciones sobre ropa gruesa.
3. Reporte la medición lo más cercano al centímetro.
4. La medición de abdomen tómela a la altura del ombligo.
5. La medición de cadera tómela a la altura del punto más ancho de la cadera.

Perímetro abdominal (cm)	Perímetro de cadera (cm)
⓪⓪⓪	⓪⓪⓪
①①①	①①①
②②②	②②②
③③③	③③③
④④④	④④④
⑤⑤⑤	⑤⑤⑤
⑥⑥⑥	⑥⑥⑥
⑦⑦⑦	⑦⑦⑦
⑧⑧⑧	⑧⑧⑧
⑨⑨⑨	⑨⑨⑨

Las siguientes preguntas tienen como propósito conocer su opinión sobre el peso de las personas. Es por ello que lo más importante es su respuesta sincera, su opinión es lo que cuenta. De ninguna manera se trata de que usted se sienta en la necesidad de responder según lo que otros opinan, por ejemplo, lo que los médicos opinan o lo que ha leido en revistas, etc.; su opinión puede ser contraria a lo que otros refieren, esto no quiere decir que usted esté en un error, simplemente tiene opiniones distintas. Para conocer su opinión, puede escoger una de las siguientes opciones de respuesta para cada pregunta, cada opción de respuestas tiene un número (del 1 al 5):

① Totalmente de acuerdo ② Más de acuerdo que en desacuerdo ③ Ni de acuerdo, ni en desacuerdo ④ Más en desacuerdo que en acuerdo ⑤ Totalmente en desacuerdo

86.- La obesidad es:	Rellene el círculo de su respuesta				
Una muestra de buena salud a cualquier edad	①	②	③	④	⑤
Un efecto de cierta manera personal de vivir (comer mucho, no hacer ejercicio, etc)	①	②	③	④	⑤
Una forma de ser que no debe preocuparnos	①	②	③	④	⑤
Una condición que siempre se debe evitar	①	②	③	④	⑤
Una característica que se hereda de familia	①	②	③	④	⑤
Un problema de salud sólo para algunos (médicos, las adolescentes, comerciantes)	①	②	③	④	⑤
**87.- En general las personas obesas, son:**	**Rellene el círculo de su respuesta**				
Débiles	①	②	③	④	⑤
Como cualquier otra persona al llevarse con los demás	①	②	③	④	⑤
Desagradables	①	②	③	④	⑤
Gente que requiere un trato especial	①	②	③	④	⑤
Fuertes	①	②	③	④	⑤
Propensas a enfermarse	①	②	③	④	⑤
Encantadoras	①	②	③	④	⑤
Personas que se mantienen en buena salud	①	②	③	④	⑤

Antecedentes de problemas músculo - esqueléticos

88.- ¿Ha tenido en los últimos 7 días algún problema como dolor a la presión, inflamación o rigidez en sus huesos, sus articulaciones (coyunturas) o sus músculos?

○ Sí ○ No (pase a la siguiente pregunta)

¿Desde cuándo tiene este problema?

Años |_|_| ⓪ ① ② ③ ④ ⑤ ⑥ ⑦ ⑧ ⑨ / ⓪ ① ② ③ ④ ⑤ ⑥ ⑦ ⑧ ⑨

Meses |_|_| ⓪ ① ② ③ ④ ⑤ ⑥ ⑦ ⑧ ⑨ ⑩ ⑪ ⑫

Días |_|_| ⓪ ① ② ③ ④ ⑤ ⑥ ⑦ ⑧ ⑨ / ⓪ ① ② ③ ④ ⑤ ⑥ ⑦ ⑧ ⑨

Rellene el círculo que señale el sitio donde tuvo esta molestia o dolor:

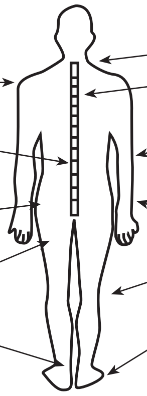

○ Hombros
○ Espalda baja
○ Cadera
○ Muslos
○ Tobillos
○ Cuello
○ Espalda alta
○ Codos
○ Muñecas y manos
○ Rodillas
○ Dedos de los pies

¿Cómo describiría la intensidad de su dolor en los últimos 7 días?
Seleccione el círculo que mejor describa la intensidad del dolor

Dolor muy leve ○ 1 ○ 2 ○ 3 ○ 4 ○ 5 ○ 6 ○ 7 ○ 8 ○ 9 ○ 10 Dolor muy inteso

¿Existió algún problema traumático (por ejemplo una torcedura o un golpe) **que haya causado este dolor, inflamación o rigidez?**

○ Sí ○ No

¿Qué fue lo que le produjo el problema?

○ Una fractura (ruptura de un hueso)
○ Un golpe en un accidente automovilístico
○ Una torcedura
○ Otro: (Especifique): ____________________

88a) NO RELLENAR
⓪ ① ② ③ ④ ⑤ ⑥ ⑦ ⑧ ⑨

¿Está recibiendo tratamiento para el dolor, la inflamación o la rigidez en sus huesos, articulaciones o músculos?

○ Sí ○ No

¿Quién le mandó el tratamiento? Puede llenar más de un opción.

○ Médico general
○ Farmacéutico
○ Especialista (Reumatólogo, traumatólogo, otro)
○ Fisioterapeuta
○ Quiropráctico
○ Acupunturista
○ Curandero
○ Usted mismo se recomendó remedios caseros (por ejemplo ungüentos)
○ Otro: (Especifique) ____________________

88b) NO RELLENAR
⓪ ① ② ③ ④ ⑤ ⑥ ⑦ ⑧ ⑨

89.- ¿En algún momento en la vida ha tenido algún problema como dolor, inflamación o rigidez en sus huesos por más de 2 semanas, pero actualmente ya no la tiene?

○ No (pase a la siguiente pregunta)
○ Sí, ¿Cuánto tiempo duró este problema?

Meses |_|_| ⓪ ① ② ③ ④ ⑤ ⑥ ⑦ ⑧ ⑨ / ⓪ ① ② ③ ④ ⑤ ⑥ ⑦ ⑧ ⑨

¿Cuándo fue esto?

○ Antes de 1998 ○ 1998 ○ 1999 ○ 2000
○ 2001 ○ 2002 ○ 2003 ○ 2004
○ 2005 ○ 2006 ○ 2007 ○ 2009

Rellene el círculo que señale el sitio donde tuvo esta molestia o dolor:

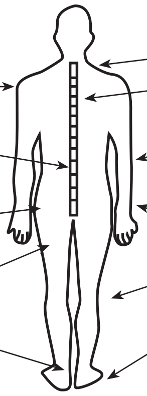

○ Hombros
○ Espalda baja
○ Cadera
○ Muslos
○ Tobillos
○ Cuello
○ Espalda alta
○ Codos
○ Muñecas y manos
○ Rodillas
○ Dedos de los pies

¿Cómo describiría la intensidad de su dolor en esa ocasión?
Seleccione el círculo que mejor describa la intensidad del dolor

Dolor muy leve ○ 1 ○ 2 ○ 3 ○ 4 ○ 5 ○ 6 ○ 7 ○ 8 ○ 9 ○ 10 Dolor muy intenso

¿Existió algún problema traumático (por ejemplo una torcedura o un golpe) **que haya causado este dolor, inflamación o rigidez?**

○ Sí ○ No

¿Qué fue lo que le produjo el problema?

○ Una fractura (ruptura de un hueso)
○ Un golpe en un accidente automovilístico
○ Una torcedura
○ Otro: (Especifique) ____________________

89a) NO RELLENAR
⓪ ① ② ③ ④ ⑤ ⑥ ⑦ ⑧ ⑨

¿Alguna de las personas anteriores le dijo el nombre de la enfermedad o el diagnóstico de su problema?

○ No ○ Sí, ¿Qué problema le dijo que tenía?
(Especifique) ____________________

89b) NO RELLENAR
⓪ ① ② ③ ④ ⑤ ⑥ ⑦ ⑧ ⑨

¿Cuáles de los siguientes tratamientos usó para el dolor, inflamación o rigidez?

Automedicación (no ordenada por su médico)	○ Sí	○ No
Medicamentos ordenados por un médico	○ Sí	○ No
Inyecciones	○ Sí	○ No
Fisioterapia	○ Sí	○ No
Cirugía	○ Sí	○ No
Dieta	○ Sí	○ No
Otro: (Especifique) ____________________		

89c) NO RELLENAR
⓪ ① ② ③ ④ ⑤ ⑥ ⑦ ⑧ ⑨

¿Qué tan bien ha podido adaptarse a su problema?

○ Muy bien ○ No tan bien
○ Bien ○ Nada bien

90.- Queremos conocer más sobre la forma como están o estuvieron limitadas sus actividades por su problema, es decir por el dolor, la inflamación o la rigidez en sus huesos, articulaciones o músculos. Estamos especialmente interesados en conocer si ha tenido dificultad para realizar actividades específicas durante el último año.

¿Ha estado limitado, en el último año, en el tipo de actividades que puede hacer debido a un problema como dolor o rigidez en sus huesos, articulaciones o músculos?

◯ No he estado limitado (pase a la siguiente pregunta)

◯ Sí, actualmente me encuentro limitado para realizar algunas actividades (Especifique la duración de esa limitación, días, semanas o meses)

Días ① ② ③ ④ ⑤ ⑥ ⑦

Semanas ① ② ③ ④ ⑤

Meses
⓪ ① ② ③ ④ ⑤ ⑥ ⑦ ⑧ ⑨
⓪ ① ② ③ ④ ⑤ ⑥ ⑦ ⑧ ⑨

◯ Sí, estuve limitado durante el último año, pero ya no (Especifique la duración de esa limitación, días, semanas o meses)

Días ① ② ③ ④ ⑤ ⑥ ⑦

Semanas ① ② ③ ④ ⑤

Meses
⓪ ① ② ③ ④ ⑤ ⑥ ⑦ ⑧ ⑨
⓪ ① ② ③ ④ ⑤ ⑥ ⑦ ⑧ ⑨

Selecciona el círculo que representa la limitación para estas actividades.

A = Actualmente **P** = En el último año, pero actualmente **NO**

	Sin dificultad		Con cierta dificultad pero lo logró o lograba		Sólo lo logró o lograba con ayuda de otro		Aún con ayuda no puede o no podía hacerlo		No puede o no podía por causas ajenas a su artritis	
	**A**	**P**	**A**	**P**	**A**	**P**	**A**	**P**	**A**	**P**
Caminar en terrenos planos	◯	◯	◯	◯	◯	◯	◯	◯	◯	◯
Levantarse de una silla recta sin apoyarse con las manos	◯	◯	◯	◯	◯	◯	◯	◯	◯	◯
Sentarse y levantarse de la taza del sanitario	◯	◯	◯	◯	◯	◯	◯	◯	◯	◯
Sacar la ropa del armario (ropero) y cajones	◯	◯	◯	◯	◯	◯	◯	◯	◯	◯
Vestirse solo(a) (incluso abotonarse y usar cierre)	◯	◯	◯	◯	◯	◯	◯	◯	◯	◯
Lavarse el cabello	◯	◯	◯	◯	◯	◯	◯	◯	◯	◯
Lavarse y secarse el cuerpo	◯	◯	◯	◯	◯	◯	◯	◯	◯	◯
Peinarse	◯	◯	◯	◯	◯	◯	◯	◯	◯	◯
Comer por si mismo	◯	◯	◯	◯	◯	◯	◯	◯	◯	◯
Llevarse a la boca un vaso con líquido	◯	◯	◯	◯	◯	◯	◯	◯	◯	◯
Abrir y cerrar las llaves del agua	◯	◯	◯	◯	◯	◯	◯	◯	◯	◯
Alcanzar y bajar una bolsa de 2Kg. que esté por arriba de su cabeza	◯	◯	◯	◯	◯	◯	◯	◯	◯	◯
Abrir las puertas de un coche	◯	◯	◯	◯	◯	◯	◯	◯	◯	◯
Abrir un tapón de rosca no muy apretado	◯	◯	◯	◯	◯	◯	◯	◯	◯	◯
Escribir un recado	◯	◯	◯	◯	◯	◯	◯	◯	◯	◯
Conducir un coche o coser a máquina (no eléctrica)	◯	◯	◯	◯	◯	◯	◯	◯	◯	◯
Salir de compras	◯	◯	◯	◯	◯	◯	◯	◯	◯	◯
Acuclillarse	◯	◯	◯	◯	◯	◯	◯	◯	◯	◯
Arrodillarse	◯	◯	◯	◯	◯	◯	◯	◯	◯	◯

A = Actualmente **P** = En el último año, pero actualmente no

91.- ¿Durante los últimos 2 años algún médico le ha dicho que es hipertenso o que tiene la presión alta?

◯ Sí ◯ No (pase a la siguiente pregunta)

¿Cuántos meses tiene con la hipertensión?

meses [|]

⓪ ① ② ③ ④ ⑤ ⑥ ⑦ ⑧ ⑨
⓪ ① ② ③ ④ ⑤ ⑥ ⑦ ⑧ ⑨

¿Toma medicamentos para su presión?

◯ Sí ◯ No

¿Cuáles medicamentos toma actualmente?

a) ______
b) ______
c) ______

91a) NO RELLENAR
⓪ ① ② ③ ④ ⑤ ⑥ ⑦ ⑧ ⑨
⓪ ① ② ③ ④ ⑤ ⑥ ⑦ ⑧ ⑨
⓪ ① ② ③ ④ ⑤ ⑥ ⑦ ⑧ ⑨

¿Cuántas cápsulas o tabletas toma al día?

a) ______ / día
b) ______ / día
c) ______ / día

91b) NO RELLENAR
⓪ ① ② ③ ④ ⑤ ⑥ ⑦ ⑧ ⑨
⓪ ① ② ③ ④ ⑤ ⑥ ⑦ ⑧ ⑨
⓪ ① ② ③ ④ ⑤ ⑥ ⑦ ⑧ ⑨

¿Desde cuándo toma este medicamento?
Señale el número de meses que ha usado cada medicamento durante los últimos 2 años.

Durante

a) Menos de 1 mes ◯
① ② ③ ④ ⑤ ⑥ ⑦ ⑧ ⑨ [|]
⓪ ① ② ③ ④ ⑤ ⑥ ⑦ ⑧ ⑨ meses

b) Menos de 1 mes ◯
① ② ③ ④ ⑤ ⑥ ⑦ ⑧ ⑨ [|]
⓪ ① ② ③ ④ ⑤ ⑥ ⑦ ⑧ ⑨ meses

c) Menos de 1 mes ◯
① ② ③ ④ ⑤ ⑥ ⑦ ⑧ ⑨ [|]
⓪ ① ② ③ ④ ⑤ ⑥ ⑦ ⑧ ⑨ meses

92.- ¿Durante los últimos 3 años ha tenido dolor o malestar en el pecho?

◯ Sí ◯ No (pase a la siguiente pregunta)

¿Este dolor o malestar se presenta al caminar cuesta arriba (de subida) o al caminar rápidamente?

◯ Sí ◯ No
◯ Nunca camino rápido o cuesta arriba

¿Este dolor o malestar se presenta al caminar normalmente (en plano)?

◯ Sí ◯ No

¿Qué hace usted si siente el dolor o la molestia al ir caminando?

◯ Me paro o camino más despacio
◯ Sigo caminando igual

¿Si se queda parado qué le pasa al dolor o a la molestia?

◯ Se le quita o disminuye
◯ No se le quita

¿En cuánto tiempo se le quita o cuánto tiempo dura?

◯ 10 min. o menos
◯ Más de 10 min.

¿Ha tenido este dolor o molestia en más de tres ocasiones?

◯ Sí ◯ No

¿Algunas de estas cosas le provoca este dolor o molestia?

Una emoción	Sí	No
Inclinarse	Sí	No
Comer	Sí	No
Respirar	Sí	No
Toser	Sí	No
Un aire frío (un enfriamiento)	Sí	No

◯ Otro: ______________________
(Especifique)

92a) NO RELLENAR
⓪ ① ② ③ ④ ⑤ ⑥ ⑦ ⑧ ⑨
⓪ ① ② ③ ④ ⑤ ⑥ ⑦ ⑧ ⑨

Señale dónde le duele o molesta

◯ Otro: ______________________
Especifique

92b) NO RELLENAR
⓪ ① ② ③ ④ ⑤ ⑥ ⑦ ⑧ ⑨

92c) NO RELLENAR
⓪ ① ② ③ ④ ⑤ ⑥ ⑦ ⑧ ⑨

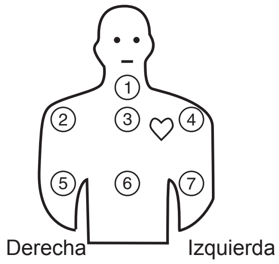

¿Esta sensación (dolor) es peor cuando tiene catarro o tos?

◯ Sí ◯ No

¿Cómo describiría esta sensación?

◯ Como molestia
◯ Como dolor

¿Alguna de estas palabras describe la sensación?

◯ Pesantez
◯ Punzada
◯ Opresión
◯ Ardor
◯ Otra: ______________________
(Especifique)

93.- ¿Durante los últimos 3 años ha tenido un dolor intenso en el pecho (muy fuerte) y que le duró más de media hora?

◯ Sí ◯ No (pase a la siguiente pregunta)

¿Cuántos episodios de estos ha tenido?

Número de episodios: |_|_| ⓪ ① ② ③ ④ ⑤ ⑥ ⑦ ⑧ ⑨
Más de ⑨

Anote sólo la fecha del primero

1º
mes ⓪ ①
⓪ ① ② ③ ④ ⑤ ⑥ ⑦ ⑧ ⑨ |_|_|
año ⓪ ① ② ③ ④ ⑤ ⑥ ⑦ ⑧ ⑨
⓪ ① ② ③ ④ ⑤ ⑥ ⑦ ⑧ ⑨ |_|_|

94.- ¿Siente dolor en alguna de las piernas al caminar?

◯ Sí ◯ No

¿Siente dolor en alguna de sus piernas al estar sentado o de pie sin caminar?

◯ Sí ◯ No

◯ Incluye pantorrilla ◯ No incluye pantorrilla

¿Siente dolor al caminar cuesta arriba (de subida) o rápido?

◯ Sí ◯ No

¿Siente dolor al caminar normalmente?

◯ Sí ◯ No

¿El dolor desaparece al ir caminando?

◯ Sí ◯ No

¿Qué hace usted cuando va caminando y le da el dolor?

◯ Sigo caminado igual
◯ Me paro o camino más despacio

¿Si se para, qué pasa con el dolor?

◯ Se me quita ◯ No se me quita

¿Cuánto tiempo le dura?

◯ Menos de 10 min. ◯ Más de 10 min.

95.- ¿Durante los últimos 3 años le ha dicho algún medico que tuvo usted una embolia en el cerebro (un coágulo, una trombosis o una isquemia)?

◯ Sí ◯ No

¿Cuándo fue?

mes ⓪ ①
⓪ ① ② ③ ④ ⑤ ⑥ ⑦ ⑧ ⑨ |_|_| mes

año
2007 ◯
2008 ◯
2009 ◯
2010 ◯
2011 ◯

96.- ¿Durante los últimos 3 años, ha presentado de pronto debilidad o pérdida de fuerza en alguna(s) parte(s) de su cuerpo?

◯ Sí ◯ No

¿En cuál (es)?

◯ En un brazo ◯ Pierna y brazo del mismo lado
◯ En una pierna ◯ La mitad de la cara

¿Durante los últimos 3 años, ha sentido que de pronto deja de sentir o que se le durmió alguna(s) parte(s) de su cuerpo?

◯ Sí ◯ No

¿En cuál (es)?

◯ En un brazo ◯ Pierna y brazo del mismo lado
◯ En una pierna ◯ La mitad de la cara
◯ Otra

¿Durante los últimos 3 años, ha quedado completamente ciego de un ojo y después ha vuelto a ver bien?

◯ Sí ◯ No

¿Durante los últimos 3 años ha visto realmente doble (no borroso)**?**

◯ Sí ◯ No

¿Durante los últimos 3 años, ha tenido problemas para hablar (como arrastrar las palabras o no poder decir lo que estaba pensando)?

◯ Sí ◯ No

¿Durante los últimos 3 años, se ha caído al suelo sin ninguna razón aparente (como tropezarse o sufrir un mareo)?

◯ Sí ◯ No

¿Lo tuvieron que hospitalizar por alguno de estos problemas?

◯ Sí ◯ No

¿Siente que le falta aire cuando camina cuesta arriba?

◯ Sí ◯ No

¿Siente que le falta aire cuando está caminando con otra persona de su misma edad en lugares planos?

◯ Sí ◯ No

¿Tiene falta de aire cuando está caminado a su propio paso?

◯ Sí ◯ No

97.- Esta sección pretende verificar si usted o sus familiares han padecido las patologías mencionadas, así como la forma en que se hizo el diagnóstico, y el tipo de tratamiento que recibió

¿Padece osteoporosis?

◯ Sí ◯ No (pase a la siguiente pregunta)

¿Desde hace cuántos años?

años |_|_|

⓪ ① ② ③ ④ ⑤ ⑥ ⑦ ⑧ ⑨
⓪ ① ② ③ ④ ⑤ ⑥ ⑦ ⑧ ⑨

¿Quién le dio este diagnóstico?

◯ Médico ◯ Usted mismo
◯ Otra persona (especifique) ____________________

97a) NO RELLENAR
⓪ ① ② ③ ④ ⑤ ⑥ ⑦ ⑧ ⑨

¿Le realizaron algún estudio para confirmar este diagnóstico?

◯ No ◯ Sí ► ¿Cuál? (Especifique)____________________

97b) NO RELLENAR
⓪ ① ② ③ ④ ⑤ ⑥ ⑦ ⑧ ⑨

¿Recibió tratamiento para este problema?

◯ Sí ◯ No

¿Qué tipo de tratamiento recibió?

◯ Hormonales ◯ Calcio
◯ Otro: (Especifique)______________

97c) NO RELLENAR
⓪ ① ② ③ ④ ⑤ ⑥ ⑦ ⑧ ⑨

¿Cuánto tiempo recibió este tratamiento?

años |_|_|

⓪ ① ② ③ ④ ⑤ ⑥ ⑦ ⑧ ⑨
⓪ ① ② ③ ④ ⑤ ⑥ ⑦ ⑧ ⑨

¿Alguno de sus familiares padece (o padeció) osteoporosis?

◯ Sí ◯ No ◯ No sabe

¿Cuál (es) de sus familiares?

◯ Madre ◯ Padre ◯ Abuela materna ◯ Abuelo paterno
◯ Abuela paterna ◯ Abuelo materno
◯ Otro: (Especifique) ____________

97d) NO RELLENAR
⓪ ① ② ③ ④ ⑤ ⑥ ⑦ ⑧ ⑨

98.- ¿Qué tan frecuentemente siente usted (o su familia) que el dinero del que dispone no es suficiente para cubrir algunas de las siguientes necesidades?

	Todo el tiempo	Frecuentemente	En ocasiones	Casi nunca
El tipo de comida que su familia debe tener	◯	◯	◯	◯
El tipo de atención médica que su familia debe tener	◯	◯	◯	◯
El tipo de ropa y vestido que su familia debe tener	◯	◯	◯	◯
El tipo de actividades recreativas y de esparcimiento que su familia debe tener	◯	◯	◯	◯

Estrés Social y Salud Mental

El estrés puede ser un factor crónico o agudo en la vida del individuo. Esta sección del cuestionario tiene la finalidad de colectar información sobre los principales factores estresantes en la vida como los eventos estresantes y el estrés económico.

99.- ¿Cuántas veces durante el último año se ha sentido amenazado físicamente?

◯ Nunca
◯ Varias veces al año
◯ Por lo menos una vez al mes
◯ Por lo menos una vez por semana
◯ Casi todos los días

100.- ¿En el último año qué tan seguro se ha sentido en su casa o colonia?

	Muy seguro	Un poco seguro	Nada seguro
Casa	◯	◯	◯
Colonia	◯	◯	◯

Eventos de vida

101.- Las siguientes preguntas se refieren a situaciones o eventos que pudieron haberle sucedido en el último año:			Cuando ocurrió esta situación, ¿suspendió las actividades que realizaba?, ¿Por qué período las suspendió?				¿Desde entonces qué tan a menudo piensa en lo que ocurrió?			
	Sí	No	Largo	Moderado	Pequeño	No suspendió actividades	Muy a menudo	A menudo	Rara vez	Muy rara vez
Se enfermó gravemente.	○	○	○	○	○	○	○	○	○	○
Se enfermó gravemente alguien cercano a usted.	○	○	○	○	○	○	○	○	○	○
Se lesionó gravemente.	○	○	○	○	○	○	○	○	○	○
Se lesionó gravemente alguien cercano a usted.	○	○	○	○	○	○	○	○	○	○
Perdió la visión o la audición.	○	○	○	○	○	○	○	○	○	○
Se incrementaron sus dificultades para caminar o realizar otras actividades de la vida diaria.	○	○	○	○	○	○	○	○	○	○
Se jubiló.	○	○	○	○	○	○	○	○	○	○
Tuvo problemas con sus vecinos.	○	○	○	○	○	○	○	○	○	○
Tuvo problemas financieros.	○	○	○	○	○	○	○	○	○	○
Fue robado o asaltado.	○	○	○	○	○	○	○	○	○	○
Separación matrimonial o divorcio.	○	○	○	○	○	○	○	○	○	○
Tuvo una enfermera o cuidadora en casa.	○	○	○	○	○	○	○	○	○	○
Mudanza.	○	○	○	○	○	○	○	○	○	○
Muerte de una mascota.	○	○	○	○	○	○	○	○	○	○
¿Alguna otra situación? (Por favor díganos cuál recuerda)	○	○	○	○	○	○	○	○	○	○
______ Especifique — 101a) NO RELLENAR ⓪①②③④⑤⑥⑦⑧⑨ ⓪①②③④⑤⑥⑦⑧⑨			○	○	○	○	○	○	○	○
______ Especifique — 101b) NO RELLENAR ⓪①②③④⑤⑥⑦⑧⑨ ⓪①②③④⑤⑥⑦⑧⑨			○	○	○	○	○	○	○	○

102.- ¿Alguien cercano a usted falleció en el último año? ○ Sí ○ No → Pase a la siguiente pregunta

Con respecto a la persona que falleció ¿Qué relación tenía con ella? 102) NO RELLENAR ⓪①②③④⑤⑥⑦⑧⑨	¿Cuánto piensa en la persona que falleció?				¿Usted tenía idea de que iba a morir pronto o fue inesperado?	
	Muy frecuentemente	Frecuentemente	Algo	Nada	Tenía idea	Fue inesperado
○ Mi padre	○	○	○	○	○	○
○ Mi madre	○	○	○	○	○	○
○ Mi esposo(a)/pareja	○	○	○	○	○	○
○ Mi hermano(a)	○	○	○	○	○	○
○ Mi hijo(a)	○	○	○	○	○	○
○ Mi nieto(a)	○	○	○	○	○	○
○ Mi yerno	○	○	○	○	○	○
○ Mi nuera	○	○	○	○	○	○
○ Mi amigo	○	○	○	○	○	○
○ Otro Especifique: ______	○	○	○	○	○	○

103.- Rellene el círculo de la opción que considere más adecuada para cada pregunta.

○ **No trabajo (pase a la siguiente pregunta)**

	Muy rara vez	Rara vez	Indeciso(a)	Frecuentemente	Muy frecuentemente
¿Aprende cosas nuevas en su trabajo?	○	○	○	○	○
¿Hay discusiones entre grupos de personas en su sitio de trabajo?	○	○	○	○	○
¿Con qué frecuencia piensa en su trabajo cuando está en casa?	○	○	○	○	○
¿Tiene usted oportunidades de aplicar sus conocimientos y habilidades en su trabajo?	○	○	○	○	○
¿Está usted muy estresado(a) en su trabajo?	○	○	○	○	○
¿Siente usted que puede ser sustituido por cualquier persona en su trabajo?	○	○	○	○	○
¿Se entera usted de los planes sobre cambios en su trabajo?	○	○	○	○	○
¿Puede influir usted sobre los cambios en su trabajo?	○	○	○	○	○
¿Su jefe inmediato, es capaz de apreciar las dificultades que tiene usted en su trabajo?	○	○	○	○	○
¿Sus problemas personales son tomados en cuenta por su jefe inmediato?	○	○	○	○	○
¿Su jefe inmediato lo(a) trata justamente?	○	○	○	○	○

104.- Lea con cuidado cada una de las siguientes afirmaciones y rellene el círculo de la opción que mejor corresponda.	Nunca	Menos de 10 veces al año	1 vez por mes	2 a 3 veces por mes	1 vez por semana	2 a 5 veces por semana	Todos los días
Me siento emocionalmente agotado(a) por mi trabajo	◯	◯	◯	◯	◯	◯	◯
Me siento cansado(a) al final de la jornada de trabajo	◯	◯	◯	◯	◯	◯	◯
Me siento fatigado(a) cuando me levanto por la mañana y tengo que ir a trabajar	◯	◯	◯	◯	◯	◯	◯
Comprendo fácilmente cómo se sienten las personas que atiendo en mi trabajo	◯	◯	◯	◯	◯	◯	◯
Creo que trato a las personas como si fueran objetos impersonales	◯	◯	◯	◯	◯	◯	◯
Trabajar todo el día con mucha gente es un esfuerzo	◯	◯	◯	◯	◯	◯	◯
Trato muy eficazmente los problemas de las personas que atiendo en mi trabajo	◯	◯	◯	◯	◯	◯	◯
Me siento exhausto por mi trabajo	◯	◯	◯	◯	◯	◯	◯
Creo que influyo positivamente con mi trabajo en la vida de las personas	◯	◯	◯	◯	◯	◯	◯
Me he vuelto más insensible con la gente, desde que ejerzo este trabajo	◯	◯	◯	◯	◯	◯	◯
Me preocupa el hecho de que este trabajo me endurezca emocionalmente	◯	◯	◯	◯	◯	◯	◯
Me siento muy activo(a)	◯	◯	◯	◯	◯	◯	◯
Me siento frustrado(a) en mi trabajo	◯	◯	◯	◯	◯	◯	◯
Creo que estoy trabajando demasiado	◯	◯	◯	◯	◯	◯	◯
Realmente me preocupa lo que le ocurre a las personas que atiendo	◯	◯	◯	◯	◯	◯	◯
Trabajar directamente con las personas me produce estrés	◯	◯	◯	◯	◯	◯	◯
Puedo crear fácilmente una atmósfera relajada con las personas que atiendo	◯	◯	◯	◯	◯	◯	◯
He conseguido muchas cosas útiles en mi trabajo	◯	◯	◯	◯	◯	◯	◯
Me siento cansado(a)	◯	◯	◯	◯	◯	◯	◯
En mi trabajo trato los problemas emocionalmente con mucha calma	◯	◯	◯	◯	◯	◯	◯
Siento que las personas que atiendo en mi trabajo me culpan por sus problemas	◯	◯	◯	◯	◯	◯	◯

¿Cuánta tensión le causa a usted el trabajo monótono?

◯ Ninguna ◯ Poca ◯ Regular ◯ Mucha ◯ Demasiada

	Muy satisfecho(a)	Satisfecho(a)	Indeciso(a)	Insatisfecho(a)	Muy insatisfecho(a)
¿Está usted satisfecho(a) con lo interesante y variado de su trabajo?	◯	◯	◯	◯	◯
¿Qué tan satisfecho(a) está usted con la actitud de su jefe inmediato?	◯	◯	◯	◯	◯
¿Qué tan satisfecho(a) está usted con sus compañeros de trabajo?	◯	◯	◯	◯	◯

105.- La relación con mis compañeros de trabajo es:

◯ Muy constructiva
◯ Constructiva
◯ Indiferente
◯ Destructiva

El reconocimiento que recibo por mi trabajo es:

◯ Muy satisfactorio
◯ Satisfactorio
◯ Insatisfactorio
◯ Regular

Mis actividades cotidianas son:

◯ Muy estimulantes
◯ Rutinarias
◯ Estimulantes
◯ Muy rutinarias
◯ Inmediatas

Estrés Económico

Las siguientes preguntas tienen como finalidad conocer la manera en que su pareja y otros miembros de la familia realizan el aporte económico para los gastos de la casa y de la manera en que disponen de él. Llene la opción que corresponda mejor a su situación.

106.- ¿Cuántas personas dependen económicamente de usted? |__|__| personas

106) NO RELLENAR
⓪ ① ② ③ ④ ⑤ ⑥ ⑦ ⑧ ⑨

107.- ¿El ingreso del hogar...

¿Lo maneja su pareja y le da gasto?	◯ Sí	◯ No
¿Se coloca en un fondo común y cada quien utiliza lo que necesita?	◯ Sí	◯ No
¿Su pareja le da lo que gana y usted decide cómo se gasta?	◯ Sí	◯ No
¿Cada quien maneja su ingreso?	◯ Sí	◯ No

Otro: ______________________________
(Especifique)

107) NO RELLENAR
⓪ ① ② ③ ④ ⑤ ⑥ ⑦ ⑧ ⑨

108.- Mencione cuántos hijos(as) vivos tiene, si son hombres o mujeres y si viven con usted.

	Edad	Sexo M	Sexo F	¿Vive con usted? Sí	¿Vive con usted? No
Hijo(a) 1	|__|__|	◯	◯	◯	◯
Hijo(a) 2	|__|__|	◯	◯	◯	◯
Hijo(a) 3	|__|__|	◯	◯	◯	◯
Hijo(a) 4	|__|__|	◯	◯	◯	◯
Hijo(a) 5	|__|__|	◯	◯	◯	◯
Hijo(a) 6	|__|__|	◯	◯	◯	◯
Hijo(a) 7	|__|__|	◯	◯	◯	◯

108a) NO RELLENAR
⓪ ① ② ③ ④ ⑤ ⑥ ⑦ ⑧ ⑨
⓪ ① ② ③ ④ ⑤ ⑥ ⑦ ⑧ ⑨

108b) NO RELLENAR
⓪ ① ② ③ ④ ⑤ ⑥ ⑦ ⑧ ⑨
⓪ ① ② ③ ④ ⑤ ⑥ ⑦ ⑧ ⑨

108c) NO RELLENAR
⓪ ① ② ③ ④ ⑤ ⑥ ⑦ ⑧ ⑨
⓪ ① ② ③ ④ ⑤ ⑥ ⑦ ⑧ ⑨

108d) NO RELLENAR
⓪ ① ② ③ ④ ⑤ ⑥ ⑦ ⑧ ⑨
⓪ ① ② ③ ④ ⑤ ⑥ ⑦ ⑧ ⑨

Patrimonio familiar.
Por favor marque las opciones que correspondan mejor a su situación, puede elegir más de una respuesta.

Artículo	Usted posee	Mi pareja posee	Poseemos juntos	Son rentados o de la empresa
Coche	◯	◯	◯	◯
Casa / departamento	◯	◯	◯	◯
Terreno(s)	◯	◯	◯	◯
Locales comerciales	◯	◯	◯	◯
Cuenta bancaria	◯	◯	◯	◯
Otro: ____________ (Especifique)	◯	◯	◯	◯

108e) NO RELLENAR
0 1 2 3 4 5 6 7 8 9
0 1 2 3 4 5 6 7 8 9

108f) NO RELLENAR
0 1 2 3 4 5 6 7 8 9
0 1 2 3 4 5 6 7 8 9

108g) NO RELLENAR
0 1 2 3 4 5 6 7 8 9
0 1 2 3 4 5 6 7 8 9

NO RELLENAR
0 1 2 3 4 5 6 7 8 9

109.- ¿Aproximadamente, cuál es el ingreso mensual de su hogar? (En total, cuánto dinero ganan todas las personas que viven en su hogar, cada mes)

$ |_|_|_|_|_|_|
pesos

109) NO RELLENAR
0 1 2 3 4 5 6 7 8 9
0 1 2 3 4 5 6 7 8 9
0 1 2 3 4 5 6 7 8 9
0 1 2 3 4 5 6 7 8 9
0 1 2 3 4 5 6 7 8 9
0 1 2 3 4 5 6 7 8 9

110.- ¿Cuántas personas dependen de este ingreso cada mes? (Por favor incluya a todas las personas que vivan en su casa y a todas las personas adicionales a las que envían dinero o ayudan económicamente, cada mes)

|_|_|
personas

110) NO RELLENAR
0 1 2 3 4 5 6 7 8 9
0 1 2 3 4 5 6 7 8 9

111.- ¿Tiene usted algún tipo de seguro médico?
◯ Sí ◯ No

→ **¿Tiene usted seguro médico privado?**
◯ Sí ◯ No

112.- ¿Dónde acude usted normalmente cuándo está enfermo(a) o necesita que le den información sobre el cuidado de su salud?

◯ Médico del ISEMYM
◯ Médico del IMSS
◯ Médico privado
◯ Laboratorio privado
◯ Otro: ____________ (Especifique)
◯ Farmacia
◯ Medicina alternativa (acupuntura, homeopatía, curandero)

112) NO RELLENAR
0 1 2 3 4 5 6 7 8 9
0 1 2 3 4 5 6 7 8 9

Salud Mental

113.- ¿Cómo se sintió en la última semana?
Las siguientes afirmaciones describen formas en que la gente actúa o se siente. Por favor indique el número de días en que se sintió así en la última semana.

**La última semana:**	0 días	1-2 días	3-4 días	5-6 días	Todos los días
Le molestaron muchas cosas que generalmente no le molestan	◯	◯	◯	◯	◯
No tenía hambre, no tenía apetito	◯	◯	◯	◯	◯
Sentía que no podía quitarse la tristeza ni con la ayuda de su familia o amigos	◯	◯	◯	◯	◯
Sentía que era tan bueno(a) como los demás	◯	◯	◯	◯	◯
Tenía dificultad para concentrarse en lo que estaba haciendo	◯	◯	◯	◯	◯
Ha tenido la sensación de que algo le va a pasar y no sabe qué es	◯	◯	◯	◯	◯
Se sentía deprimido (a)	◯	◯	◯	◯	◯
Sentía que todo lo que hacía le costaba mucho esfuerzo	◯	◯	◯	◯	◯
Veía el futuro con esperanza	◯	◯	◯	◯	◯
Pensó que su vida era un fracaso	◯	◯	◯	◯	◯
Tenía miedo	◯	◯	◯	◯	◯
Durmió sin descansar	◯	◯	◯	◯	◯
Estaba felíz	◯	◯	◯	◯	◯
Platicó menos de lo normal	◯	◯	◯	◯	◯
Se sentía solo (a)	◯	◯	◯	◯	◯
Sentía que la gente era poco amigable	◯	◯	◯	◯	◯
Disfrutó la vida	◯	◯	◯	◯	◯
Lloraba a ratos	◯	◯	◯	◯	◯
Se sentía triste	◯	◯	◯	◯	◯
Sentía que no le caía bien a otros	◯	◯	◯	◯	◯
No podía “seguir adelante”	◯	◯	◯	◯	◯
Tenía pensamientos sobre la muerte	◯	◯	◯	◯	◯
Sentía que su familia estaría mejor si estuviera muerto(a)	◯	◯	◯	◯	◯
Pensó en matarse	◯	◯	◯	◯	◯

¿Indique si alguna vez ha intentado suicidarse?
◯ Sí ◯ No (pase a la siguiente pregunta)
¿Qué edad tenía la primera vez que lo intentó?
(Edad en años) ⓪ ① ② ③ ④ ⑤ ⑥ ⑦ ⑧ ⑨
⓪ ① ② ③ ④ ⑤ ⑥ ⑦ ⑧ ⑨
¿Cuándo fue la última vez que lo intentó?
◯ Menos de 1 mes ◯ 1 mes a 6 meses ◯ 7 meses a 1 año ◯ Más de 1 año

¿ En la última vez que intentó suicidarse, requirió atención médica?
◯ Sí ◯ No

114.- ¿Qué tan frecuentemente le ocurre lo siguiente en su relación de pareja?	Casi siempre	Frecuente-mente	En ocasiones	Nunca
Mi pareja insiste en mantener su punto de vista	◯	◯	◯	◯
Mi pareja generalmente espera más de mí de lo que él (ella) está dispuesto(a) a dar	◯	◯	◯	◯
Puedo contar con él (ella) para ayudarme con los problemas familiares que requieren más atención	◯	◯	◯	◯
Mi pareja generalmente actúa como si fuera la única persona importante en la familia	◯	◯	◯	◯
Cumplo más con los deseos de mi pareja que lo que él (ella) cumple con los míos	◯	◯	◯	◯
Puedo hablar con él (ella) de cosas que son importantes para mí	◯	◯	◯	◯
Él(ella) es afectuoso(a) conmigo	◯	◯	◯	◯
Mi pareja gasta el dinero prudentemente	◯	◯	◯	◯
Mi pareja tiene un buen salario	◯	◯	◯	◯
Mi compañero(a) es una buena pareja sexual	◯	◯	◯	◯
Mi pareja logra que afloren mis mejores cualidades	◯	◯	◯	◯
Mi pareja me quiere como soy	◯	◯	◯	◯
Mi pareja colabora bien con los quehaceres del hogar	◯	◯	◯	◯
No logro ser yo misma(o) cuando estoy con mi pareja	◯	◯	◯	◯
Mi pareja aprecia la forma en que llevo los quehaceres de la casa	◯	◯	◯	◯
Mi relación de pareja no me permite desarrollarme como para llegar a ser la persona que yo quiero ser	◯	◯	◯	◯

115.- Nos podría indicar qué tan de acuerdo se encuentra con estas observaciones sobre su persona	Muy de acuerdo	De acuerdo	En desacuerdo	Muy en desacuerdo
Soy capaz de hacer las cosas tan bien como otras personas	◯	◯	◯	◯
Siento que no tengo mucho de qué estar orgulloso	◯	◯	◯	◯
Tengo una actitud positiva acerca de mi persona	◯	◯	◯	◯
Me gustaría tenerme más respeto a mi mismo	◯	◯	◯	◯
En general, estoy satisfecho sobre mi mismo	◯	◯	◯	◯
En ocasiones, me siento francamente inútil	◯	◯	◯	◯
Siento que tengo un buen número de cualidades	◯	◯	◯	◯
En ocasiones, siento que soy un bueno para nada	◯	◯	◯	◯
Siento que soy un fracasado	◯	◯	◯	◯
Siento que soy una persona que vale la pena o al menos que valgo tanto como otras	◯	◯	◯	◯
Siento que no hay forma de que yo pueda resolver los problemas que tengo	◯	◯	◯	◯
En ocasiones siento que la vida me lleva sin que yo pueda hacer nada	◯	◯	◯	◯
Tengo poco control en las cosas que me propongo	◯	◯	◯	◯
Con frecuencia me siento incapaz de enfrentar los problemas de la vida	◯	◯	◯	◯
Lo que me pase en el futuro depende en gran medida de mi	◯	◯	◯	◯
Realmente puedo hacer muy poco sobre muchas de las cosas importantes de mi vida	◯	◯	◯	◯

116.- En ocasiones la gente sufre en carne propia o presencia en forma cercana un hecho lamentable (como una agresión o un hecho de violencia, un accidente grave o se ve involucrado en un desastre natural) que le afecta o lo impresiona en forma importante. Nos gustaría saber si usted ha sufrido alguno de estos problemas o ha vivido uno de estos eventos en forma tan cercana que siente que también le hubiera podido suceder a usted.

¿Alguna vez ha sufrido agresión o ha vivido cercanamente una de estas experiencias? ◯ Sí ◯ No

¿Alguna vez lo han asaltado, agredido, herido, o se ha visto involucrado en un accidente o desastre natural en tal forma que pensó que pudiera haber llegado a morir? ◯ Sí ◯ No

Nos gustaría que nos dijera si ha experimentado alguno de los problemas siguientes, en qué año lo sufrió y cómo lo calificaría en términos de gravedad, en una escala del 1 al 5 (considerando que el 1 sería el menos traumático y el 5 el más traumático para usted). ◯ Sí ◯ No

Alguna vez en su vida	Sí	No	¿En qué año? antes de 2007	2007	2008	2009	2010	¿Cómo lo calificaría? (leve 1 - grave 5)
¿Lo(a) han asaltado / agredido violentamente?	◯	◯	◯	◯	◯	◯	◯	① ② ③ ④ ⑤
¿Ha tenido un accidente serio?	◯	◯	◯	◯	◯	◯	◯	① ② ③ ④ ⑤
¿Ha visto cómo es que muere cerca de usted otra persona por un ataque de violencia?	◯	◯	◯	◯	◯	◯	◯	① ② ③ ④ ⑤
¿Ha sido atacado(a) sexualmente?	◯	◯	◯	◯	◯	◯	◯	① ② ③ ④ ⑤
¿Ha sufrido el suicidio de un familiar cercano?	◯	◯	◯	◯	◯	◯	◯	① ② ③ ④ ⑤

117.- En caso de haber sufrido algún tipo de agresión por parte de su pareja nos gustaría saber cómo se siente ahora sobre este problema. Para esto responda las siguientes preguntas:	Siempre	Casi siempre	Frecuentemente	En ocasiones	Casi nunca	Nunca
Se siente asustado(a), temeroso(a), o molesto(a) cuando piensa en lo que ocurrió	○	○	○	○	○	○
Le vuelve a la mente, esto es, recuerda imágenes, sonidos, etc. de lo que ocurrió	○	○	○	○	○	○
Los recuerdos de lo que ocurrió le vuelven a la mente cuando usted no quiere	○	○	○	○	○	○
Tiene sueños o pesadillas sobre lo que ocurrió	○	○	○	○	○	○
Piensa que algo similar a lo que le ocurrió le pasará de nuevo	○	○	○	○	○	○
Se siente más solo(a) desde que ocurrió este problema	○	○	○	○	○	○
Se siente tan temeroso(a) por lo que le ocurrió que ya no quiere pensar cómo se siente	○	○	○	○	○	○
Se siente tan temeroso(a) o nervioso(a) desde que le ocurrió que ya no quiere ni hablar o llorar	○	○	○	○	○	○
Se siente tan inestable y nervioso(a) desde que le ocurrió este problema	○	○	○	○	○	○
Duerme bien desde que le ocurrió este problema	○	○	○	○	○	○
Se siente mal o culpable porque no hizo lo que quiso o debió hacer para evitar que le ocurriera este problema	○	○	○	○	○	○
Puede usted poner atención o concentrarse como antes de que le ocurriera este problema	○	○	○	○	○	○
Quiere estar lejos de las cosas o lugares que le recuerdan lo que le ocurrió	○	○	○	○	○	○
Se pone tenso(a) o molesto(a) cuando algo le recuerda o le hace pensar en lo que le ocurrió	○	○	○	○	○	○
Esta teniendo molestias como dolor de estómago o dolor de cabeza más frecuentemente que antes que le ocurriera este problema	○	○	○	○	○	○

Apoyo Social y Calidad de Vida

En la etapa previa del estudio de Cohorte de Trabajadores del IMSS, UAEM e INSP se incluyeron algunas preguntas que nos han ayudado a conocer cómo es la percepción que cada uno de los participantes tiene sobre su propia salud y determinar algunos aspectos relacionados con la calidad de vida. Los resultados nos señalan que alrededor del 12% consideró que estaba en condiciones óptimas de salud y menos del 2% percibió que su estado de salud era el peor imaginable. Así mismo, cerca del 10% de las mujeres y el 6% de los hombres consideraron que su estado de salud había empeorado durante los últimos 12 meses. Esta valiosa información nos permite plantear varias preguntas que pueden estar relacionadas con estos resultados y proponer una medición mas integral sobre la calidad de vida. A continuación le haremos una serie de preguntas con la finalidad de conocer la percepción que tiene usted sobre su calidad de vida y su estado de salud en general. Esta información nos ayudará a conocer de qué forma sus condiciones generales de salud están relacionadas con su desempeño cotidiano y su bienestar individual.

A continuación encontrará una serie de preguntas sobre la ayuda con la que usted puede disponer durante diferentes momentos de la vida. Al leer la pregunta piense si algún familiar o amigo cercano, le ayudarían.

118.- Alrededor de cuántos parientes allegados o amigos cercanos tiene usted? (personas con las que pueda y se sienta cómodo (a) para hablar de sus cosas).

Parientes allegados |__|__| ⓪ ① ② ③ ④ ⑤ ⑥ ⑦ ⑧ ⑨ / ⓪ ① ② ③ ④ ⑤ ⑥ ⑦ ⑧ ⑨

Amigos cercanos |__|__| ⓪ ① ② ③ ④ ⑤ ⑥ ⑦ ⑧ ⑨ / ⓪ ① ② ③ ④ ⑤ ⑥ ⑦ ⑧ ⑨

La gente algunas veces busca otras compañías, asistencia u otro tipo de ayuda.

Registre si es un familiar, amigo o ambos, quiénes le ayudan en las diferentes situaciones planteadas.

119.- ¿Qué tan frecuentemente puede contar con alguien...	Nunca	Casi nunca	Algunas veces	Casi siempre	Siempre	¿Quién le proporciona ayuda? a)Familiar	b)Amigo(a)
Que le proporcione ayuda si estuviera confinado(a) a la cama?	○	○	○	○	○	○	○
Que lo(a) escucha cuando tiene necesidad de hablar?	○	○	○	○	○	○	○
Que le dé un buen consejo en un momento de crisis?	○	○	○	○	○	○	○
Que lo(a) lleve al doctor si usted lo necesita?	○	○	○	○	○	○	○
Que le dé amor y afecto?	○	○	○	○	○	○	○
Con quién pasar un buen rato?	○	○	○	○	○	○	○
Que le brinde información para ayudarle a entender una situación en particular?	○	○	○	○	○	○	○
En quién confiar para hablar sobre usted mismo(a) o sus problemas?	○	○	○	○	○	○	○
Que lo(a) abrace?	○	○	○	○	○	○	○
Con quién estar junto(a) para relajarse?	○	○	○	○	○	○	○
Que le prepare su comida si usted no pudiera hacerlo?	○	○	○	○	○	○	○
Que le aconseje realmente?	○	○	○	○	○	○	○
Que haga cosas que le ayuden a despejar la mente?	○	○	○	○	○	○	○
Que le ayude con los quehaceres cotidianos si usted estuviera enfermo (a)?	○	○	○	○	○	○	○
Con quién compartir sus preocupaciones y miedos?	○	○	○	○	○	○	○
Que le dé sugerencias sobre cómo enfrentar un problema personal?	○	○	○	○	○	○	○
Para hacer algo que usted disfrute?	○	○	○	○	○	○	○
Que entienda sus problemas?	○	○	○	○	○	○	○
Que le quiera y que le haga sentir querido (a)?	○	○	○	○	○	○	○

120.- ¿En general diría que su salud es:

◯ Excelente ◯ Muy Buena ◯ Buena ◯ Regular ◯ Mala ◯ No sabe

**121.- Las siguientes preguntas se refieren a las actividades que usted podría hacer en un día normal ¿Su estado de salud actual lo limita para...**	Le limita mucho	Le limita poco	No le limita en absoluto
Hacer actividades vigorosas, tales como: correr, levantar objetos pesados, participar en deportes intensos	◯	◯	◯
Hacer actividades moderadas, tales como: mover una mesa, barrer, trapear, lavar, o jugar béisbol o fútbol	◯	◯	◯
Levantar y/o llevar las compras del mercado	◯	◯	◯
Subir un piso por las escaleras	◯	◯	◯
Doblarse, arrodillarse o agacharse	◯	◯	◯
Caminar más de diez cuadras	◯	◯	◯
Caminar varias cuadras	◯	◯	◯
Bañarse o vestirse	◯	◯	◯

**Durante el último mes: ¿Ha tenido usted alguno de los siguientes problemas con el trabajo u otras actividades diarias normales a causa de su salud física?**	Sí	No
¿Ha reducido el tiempo que dedicaba al trabajo u otras actividades?	◯	◯
¿Ha logrado hacer menos de lo que le hubiera gustado?	◯	◯
¿Ha tenido limitaciones en cuanto al tipo de trabajo u otras actividades?	◯	◯
¿Ha tenido dificultades para realizar su trabajo u otras actividades?	◯	◯

122.- Durante el último mes:
¿En qué medida su salud o sus problemas emocionales han dificultado sus actividades sociales normales con la familia, amigos, vecinos o grupos?

◯ Nada ◯ Más o menos ◯ Demasiado
◯ Un poco ◯ Mucho

¿Cuánto dolor físico ha tenido durante el último mes?

◯ Ninguno ◯ Poco ◯ Severo
◯ Muy poco ◯ Moderado ◯ Muy severo

Durante el último mes ¿Qué tanto este dolor ha dificultado su trabajo normal?
(incluyen tanto el trabajo fuera de casa como los quehaceres domésticos)

◯ Nada ◯ Más o menos ◯ Demasiado
◯ Un poco ◯ Mucho

**123.- ¿Cómo se ha sentido durante el último mes?** Por cada pregunta, por favor de la respuesta que más se acerca a la manera como se ha sentido usted:	Siempre	Casi siempre	Muchas veces	Algunas veces	Casi nunca	Nunca
Se ha sentido lleno(a) de vida	◯	◯	◯	◯	◯	◯
Se ha sentido muy nervioso(a)	◯	◯	◯	◯	◯	◯
Se ha sentido tan decaído(a) de ánimo que nada podría alentarlo(a)	◯	◯	◯	◯	◯	◯
Se ha sentido tranquilo(a) y sosegado(a)	◯	◯	◯	◯	◯	◯
Ha tenido mucha energía	◯	◯	◯	◯	◯	◯
Se ha sentido desanimado(a) o triste	◯	◯	◯	◯	◯	◯
Se ha sentido agotado(a)	◯	◯	◯	◯	◯	◯
Se ha sentido feliz	◯	◯	◯	◯	◯	◯
Se ha sentido cansado(a)	◯	◯	◯	◯	◯	◯
Su salud o sus problemas emocionales han dificultado sus actividades sociales (como visitar amigos o parientes)	◯	◯	◯	◯	◯	◯

**124.- ¿Qué tan de acuerdo está con las siguientes frases?**	Totalmente de acuerdo	Mediamente de acuerdo	No sé	Mediamente en desacuerdo	Totalmente en desacuerdo
Le parece que se enferma un poco más fácilmente que otras personas	◯	◯	◯	◯	◯
Le parece que tiene tan buena salud como cualquiera que conoce	◯	◯	◯	◯	◯
Cree que su salud va a empeorar	◯	◯	◯	◯	◯
Cree que su salud es excelente	◯	◯	◯	◯	◯

125.- Elija la calificación que mejor describa su estado de salud al día de hoy, tomando en cuenta que "0" es el peor estado de salud imaginable y "100" el mejor estado de salud imaginable.

Peor estado de salud — Mejor estado de salud

◯ 0 ◯ 10 ◯ 20 ◯ 30 ◯ 40 ◯ 50 ◯ 60 ◯ 70 ◯ 80 ◯ 90 ◯ 100

Comparado con su estado de salud durante los últimos 12 meses, su salud hoy es: ◯ Mejor ◯ Igual ◯ Peor

126.- En su trabajo tiene contacto físico con enfermos o con desechos biológicos, como jeringas o agujas usadas, equipo punzocortante, bisturí, sangre, etc.?

◯ Sí ◯ No ◯ No trabajo

(Pase a la pregunta 134)

127.- ¿Con qué frecuencia tiene contacto físico con enfermos o con desechos biológicos?

◯ Todos los días ◯ Una vez por semana
◯ Una vez al mes ◯ Más de una vez al mes

128.- ¿Con qué frecuencia se lava las manos antes y después de tener contacto con enfermos o con desechos biológicos?

◯ Siempre ◯ A veces ◯ Nunca

129.- ¿Alguna vez han estado en contacto su piel, ojos o boca con la sangre o los fluidos corporales de un enfermo?

◯ Sí ◯ No

130.- ¿En su trabajo, con qué frecuencia maneja los siguientes productos biológicos usando guantes?

130) NO RELLENAR
⓪ ① ② ③ ④ ⑤ ⑥ ⑦ ⑧ ⑨
⓪ ① ② ③ ④ ⑤ ⑥ ⑦ ⑧ ⑨

	Casi siempre	A veces	Casi nunca	Nunca
◯ Sangre	◯	◯	◯	◯
◯ Heces	◯	◯	◯	◯
◯ Orina	◯	◯	◯	◯
◯ Mucosa	◯	◯	◯	◯
◯ Otro: ________ (Especifique)	◯	◯	◯	◯

131.- ¿En los últimos 12 meses, ha recibido capacitación en las siguientes áreas? (puede responder más de una opción)

◯ No he recibido capacitación en los últimos 12 meses
◯ Lavado de manos
◯ Uso de guantes
◯ Manejo apropiado de basura y de desechos biológicos
◯ Manejo apropiado de heridas en la piel
◯ Desecho apropiado de agujas y jeringas
◯ Desecho apropiado de desechos infecciosos
◯ Otro: (Especifique) ________________

131) NO RELLENAR
⓪ ① ② ③ ④ ⑤ ⑥ ⑦ ⑧ ⑨
⓪ ① ② ③ ④ ⑤ ⑥ ⑦ ⑧ ⑨

132.- ¿Alguna vez se ha picado o cortado la piel con algún tipo de equipo punzocortante de tal manera que le salió sangre, mientras estaba en su trabajo?

◯ Sí ◯ No (pase a la siguiente pregunta)

¿Cuántas veces se ha picado o cortado la piel en el trabajo en los últimos 12 meses?

◯ Ninguna ◯ 3-4 veces ◯ más de 10 veces
◯ 1-2 veces ◯ 5-10 veces

¿Hace cuánto tiempo fue la última vez que se cortó o pinchó la piel en su trabajo?

◯ Este mes ◯ Hace 2 años
◯ Hace 2-5 meses ◯ Hace 3-5 años
◯ Hace 6-11 meses ◯ Hace más de 5 años
◯ Hace un año ◯ Nunca me he cortado o pinchado

¿Cómo se cortó o pinchó la piel en el trabajo, la última vez que esto ocurrió?

◯ Me piqué mientras estaba inyectando a un paciente.
◯ Me piqué mientras estaba tomando una muestra de sangre de un paciente.
◯ Me piqué mientras estaba insertando o quitando un suero.
◯ Me piqué con una aguja mientras estaba intentando ponerle su capuchón.
◯ Me piqué con una aguja mientras estaba tirándola a la basura.
◯ Me piqué con una aguja que no fue desechada correctamente.
◯ Un médico (u otro trabajador de salud) me picó por accidente.
◯ Me piqué con una aguja que estaba en las sábanas de la cama de un paciente.
◯ Me piqué con la aguja que estaba en una bolsa de basura
◯ Me corté mientras estaba operando un paciente
◯ Otro: (Especifique) ________________

132) NO RELLENAR
⓪ ① ② ③ ④ ⑤ ⑥ ⑦ ⑧ ⑨
⓪ ① ② ③ ④ ⑤ ⑥ ⑦ ⑧ ⑨

133.- ¿Ha recibido alguna vez una transfusión de sangre?

◯ Sí ◯ No ◯ No sé

¿En qué año recibió su primera transfusión de sangre?

|_|_|_|_| año

◯ No sé

133) NO RELLENAR
⓪ ① ② ③ ④ ⑤ ⑥ ⑦ ⑧ ⑨
⓪ ① ② ③ ④ ⑤ ⑥ ⑦ ⑧ ⑨
⓪ ① ② ③ ④ ⑤ ⑥ ⑦ ⑧ ⑨
⓪ ① ② ③ ④ ⑤ ⑥ ⑦ ⑧ ⑨

134.- ¿Alguna vez ha usado drogas intravenosas?

◯ Sí ◯ No

¿Alguna vez ha compartido agujas al usar drogas intravenosas?

◯ Sí ◯ No

135.- Actualmente o en el pasado: (Puede responder más de una opción)

◯ Me he realizado un tatuaje
◯ He estado expuesto a agujas de acupuntura
◯ Me he realizado perforaciones en el cuerpo
◯ He usado cocaína u otras drogas intranasales
◯ He compartido un rastrillo, cepillo de dientes u otra cosa que pudiera tener sangre infectada
◯ He estado en el ejército
◯ He cambiado de pareja sexual con frecuencia
◯ He tenido relaciones sexuales con trabajadores(as) de sexo comercial
◯ He estado en prisión
◯ He tenido problemas de alcoholismo
◯ Mi piel, ojos o boca han estado en contacto con la sangre o los fluídos corporales de otra persona
◯ Me he realizado alguna cirugía dental o algún otro procedimiento dental
◯ Me he realizado una cirugía

136.- ¿Alguna vez ha tenido relaciones sexuales?

◯ Sí ◯ No

(Pase a la pregunta 143)

137.- ¿A qué edad tuvo relaciones sexuales por primera vez?

|__|__| años

⓪ ① ② ③ ④ ⑤ ⑥ ⑦ ⑧ ⑨
⓪ ① ② ③ ④ ⑤ ⑥ ⑦ ⑧ ⑨

138.- ¿Con cuántas personas ha tenido relaciones sexuales en toda su vida?

|__|__|__| parejas

⓪ ① ② ③ ④ ⑤ ⑥ ⑦ ⑧ ⑨
⓪ ① ② ③ ④ ⑤ ⑥ ⑦ ⑧ ⑨
⓪ ① ② ③ ④ ⑤ ⑥ ⑦ ⑧ ⑨

139.- ¿Cuántas parejas sexuales masculinas ha tenido en toda su vida?

|__|__| parejas

⓪ ① ② ③ ④ ⑤ ⑥ ⑦ ⑧ ⑨
⓪ ① ② ③ ④ ⑤ ⑥ ⑦ ⑧ ⑨

140.- ¿Cuántas parejas sexuales femeninas ha tenido en toda su vida?

|__|__| parejas

⓪ ① ② ③ ④ ⑤ ⑥ ⑦ ⑧ ⑨
⓪ ① ② ③ ④ ⑤ ⑥ ⑦ ⑧ ⑨

141.- ¿Alguna vez ha recibido tratamiento médico por tener una enfermedad de transmisión sexual?

◯ Sí ◯ No ◯ No sé

142.- ¿Con qué frecuencia utiliza el condón o preservativo cuando tiene relaciones sexuales?

◯ Siempre ◯ A veces ◯ Nunca
◯ Casi siempre ◯ No muy seguido

La siguiente sección se refiere al consumo de bebidas alcohólicas. Para todos los casos considere que una bebida alcohólica equivale a una lata o a una botella de cerveza, una copa de vino, una bebida mezclada (una cuba, un bloody mary, un desarmador, etc.), un vaso de pulque, o un caballito de tequila, whisky, ron, etc.

143.- ¿En toda su vida ha tomado por lo menos 12 bebidas alcohólicas de cualquier tipo?

◯ Sí ◯ No (Pase a la pregunta 151)

144.- ¿A qué edad empezó a beber alcohol?

|__|__| años

⓪ ① ② ③ ④ ⑤ ⑥ ⑦ ⑧ ⑨
⓪ ① ② ③ ④ ⑤ ⑥ ⑦ ⑧ ⑨

145.- ¿Actualmente bebe alcohol?

◯ No ◯ Sí

¿A qué edad dejó de beber alcohol?

|__|__| años

⓪ ① ② ③ ④ ⑤ ⑥ ⑦ ⑧ ⑨
⓪ ① ② ③ ④ ⑤ ⑥ ⑦ ⑧ ⑨

146.- ¿Cuando bebía alcohol, cuántos días a la semana o al mes tomaba por lo menos una bebida alcohólica?

|__|__| días a la semana

⓪ ① ② ③ ④ ⑤ ⑥ ⑦ ⑧ ⑨

|__|__| días al mes

⓪ ① ② ③
⓪ ① ② ③ ④ ⑤ ⑥ ⑦ ⑧ ⑨

◯ Menos de una bebida alcohólica al mes ◯ No sé

147.- ¿La última vez que tomó algún tipo de bebida alcohólica, cuántas copas se tomó?

◯ Una copa ◯ Cuatro copas ◯ Siete copas ◯ Más de 15 copas
◯ Dos copas ◯ Cinco copas ◯ 6 a 10 copas
◯ Tres copas ◯ Seis copas ◯ 11 a 15 copas

148.- ¿Durante los últimos 30 días cuántos días a la semana o al mes tomó por lo menos una bebida alcohólica?

|__|__| días a la semana

⓪ ① ② ③ ④ ⑤ ⑥ ⑦ ⑧ ⑨

|__|__| días al mes

⓪ ① ② ③
⓪ ① ② ③ ④ ⑤ ⑥ ⑦ ⑧ ⑨

◯ No bebí en el último mes ◯ No sé

149.- ¿En los días que bebió alcohol durante los últimos 30 días, aproximadamente cuántas copas se tomó?

|__|__| número de copas

⓪ ① ② ③ ④ ⑤ ⑥ ⑦ ⑧ ⑨
⓪ ① ② ③ ④ ⑤ ⑥ ⑦ ⑧ ⑨
◯ No sé

150.- ¿Considerando todas las bebidas alcohólicas, cuántos días tomó 5 ó más bebidas alcohólicas seguidas, o sea en unas cuántas horas, durante los últimos 30 días?

|__|__| días

⓪ ① ② ③
⓪ ① ② ③ ④ ⑤ ⑥ ⑦ ⑧ ⑨

◯ Ningún día ◯ No sé

151.- ¿Se ha realizado una prueba de sangre para saber si ha estado en contacto con el virus de la hepatitis B?

◯ Sí ◯ No ◯ No sé

¿Cuál fue el resultado de su prueba de hepatitis B?

◯ Tengo una infección de hepatitis B
◯ Estuve infectado con hepatitis B en el pasado, pero ahora soy inmune al virus
◯ Negativo, que no he tenido hepatitis B
◯ No sé

152.- ¿Ha recibido la vacuna para prevenir la infección con hepatitis B?

◯ Sí ◯ No ◯ No sé

¿Cuántas dosis (vacunas) ha recibido para prevenir la infección por hepatitis B?

◯ Una ◯ Dos ◯ Tres ◯ No sé

¿En qué año recibió la última dosis (vacuna) de hepatitis B?

|__|__|__|__| Año

⓪ ① ② ③
⓪ ① ② ③ ④ ⑤ ⑥ ⑦ ⑧ ⑨
⓪ ① ② ③ ④ ⑤ ⑥ ⑦ ⑧ ⑨
⓪ ① ② ③ ④ ⑤ ⑥ ⑦ ⑧ ⑨

153.- ¿Se ha realizado una prueba de sangre para saber si ha estado en contacto con el virus de la hepatitis C?

◯ Sí ◯ No ◯ No sé

¿Cuál fue el resultado de su prueba de hepatitis C?

◯ Tengo una infección de hepatitis C
◯ Estuve infectado con hepatitis C en el pasado, pero ahora soy inmune al virus
◯ Negativo, que no he tenido hepatitis C
◯ No sé

Salud Sexual y Reproductiva

Existen muchos problemas de salud de la mujer como el cáncer cervicouterino, el cáncer mamario, la menopausia y la osteoporosis, entre otros, que tienen relación con la salud reproductiva de la mujer, en mayor o menor grado. Sin embargo, aún existen muchos vacios en el conocimiento sobre la mejor forma de prevenir estos problemas por lo que continúan siendo una necesidad de investigación.

El inicio de la menstruación, el inicio de las relaciones sexuales y sus características, el número de embarazos, el espaciamiento de los mismos, el uso de métodos de planificación familiar y la presentación de la menopausia, son todos aspectos que forman parte de la salud sexual y reproductiva de las mujeres.

Los datos que nos proporciona cada una de las mujeres que participa en este estudio son de gran utilidad para conocer mejor los problemas relacionados con la salud sexual y reproductiva de las mujeres en México. Nuestros resultados permitirán proponer estrategias de atención que contribuyan a mejorar la salud de las mujeres.

Recuerde que la información que nos proporciona en este cuestionario es estrictamente confidencial, la identidad de los pacientes es removida de los datos y por lo tanto para los investigadores esta información es anónima.

154.- ¿Cuántos años tenía cuando tuvo su primera menstruación (regla)?

|__|__| años

⓪ ① ② ③ ④ ⑤ ⑥ ⑦ ⑧ ⑨
⓪ ① ② ③ ④ ⑤ ⑥ ⑦ ⑧ ⑨

155.- ¿Después de que inició su regla, cuánto tiempo pasó para que fuera regular?

- ○ Fue de inmediato
- ○ De 1 a 2 años
- ○ 3 años ó más → Si recuerda los meses exactos por favor indique |__|__| meses
- ○ Siempre ha sido irregular
- ○ No recuerdo

⓪ ① ② ③ ④ ⑤ ⑥ ⑦ ⑧ ⑨
⓪ ① ② ③ ④ ⑤ ⑥ ⑦ ⑧ ⑨

156.- ¿Cuando usted tenía entre 20 y 35 años y no estaba embarazada ni tomaba anticonceptivos, por lo general, cómo se presentaba su regla? (Si tiene más de 55 años pase a la siguiente pregunta)

○ Era irregular ○ Era regular

→ **¿Cada cuántos días le venía su regla a esta edad?**

|__|__| días

⓪ ① ② ③ ④ ⑤ ⑥ ⑦ ⑧ ⑨
⓪ ① ② ③ ④ ⑤ ⑥ ⑦ ⑧ ⑨

¿Cuántos días duraba el sangrado generalmente?

|__|__| días

⓪ ① ② ③ ④ ⑤ ⑥ ⑦ ⑧ ⑨
⓪ ① ② ③ ④ ⑤ ⑥ ⑦ ⑧ ⑨

157.- ¿A qué edad se le empezaron a desarrollar sus pechos?

|__|__| años

⓪ ① ② ③
⓪ ① ② ③ ④ ⑤ ⑥ ⑦ ⑧ ⑨

158.- ¿Si todavía regla, cada cuándo le viene su regla?
(anotar el número de días entre el primer día de sangrado de una menstruación y el primer día de sangrado de la siguiente menstruación)

- ○ Cada 26 días o menos
- ○ De 27 a 32 días
- ○ De 32 a 40 días
- ○ De 41 a 60 días
- ○ Más de 61 días → Si recuerda los días exactos por favor indique el número de días |__|__| meses
- ○ Es variable

⓪ ① ② ③ ④ ⑤ ⑥ ⑦ ⑧ ⑨
⓪ ① ② ③ ④ ⑤ ⑥ ⑦ ⑧ ⑨

159.- ¿En alguna ocasión ha estado sin reglar por más de dos meses, sin estar embarazada ni tomando anticonceptivos?

○ No (pase a la siguiente pregunta)
○ Sí → ¿Cuántas ocasiones le ha sucedido esto? |__|__| veces

⓪ ① ② ③ ④ ⑤ ⑥ ⑦ ⑧ ⑨
⓪ ① ② ③ ④ ⑤ ⑥ ⑦ ⑧ ⑨

¿Cuál es el tiempo máximo que ha permanecido sin reglar (sin estar embarazada ni tomando anticonceptivos)?

|__|__| meses

⓪ ① ② ③ ④ ⑤ ⑥ ⑦ ⑧ ⑨
⓪ ① ② ③ ④ ⑤ ⑥ ⑦ ⑧ ⑨

¿Qué edad tenía cuando le ocurrió esto en la última ocasión?

|__|__| años

⓪ ① ② ③ ④ ⑤ ⑥ ⑦ ⑧ ⑨
⓪ ① ② ③ ④ ⑤ ⑥ ⑦ ⑧ ⑨

160.- ¿Alguna vez ha tenido relaciones sexuales?

○ Sí ○ No (pase a la pregunta 174)

¿A qué edad tuvo usted su primera relación sexual?

⓪ ① ② ③ ④
⓪ ① ② ③ ④ ⑤ ⑥ ⑦ ⑧ ⑨

|__|__| años

¿En toda su vida, con cuántas parejas ha tenido relaciones sexuales?

⓪ ① ② ③ ④ ⑤ ⑥ ⑦ ⑧ ⑨ ó más |__|__| parejas

161.- Para cada pareja por favor marque el círculo que indique la edad de inicio y marque también los círculos de las edades hasta las que continuó teniendo relaciones con esta misma pareja. Para las parejas ocasionales con las que no volvió a tener relaciones (por ejemplo, para un encuentro de una sola vez sólo hay que marcar el círculo de la edad en que éste ocurrió).

Edades	1a.	2a.	3a.	4a.	5a.	6a.	7a.	8a.	9a.	10a.	11a.	12a.	13a.	14a.	15a.	16a.	17a.	18a.	19a.	20a.
menos de 18 años	○	○	○	○	○	○	○	○	○	○	○	○	○	○	○	○	○	○	○	○
18 - 22 años	○	○	○	○	○	○	○	○	○	○	○	○	○	○	○	○	○	○	○	○
23 - 27 años	○	○	○	○	○	○	○	○	○	○	○	○	○	○	○	○	○	○	○	○
28 - 32 años	○	○	○	○	○	○	○	○	○	○	○	○	○	○	○	○	○	○	○	○
33 - 37 años	○	○	○	○	○	○	○	○	○	○	○	○	○	○	○	○	○	○	○	○
38 - 42 años	○	○	○	○	○	○	○	○	○	○	○	○	○	○	○	○	○	○	○	○
43 - 47 años	○	○	○	○	○	○	○	○	○	○	○	○	○	○	○	○	○	○	○	○
48 - 52 años	○	○	○	○	○	○	○	○	○	○	○	○	○	○	○	○	○	○	○	○
53 - 57 años	○	○	○	○	○	○	○	○	○	○	○	○	○	○	○	○	○	○	○	○
58 años o más	○	○	○	○	○	○	○	○	○	○	○	○	○	○	○	○	○	○	○	○

162.- ¿Alguna vez ha estado embarazada?

◯ Sí ◯ No

→ ¿Qué edad tenía en su primer embarazo?

0 1 2 3 4
0 1 2 3 4 5 6 7 8 9

¿Cuántas veces ha estado embarazada? (Sin incluir su embarazo actual en caso de estarlo, no olvide incluir los abortos)

0 1 2 3 4 5 6 7 8 9 10 ó más

¿En alguno de sus embarazos ha tenido un hijo con peso igual o mayor de 4 kg. al nacer?

◯ Sí ◯ No

→ ¿Qué edad tenía en su embarazo?

0 1 2 3 4
0 1 2 3 4 5 6 7 8 9

163.- ¿En alguno de sus embarazos ha tenido un hijo con peso menor de 2.5 kg al nacer?

◯ Sí ◯ No

¿Cuántos? ←

0 1 2 3 4 5 6 7 8 9 10 ó mas

¿Cómo han terminado sus embarazos?

	¿Cuántos?
Parto vaginal	0 1 2 3 4 5 6 7 8 9 10 ó más
Cesárea	0 1 2 3 4 5 6 7 8 9 10 ó más
Aborto	0 1 2 3 4 5 6 7 8 9 10 ó más

¿Número de hijos nacidos vivos?

0 1 2 3 4 5 6 7 8 9 10 ó más

¿Le dio pecho a su(s) hijo(s)? ◯ Sí ◯ No (pase a la siguiente pregunta)

¿Cuántos meses le(s) dio pecho?

Hijo 1	0 1 2 3 4 5 6 7 8 9 10 11 12 13 ó más
Hijo 2	0 1 2 3 4 5 6 7 8 9 10 11 12 13 ó más
Hijo 3	0 1 2 3 4 5 6 7 8 9 10 11 12 13 ó más
Hijo 4	0 1 2 3 4 5 6 7 8 9 10 11 12 13 ó más
Hijo 5	0 1 2 3 4 5 6 7 8 9 10 11 12 13 ó más
Hijo 6	0 1 2 3 4 5 6 7 8 9 10 11 12 13 ó más
Hijo 7	0 1 2 3 4 5 6 7 8 9 10 11 12 13 ó más
Hijo 8	0 1 2 3 4 5 6 7 8 9 10 11 12 13 ó más

164.- ¿Por qué no se ha embarazado?

◯ He evitado tener hijos
◯ Aún no inicio una relación de pareja
◯ Porque soy estéril
◯ Otra razón ____________________ (Especifique)
◯ Mi compañero es estéril

164) NO RELLENAR
0 1 2 3 4 5 6 7 8 9
0 1 2 3 4 5 6 7 8 9

165.- Marque si ha utilizado algunos de lo siguientes métodos para evitar el embarazo:
(sólo marque aquel que sí ha utilizado)

165) NO RELLENAR
0 1 2 3 4 5 6 7 8 9
0 1 2 3 4 5 6 7 8 9

Métodos	Sí	¿Cuántos años lo utilizó?
Dispositivo intrauterino	◯	0 1 2 3 4 5 6 7 8 9 10 11 12 13 14 15 16 17 18 19 20
Condón o preservativo	◯	0 1 2 3 4 5 6 7 8 9 10 11 12 13 14 15 16 17 18 19 20
Óvulos vaginales	◯	0 1 2 3 4 5 6 7 8 9 10 11 12 13 14 15 16 17 18 19 20
Pastillas	◯	0 1 2 3 4 5 6 7 8 9 10 11 12 13 14 15 16 17 18 19 20
Calendario o ritmo	◯	0 1 2 3 4 5 6 7 8 9 10 11 12 13 14 15 16 17 18 19 20
Salpingoclasia/ligada	◯	0 1 2 3 4 5 6 7 8 9 10 11 12 13 14 15 16 17 18 19 20
Mi esposo se hizo vasectomía	◯	0 1 2 3 4 5 6 7 8 9 10 11 12 13 14 15 16 17 18 19 20
Otro: ____________ (Especifique)	◯	0 1 2 3 4 5 6 7 8 9 10 11 12 13 14 15 16 17 18 19 20

166.- Si ha utilizado o utiliza actualmente anticonceptivos (pastillas o inyecciones) para evitar el embarazo: especifique la marca de anticonceptivos (pastillas o inyecciones) que utilizó anteriormente, o utiliza actualmente. Anote también el número total de meses de utilización de cada uno de ellos. En caso de estarlo usando actualmente, no olvide incluir este tiempo en la suma total de meses.

Nombre y marca del medicamento	TIPO: Oral	TIPO: Inyectable	(Anote el número total de meses, no olvide que por cada uno deberá contar 12 meses, ejemplo: 5 años equivalen a 60 meses. 2 años y 5 mese equivalen 29 meses)	
	◯	◯	0 1 2 3 4 5 6 7 8 9<br>0 1 2 3 4 5 6 7 8 9	0 1 2 3 4 5 6 7 8 9<br>0 1 2 3 4 5 6 7 8 9
	◯	◯	0 1 2 3 4 5 6 7 8 9<br>0 1 2 3 4 5 6 7 8 9	0 1 2 3 4 5 6 7 8 9<br>0 1 2 3 4 5 6 7 8 9
	◯	◯	0 1 2 3 4 5 6 7 8 9<br>0 1 2 3 4 5 6 7 8 9	0 1 2 3 4 5 6 7 8 9<br>0 1 2 3 4 5 6 7 8 9
◯ No recuerdo la marca, pero sí utilicé	◯	◯	0 1 2 3 4 5 6 7 8 9<br>0 1 2 3 4 5 6 7 8 9	0 1 2 3 4 5 6 7 8 9<br>0 1 2 3 4 5 6 7 8 9

166a) NO RELLENAR
0 1 2 3 4 5 6 7 8 9
0 1 2 3 4 5 6 7 8 9

166b) NO RELLENAR
0 1 2 3 4 5 6 7 8 9
0 1 2 3 4 5 6 7 8 9

166c) NO RELLENAR
0 1 2 3 4 5 6 7 8 9
0 1 2 3 4 5 6 7 8 9

167.- ¿Actualmente usted regla? ◯ Sí ◯ No (pase a la pregunta 170)

Escriba la fecha de su última regla (el día que empezó):

día: ⓪ ① ② ③ ④ ⑤ ⑥ ⑦ ⑧ ⑨ / ⓪ ① ② ③ ④ ⑤ ⑥ ⑦ ⑧ ⑨

meses: ⓪ ① ② ③ ④ ⑤ ⑥ ⑦ ⑧ ⑨ / ⓪ ① ② ③ ④ ⑤ ⑥ ⑦ ⑧ ⑨

¿Cuántas veces ha menstruado en los últimos 12 meses?

veces: ⓪ ① ② ③ ④ ⑤ ⑥ ⑦ ⑧ ⑨ / ⓪ ① ② ③ ④ ⑤ ⑥ ⑦ ⑧ ⑨

Durante los últimos 12 meses el número de días de sangrado de su regla ha sido:

◯ Más días ◯ Menos días ◯ Se ha vuelto variable ◯ Igual que siempre

¿Desde hace cuánto ha presentado estos cambios?

◯ 2 meses ◯ 3 meses ◯ 4 meses
◯ 5 meses ◯ + 6 meses

168.- Durante los últimos 5 años la cantidad de sangrado de su regla ha sido:

◯ Mayor cantidad de sangrado ◯ Menor cantidad de sangrado ◯ Igual que siempre

¿Desde hace cuánto ha presentado estos cambios?

◯ 2 meses ◯ 3 meses ◯ 4 meses
◯ 5 meses ◯ + 6 meses

La duración entre una y otra menstruación (es decir, el número de días entre el primer día de una menstruación y el primer día de la siguiente menstruación) cómo ha sido durante los últimos 5 años?

días: ⓪ ① ② ③ ④ / ⓪ ① ② ③ ④ ⑤ ⑥ ⑦ ⑧ ⑨

¿Desde hace cuánto ha presentado estos cambios?

◯ 2 meses ◯ 3 meses ◯ 4 meses
◯ 5 meses ◯ + 6 meses

169.- ¿Por qué dejo de reglar?

◯ Por menopausia ◯ Por cirugía → 170

¿Le quitaron a usted sus ovarios?

◯ No ◯ Sí → ◯ uno ◯ los dos ◯ no sabe

¿Por qué motivo?
◯ Por quistes
◯ No sabe
◯ Por embarazo ectópico
◯ Otro: (Especifique) ____________________

169a) NO RELLENAR
⓪ ① ② ③ ④ ⑤ ⑥ ⑦ ⑧ ⑨
⓪ ① ② ③ ④ ⑤ ⑥ ⑦ ⑧ ⑨

170) NO RELLENAR
⓪ ① ② ③ ④ ⑤ ⑥ ⑦ ⑧ ⑨
⓪ ① ② ③ ④ ⑤ ⑥ ⑦ ⑧ ⑨

170.- ¿Le extirparon a usted su matríz?

◯ Sí ◯ No

¿Por qué motivo?

◯ Miomas ◯ No sabe
◯ Cáncer ◯ Otro ____________________ (Especifique)

170a) ¿Cuándo fue la fecha de su última regla?

día: ⓪ ① ② ③ / ⓪ ① ② ③ ④ ⑤ ⑥ ⑦ ⑧ ⑨

mes: ⓪ ① ② / ⓪ ① ② ③ ④ ⑤ ⑥ ⑦ ⑧ ⑨

año: ⓪ ① ② / ⓪ ① ② ③ / ⓪ ① ② ③ ④ ⑤ ⑥ ⑦ ⑧ ⑨ / ⓪ ① ② ③ ④ ⑤ ⑥ ⑦ ⑧ ⑨

171.- ¿Alguna vez ha tomado hormonas para la menopausia (como estrógenos o estrógenos más progesterona, por ejemplo: Premarin)?

◯ Sí ◯ No

Por favor indique las razones por las que no ha tomado tratamiento hormonal para la menopausia:

◯ Porque las hormonas no son necesarias ◯ Porque no he tenido molestias
◯ Otra: (Especifique) ____________ ◯ Porque el médico no me las recomendó

171a) NO RELLENAR
⓪ ① ② ③ ④ ⑤ ⑥ ⑦ ⑧ ⑨

¿Por cuántos años tomó los medicamentos señalados, en las edades que se le indican. (Deja en blanco aquellos en los que no los haya tomado)?

Años de Edad	¿Por cuántos años?
20-29 años	⓪ ① ② ③ ④ ⑤ ⑥ ⑦ ⑧ ⑨
30-39 años	⓪ ① ② ③ ④ ⑤ ⑥ ⑦ ⑧ ⑨
40-49 años	⓪ ① ② ③ ④ ⑤ ⑥ ⑦ ⑧ ⑨
50-59 años	⓪ ① ② ③ ④ ⑤ ⑥ ⑦ ⑧ ⑨
60-69 años	⓪ ① ② ③ ④ ⑤ ⑥ ⑦ ⑧ ⑨
70-79 años	⓪ ① ② ③ ④ ⑤ ⑥ ⑦ ⑧ ⑨
80-90 años	⓪ ① ② ③ ④ ⑤ ⑥ ⑦ ⑧ ⑨

¿Por qué razón inició este tratamiento? (Puede contestar más de una opción)

◯ Por prescripción médica
◯ Lo escuché en la T.V. o Radio
◯ Por recomendación de otra persona
◯ Otro: ____________________ (Especifique)

171b) NO RELLENAR
⓪ ① ② ③ ④ ⑤ ⑥ ⑦ ⑧ ⑨

Cuando usted empezó a tomar estos medicamentos ¿Con qué frecuencia tenía sus menstruaciones?

◯ No había tenido menstruación en 12 ó más meses
◯ Tuvo una o más menstruaciones en los últimos 12 meses pero la longitud de los ciclos (días entre regla y regla) era muy irregular

172.- ¿Las siguientes preguntas son acerca de la presencia de bochornos o sudoraciones nocturnas. Estos síntomas los presentan algunas mujeres conforme se acercan a la menopausia, aún cuando siguen reglando. Por lo tanto conteste si ha tenido alguno de estos síntomas.

¿Últimamente ha tenido bochornos?
◯ Sí ◯ No (pase a la siguiente pregunta)

¿Desde hace cuánto tiempo?
◯ Menos de un mes ◯ 1-6 meses ◯ 7-11 meses ◯ 1 año

¿Cuantas veces lo ha presentado?
|__|__| veces
⓪ ① ② ③ ④ ⑤ ⑥ ⑦ ⑧ ⑨
⓪ ① ② ③ ④ ⑤ ⑥ ⑦ ⑧ ⑨

¿Sabe usted que existe un tratamiento hormonal para mejorar los Síntomas de la menopausia?
◯ Sí ◯ No

¿Quiénes de las siguientes personas le han informado a usted sobre este tratamiento?
◯ Su médico familiar ◯ Amigos o familiares
◯ Otro médico ◯ Otro personal de salud
◯ Otro: (Especifique)________________________________

172a) NO RELLENAR
⓪ ① ② ③ ④ ⑤ ⑥ ⑦ ⑧ ⑨

172b) NO RELLENAR
⓪ ① ② ③ ④ ⑤ ⑥ ⑦ ⑧ ⑨

¿Últimamente ha tenido sudoraciones nocturnas?
◯ Sí ◯ No

¿Desde hace cuánto tiempo?
◯ Menos de un mes ◯ 1-6 meses ◯ 7-11 meses ◯ 1 año
◯ 2 años ◯ 3 años ◯ 4 años ◯ 5 años

¿Cuántas veces?
|__|__| veces
⓪ ① ② ③ ④ ⑤ ⑥ ⑦ ⑧ ⑨
⓪ ① ② ③ ④ ⑤ ⑥ ⑦ ⑧ ⑨

¿Estaría dispuesta a utilizar dicho tratamiento si su médico se lo recetara?
◯ No ◯ Sí

Por favor indíquenos cuáles son las causas por las que no utilizaría ese tratamiento
◯ Porque me han dicho que hace daño
◯ Porque causa cáncer
◯ Porque no me gusta tomar medicamento
◯ Porque me da miedo tomar hormonas
◯ Porque no he tenido molestias
◯ Otro: (Especifique)________________________________

173.- Las siguientes preguntas se refieren a la menopausia de su madre y de su hermana mayor. Si no sabe las respuestas trate de averiguarlas ya que son importantes para este estudio.

¿A qué edad tuvo la menopausia su madre?
|__|__| años
⓪ ① ② ③ ④ ⑤ ⑥ ⑦ ⑧ ⑨
⓪ ① ② ③ ④ ⑤ ⑥ ⑦ ⑧ ⑨

La menopausia de su madre fue:
◯ Natural ◯ Por cirugía

¿Tiene alguna hermana? ◯ No ◯ Sí
(Pase a la siguiente pregunta)

¿A qué edad tuvo la menopausia la mayor de sus hermanas?
◯ Aún no ha tenido la menopausia (pase a la siguiente pregunta)
|__|__| años
⓪ ① ② ③ ④ ⑤ ⑥ ⑦ ⑧ ⑨
⓪ ① ② ③ ④ ⑤ ⑥ ⑦ ⑧ ⑨

¿La menopausia de su hermana fue?
◯ Natural ◯ Por cirugía

174) NO RELLENAR
⓪ ① ② ③ ④ ⑤ ⑥ ⑦ ⑧ ⑨
⓪ ① ② ③ ④ ⑤ ⑥ ⑦ ⑧ ⑨

174.- ¿En los últimos 3 meses ha tenido frecuentemente alguna de las siguientes molestias?
◯ Dolor en el vientre
◯ Crecimiento del abdomen
◯ Flujo vaginal (escurrimiento de color blanco o amarillento)
◯ Ardor al orinar
◯ Cuando hace un esfuerzo o se ríe se le sale un poco de orina
◯ Sangrado menstrual más abundante de lo normal
◯ Sangrado entre sus menstruaciones
◯ Dolor y/o atraso de su regla
◯ Fluido vaginal con sangre
◯ Dolor cuando tiene relaciones sexuales
◯ Ninguna
◯ Otro: (Especifique) ________________

¿Acudió al médico por esta(s) molestia(s)?
◯ Sí ◯ No

Nos podría indicar qué tanto vello presenta en las diferentes partes del cuerpo que se muestran a continuación:
(marque el circulo que mejor refleja su condición)

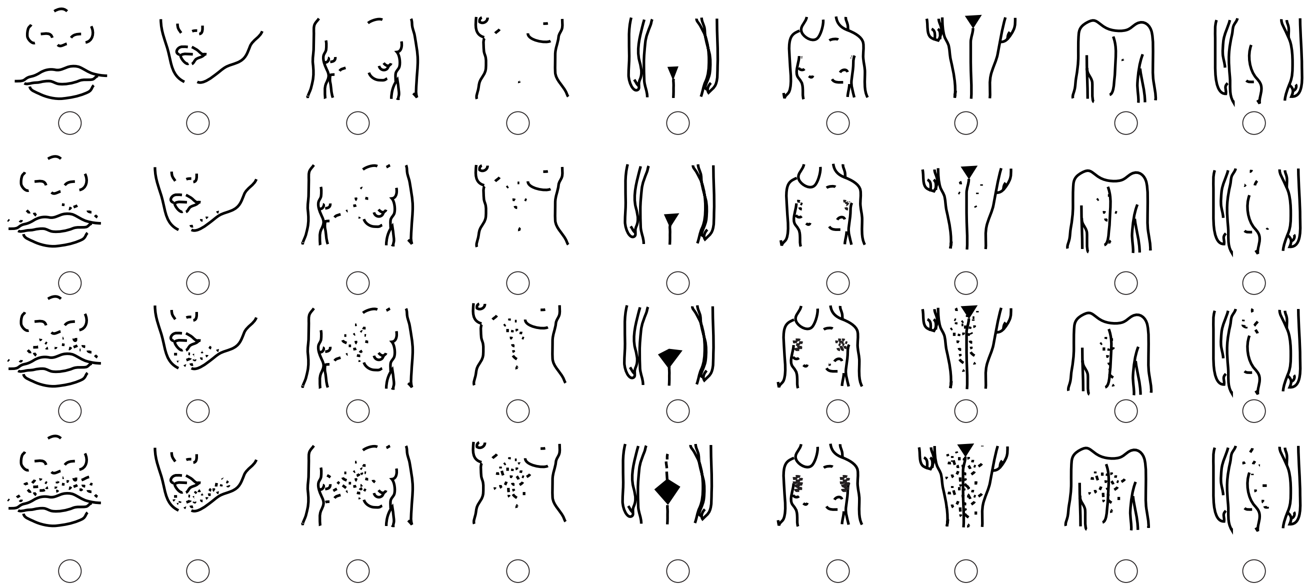

175.- ¿Actualmente utiliza usted suplementos de calcio en su dieta?

◯ Sí → ¿Desde cuándo los toma? mes ⎿⏊⏌ año ⎿⏊⏌
◯ No

175a) NO RELLENAR ⓪ ① ② ③ ④ ⑤ ⑥ ⑦ ⑧ ⑨ | 175b) NO RELLENAR ⓪ ① ② ③ ④ ⑤ ⑥ ⑦ ⑧ ⑨

De la siguiente lista, seleccione cuáles medicamentos consume; en la segunda columna, marque la cantidad de tabletas, comprimidos, grageas o cucharadas que consume en un día. Marque también cuál es la presentación del medicamento, rellenando el círculo que le corresponda a la presentación y dosis de ese medicamento.

NOMBRE DEL MEDICAMENTO	Cantidad que toma al día	PRESENTACIÓN: Tabletas				Comprimidos Masticables		Comprimidos Efervescentes			Grageas		Polvo	
		475 mg	600 mg	950 mg	1 g.	1250 mg	1500 mg	500 mg	1000 mg	3 g.	200 mg	500 mg	1 gr	2 gr
CALCIUM-SANDOZ	⓪ ① ② ③ ④ ⑤	◯	◯	◯	◯	◯	◯	◯	◯	◯	◯	◯	◯	◯
OSSOPAN	⓪ ① ② ③ ④ ⑤	◯	◯	◯	◯	◯	◯	◯	◯	◯	◯	◯	◯	◯
CALCIVAL	⓪ ① ② ③ ④ ⑤	◯	◯	◯	◯	◯	◯	◯	◯	◯	◯	◯	◯	◯
CALCIBRONAT	⓪ ① ② ③ ④ ⑤	◯	◯	◯	◯	◯	◯	◯	◯	◯	◯	◯	◯	◯
CALSAN	⓪ ① ② ③ ④ ⑤	◯	◯	◯	◯	◯	◯	◯	◯	◯	◯	◯	◯	◯
CALSAN2	⓪ ① ② ③ ④ ⑤	◯	◯	◯	◯	◯	◯	◯	◯	◯	◯	◯	◯	◯
CALTRATE 600	⓪ ① ② ③ ④ ⑤	◯	◯	◯	◯	◯	◯	◯	◯	◯	◯	◯	◯	◯

Otro: (Especifique)________________________

Especifique la presentación (Ejemplo de 500 mg.): ________________________

175c) NO RELLENAR ⓪ ① ② ③ ④ ⑤ ⑥ ⑦ ⑧ ⑨ | 175d) NO RELLENAR ⓪ ① ② ③ ④ ⑤ ⑥ ⑦ ⑧ ⑨

Relaciones Familiares

Las siguientes preguntas son incorporadas en el estudio con la finalidad de estudiar el impacto de las relaciones familiares sobre el bienestar de las mujeres. En nuestra cultura la familia es una parte central de la identidad de las mujeres, por lo que cualquier fisura en las relaciones familiares, especialmente con la pareja tiene un gran impacto sobre la salud física y mental. La violencia de pareja es una probable consecuencia de esas fisuras que puede afectar severamente el bienestar de las mujeres. No obstante, a pesar del incremento en el entendimiento de este problema, se conoce muy poco acerca de sus causas, magnitud y consecuencias a largo plazo.

Le recordamos una vez más que la información personal que nos provee es estrictamente confidencial. Por lo cual, la identidad de los participantes es removida de los datos y por tanto para los investigadores esta información es anónima.

Las siguientes preguntas están relacionadas con las conductas de personas en su relación de pareja. Por lo anterior las preguntas están dirigidas a mujeres con pareja, si esta situación aplica a su persona por favor responda a cada una de las preguntas cuidadosamente.

Recuerde que sus respuestas son confidenciales. Si la pareja con la que convive actualmente es distinta a la del año pasado, conteste las siguientes preguntas.

176.- ¿Tiene usted pareja? ◯ Sí ◯ **No (Fin del cuestionario)**

¿Hace cuánto tiempo (anda) o (vive) con su pareja?

meses ⎿⏊⏌ ⓪ ① ② ③ ④ ⑤ ⑥ ⑦ ⑧ ⑨ ⑩ ⑪ ⑫ ⑬ ó más

¿Vive con su pareja?

◯ De tiempo completo
◯ Tres cuartas partes del año o más
◯ La mitad del año
◯ Visita mensual
◯ Visita una o dos veces al año
◯ Otro: ____________________ (Especifique)

176a) NO RELLENAR ⓪ ① ② ③ ④ ⑤ ⑥ ⑦ ⑧ ⑨

¿Tuvo otra pareja antes de la actual?

◯ Sí ◯ No

¿Cuánto tiempo vivió o anduvo con su pareja anterior?

años ⓪ ① ② ③ ④ ⑤ ⑥ ⑦ ⑧ ⑨ ⑩ ⑪ ⑫ ó más

En total ¿cuántas parejas ha tenido en su vida?

⓪ ① ② ③ ④ ⑤ ⑥ ⑦ ⑧ ⑨ ⑩ ó más

¿Cuántos años cumplidos tiene su pareja actual?

años ⎿⏊⏌
⓪ ① ② ③ ④ ⑤ ⑥ ⑦ ⑧ ⑨
⓪ ① ② ③ ④ ⑤ ⑥ ⑦ ⑧ ⑨

¿Su pareja actual tiene otra mujer?

◯ Sí ◯ No ◯ No sé

¿Su pareja actual hasta qué año fue a la escuela?

rellene hasta qué año estudió

◯ Primaria ① ② ③ ④ ⑤ ⑥
◯ Secundaria ① ② ③
◯ Preparatoria ① ② ③
◯ Normal Superior ① ② ③ ④ ⑤ ⑥
◯ Profesional ① ② ③ ④ ⑤ ⑥
◯ Posgrado ① ② ③ ④ ⑤ ⑥

¿Cuando llega su pareja del trabajo, cómo lo siente?

◯ Cansado ◯ Fastidiado
◯ Tenso ◯ Triste
◯ Alegre ◯ Otro: (Especifique)______________

176c) NO RELLENAR ⓪ ① ② ③ ④ ⑤ ⑥ ⑦ ⑧ ⑨

¿Quién de los dos, usted o su pareja, gana más dinero por su trabajo?

◯ Yo ◯ Igual remuneración ◯ Él ◯ No sé

¿Qué opina su pareja de que usted gane más dinero que él?

◯ Él no sabe que gano más ◯ No le importa
◯ Le disgusta un poco ◯ Le disgusta mucho
◯ Le gusta

¿Su pareja está de acuerdo en que usted trabaje fuera del hogar?

◯ No ◯ Sí ◯ No sabe

¿Para usted qué es más importante, el amor de la pareja o de los hijos?

◯ El de la pareja ◯ El de los hijos ◯ Ambos por igual

¿La última vez que su pareja tomó algún tipo de bebida alcohólica, cuántas copas tomó?

Nunca ha tomado	Una copa	Dos copas	Tres copas	Cuatro copas	Cinco copas
◯	◯	◯	◯	◯	◯
**Seis copas**	**Siete copas**	**Ocho a 10 copas**	**11 a 15 copas**	**Más de 15 copas**	**No sabe**
◯	◯	◯	◯	◯	◯

176b) NO RELLENAR ⓪ ① ② ③ ④ ⑤ ⑥ ⑦ ⑧ ⑨

¿A qué se dedica actualmente su pareja?

◯ Obrero ◯ Campesino o jornalero ◯ Desempleado ◯ Empleado
◯ Ayudante, peón o chalán de albañil ◯ Vendedor ambulante ◯ Albañil ◯ Trabaja por su cuenta
◯ Otra: (Especifique)______________________

177.- Las siguientes preguntas están relacionadas con la violencia que todas las mujeres de una u otra manera han sufrido. Hablar de las relaciones violentas que hemos sufrido es algo difícil. Sin embargo, es vital para empezar a cambiar nuestras vidas y mejorar nuestra salud y la de nuestros hijos. No es un examen, no hay respuestas correctas e incorrectas. Responda cada pregunta dependiendo de la frecuencia con que ha sucedido.

**Durante los últimos 12 meses, su pareja**	**Nunca**	**Una vez**	**Varias veces**	**Muchas veces**	**No sucedió en el último año pero si alguna vez en la vida**
¿La ha empujado a propósito?	○	○	○	○	○
¿La ha sacudido, zarandeado o jaloneado?	○	○	○	○	○
¿Le ha torcido el brazo?	○	○	○	○	○
¿La ha jaloneado del cabello?	○	○	○	○	○
¿La ha golpeado con la mano o el puño?	○	○	○	○	○
¿La ha pateado?	○	○	○	○	○
¿Le ha golpeado el estómago?	○	○	○	○	○
¿Le ha aventado algún objeto?	○	○	○	○	○
Especifique ____________________					
¿Le ha encerrado bajo llave?	○	○	○	○	○
¿La ha amarrado?	○	○	○	○	○
¿La ha golpeado con algún palo, cinturón o algún objeto doméstico?	○	○	○	○	○
¿Le ha quemado con un cigarro?	○	○	○	○	○
¿La ha tratado de asfixiar?	○	○	○	○	○
¿La ha agredido con alguna navaja, cuchillo o machete?	○	○	○	○	○
¿Le ha disparado con una pistola o rifle?	○	○	○	○	○
¿La rebaja o menosprecia?	○	○	○	○	○
¿La insulta?	○	○	○	○	○
¿Se puso celoso o sospecha de sus amistades?	○	○	○	○	○
¿Le dice cosas como que esta poco atractiva o fea?	○	○	○	○	○
¿Se puso a golpear o patear la pared o algún mueble?	○	○	○	○	○
¿Se puso a destruir alguna de sus cosas?	○	○	○	○	○
¿La amenazó con golpearla?	○	○	○	○	○
¿La amenazó con alguna navaja, cuchillo o machete?	○	○	○	○	○
¿La amenazó con alguna pistola o rifle?	○	○	○	○	○
¿Le hizo sentir miedo de él?	○	○	○	○	○
¿La amenazó con matarla?	○	○	○	○	○
¿La amenazó con matarse a si mismo?	○	○	○	○	○
¿Se opone a que trabaje o estudie?	○	○	○	○	○
¿Siente usted que la controla o vigila?	○	○	○	○	○
¿Le impide que visite a sus amistades o familiares?	○	○	○	○	○
¿Le ha exigido tener relaciones sexuales aunque usted no este dispuesta?	○	○	○	○	○
¿La amenaza con irse con otras mujeres si no accede a tener relaciones sexuales con él?	○	○	○	○	○
¿Usa la fuerza física para tener relaciones sexuales con usted en contra	○	○	○	○	○
de su voluntad?	○	○	○	○	○
¿La obliga a tener sexo oral en contra de su voluntad?	○	○	○	○	○
¿La obliga a tener sexo anal en contra de su voluntad?	○	○	○	○	○
Otra: (Especifique) ______________________________	○	○	○	○	○

177a) NO RELLENAR
⓪ ① ② ③ ④ ⑤ ⑥ ⑦ ⑧ ⑨
⓪ ① ② ③ ④ ⑤ ⑥ ⑦ ⑧ ⑨

177b) NO RELLENAR
⓪ ① ② ③ ④ ⑤ ⑥ ⑦ ⑧ ⑨
⓪ ① ② ③ ④ ⑤ ⑥ ⑦ ⑧ ⑨

178.- ¿Durante los últimos 2 años, alguna persona la ha obligado a tener relaciones sexuales en contra de su voluntad?

○ No
○ Sí → **¿Cuántas veces le ha sucedido esto?** |__|__| veces
⓪ ① ② ③ ④ ⑤ ⑥ ⑦ ⑧ ⑨ o más

¿Quién la ha obligado? (puede contestar varias opciones)

○ Amigo ○ Novio ○ Padre ○ Esposo
○ Tío ○ Padrastro ○ Desconocido ○ Otro: (Especifique) ______________

178) NO RELLENAR
⓪ ① ② ③ ④ ⑤ ⑥ ⑦ ⑧ ⑨

179.- Si en la sección previa contestó que ha sufrido golpes o heridas por favor conteste las siguientes preguntas

	Sí	No
¿Tuvo moretones?	◯	◯
¿Tuvo cortadas que le hayan tenido que coser o suturar?	◯	◯
¿Tuvo hemorragias por la nariz?	◯	◯
¿Tuvo fractura de algún hueso?	◯	◯
¿Tuvo quemaduras?	◯	◯
¿Perdió algún diente?	◯	◯

	Sí	No
¿Tuvo lesiones internas de algún órgano?	◯	◯
¿Quedó desmayada o inconsciente?	◯	◯
¿Le quedaron dolores en el cuerpo por algunos días?	◯	◯
¿Perdió el uso de la función de alguna parte de su cuerpo?	◯	◯
¿Tuvo que recibir atención médica?	◯	◯
¿Tuvo que hospitalizarse?	◯	◯

¿En qué parte del cuerpo fue golpeada?
(marque con una X la zona afectada)

179a) NO RELLENAR
⓪ ① ② ③ ④ ⑤ ⑥ ⑦ ⑧ ⑨
⓪ ① ② ③ ④ ⑤ ⑥ ⑦ ⑧ ⑨
⓪ ① ② ③ ④ ⑤ ⑥ ⑦ ⑧ ⑨

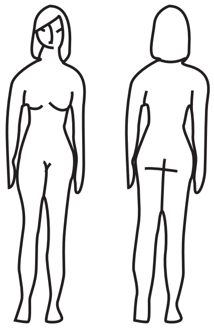

Durante el último año:

¿Su pareja alguna vez la golpeó estando embarazada?

◯ Sí ◯ No ◯ No he estado embarazada

¿Su pareja la ha maltratado delante de sus hijos?
◯ Sí ◯ No ◯ No tengo hijos

¿Su pareja la ha maltratado cuando está bajo el efecto del alcohol?
◯ Sí ◯ No ◯ No toma

¿Cuando su pareja la maltrató la última vez, mencione cómo empezó todo? (Descríbalo con sus propias palabras)

____
____
____

¿Por qué cosas discute más frecuentemente con su pareja?
(Descríbalo con sus propias palabras)

____
____
____

179b) NO RELLENAR
⓪ ① ② ③ ④ ⑤ ⑥ ⑦ ⑧ ⑨

179c) NO RELLENAR
⓪ ① ② ③ ④ ⑤ ⑥ ⑦ ⑧ ⑨

**180.- En caso de haber sufrido algún tipo de agresión por parte se su pareja nos gustaría saber cómo se siente ahora sobre este problema. Para esto responda las siguientes preguntas**	Siempre	Casi siempre	Frecuente-mente	En ocasiones	Casi nunca	Nunca
¿Se siente asustada, temerosa o molesta cuando piensa en lo que ocurrió?	◯	◯	◯	◯	◯	◯
¿Le vuelve a la mente, esto es, recuerda imágenes, sonidos, etc. de lo que ocurrió?	◯	◯	◯	◯	◯	◯
¿Los recuerdos de lo que ocurrió le vuelven a la mente cuando usted no quiere?	◯	◯	◯	◯	◯	◯
¿Tiene sueños o pesadillas sobre lo que ocurrió?	◯	◯	◯	◯	◯	◯
¿Piensa que algo similar a lo que le ocurrió le pasará de nuevo?	◯	◯	◯	◯	◯	◯
¿Se siente más sola desde el momento en que le ocurrió este problema?	◯	◯	◯	◯	◯	◯
¿Se siente tan temerosa o triste por lo que le ocurrió que ya no quiere pensar como se siente?	◯	◯	◯	◯	◯	◯
¿Se siente tan temerosa o triste por lo que ocurrió que ya no quiere ni hablar o llorar?	◯	◯	◯	◯	◯	◯
¿Se siente tan inestable o nerviosa desde que le ocurrió este problema?	◯	◯	◯	◯	◯	◯
¿Duerme bien desde que le ocurrió este problema?	◯	◯	◯	◯	◯	◯
¿Se siente mal o culpable porque no hizo algo que quiso o debió hacer para evitar que le ocurriera este problema?	◯	◯	◯	◯	◯	◯
¿Puede usted poner atención o concentrarse como antes de que le ocurriera este problema?	◯	◯	◯	◯	◯	◯
¿Quiere estar lejos de las cosas o lugares que le recuerdan lo que le ocurrió?	◯	◯	◯	◯	◯	◯
¿Se pone tensa o molesta cuando algo le recuerda o le hace pensar en lo que le ocurrió?	◯	◯	◯	◯	◯	◯
¿Esta teniendo molestias como dolor de estómago o dolor de cabeza más frecuente que antes que le ocurriera este problema?	◯	◯	◯	◯	◯	◯

181.- Enliste las 3 cosas que mejor le han funcionado para mantenerse a salvo de las agresiones de su pareja:

181a) NO RELLENAR
⓪ ① ② ③ ④ ⑤ ⑥ ⑦ ⑧ ⑨

181b) NO RELLENAR
⓪ ① ② ③ ④ ⑤ ⑥ ⑦ ⑧ ⑨

181c) NO RELLENAR
⓪ ① ② ③ ④ ⑤ ⑥ ⑦ ⑧ ⑨

1 ____

2 ____

3 ____

182.- Hay muchas formas en las que las mujeres tratan de evitar o intentan protegerse contra la violencia o de las amenazas de sus parejas. Algunas de estas acciones pueden ser útiles para evitar las agresiones o las amenazas de su pareja, pero pudiera ser que otras simplemente hagan que las cosas empeoren. ¿Alguna vez ha hecho alguna de las siguientes cosas, si su respuesta es "Sí", haciendo memoria, por favor indique qué tan útil fue la acción para terminar, detener o escapar de la violencia de su pareja?

**Acciones que he tomado o cosas que he tenido que hacer para evitar la violencia de mi pareja**	Sí	No	¿Qué tan útil le resultó haber hecho esto? Poco útil ←→ Muy útil					
He tratado de hablar con él acerca de su violencia	◯	◯	0	1	2	3	4	5
He peleado con él físicamente	◯	◯	0	1	2	3	4	5
He elegido dormir en otra cama	◯	◯	0	1	2	3	4	5
He dejado la casa para alejarme de él	◯	◯	0	1	2	3	4	5
Me he negado a hacer lo que él dice	◯	◯	0	1	2	3	4	5
He terminado (o tratado de terminar) la relación	◯	◯	0	1	2	3	4	5
He usado un arma en su contra o lo he amenazado con un arma	◯	◯	0	1	2	3	4	5
He tratado de no llorar durante la agresión	◯	◯	0	1	2	3	4	5
He tratado de mantener las cosas con calma para él	◯	◯	0	1	2	3	4	5
He puesto un cuchillo, una pistola o alguna otra arma donde la pueda alcanzar en caso de necesitarla para protegerme de él	◯	◯	0	1	2	3	4	5
He mantenido las llaves del coche o de la casa lejos de su alcance	◯	◯	0	1	2	3	4	5
He escondido dinero u objetos de valor	◯	◯	0	1	2	3	4	5
He hecho todo lo que él quiere con tal de detener o prevenir la violencia	◯	◯	0	1	2	3	4	5
He desarrollado un código con otras personas para hacerles saber cuando estoy en peligro	◯	◯	0	1	2	3	4	5
He diseñado un plan de escape	◯	◯	0	1	2	3	4	5
He sacado armas de la casa o las he escondido para que él no tenga acceso a ellas	◯	◯	0	1	2	3	4	5
He guardado números de teléfonos importantes para pedir ayuda	◯	◯	0	1	2	3	4	5
He guardado provisiones adicionales (comida o medicinas) para mí y/o para mis hijos	◯	◯	0	1	2	3	4	5
He escondido papeles importantes (pólizas de seguros o estados de cuenta bancario)	◯	◯	0	1	2	3	4	5
He mandado a los niños a que se queden con un amigo o un familiar cuando él se pone violento.	◯	◯	0	1	2	3	4	5

183.- En ocasiones, existen dificultades entre la pareja con respecto a la manera en que se llevan los gastos de la familia o el manejo del dinero. Lea con atención las siguientes preguntas y rellene el círculo de la opción que corresponda mejor a su situación

**Su pareja:**	**Una vez**	**Pocas veces**	**Muchas veces**	**Nunca**
Le ha reclamado cómo gasta usted el dinero	◯	◯	◯	◯
Aunque tenga dinero ha sido codo o tacaño con los gastos de la casa	◯	◯	◯	◯
La ha amenazado diciendo que no le va a dar gasto o no le da	◯	◯	◯	◯
Se ha gastado el dinero que se necesita para la casa	◯	◯	◯	◯
Se ha adueñado o le ha quitado bienes (cosas, terrenos, etc.)	◯	◯	◯	◯
Le ha prohibido trabajar o estudiar	◯	◯	◯	◯

3a forros
adultos

Fin del cuestionario

Gracias por su participación

Contra Portada
IMSS adultos

Unidad de Investigación Epidemiológica y en Servicios de Salud
Av. Plan de Ayala Esq. Calle Central s/n Col. Chapultepec Cuernavaca, Morelos C.P. 62450
Teléfono: 100 0855 Email: uiess@hotmail.com
